# Lateral Flow Glyco-Assays for the Rapid and Low-Cost Detection of Lectins–Polymeric Linkers and Particle Engineering Are Essential for Selectivity and Performance

**DOI:** 10.1002/adhm.202101784

**Published:** 2021-11-17

**Authors:** Alexander N. Baker, Asier R. Muguruza, Sarah-Jane Richards, Panagiotis G. Georgiou, Stephen Goetz, Marc Walker, Simone Dedola, Robert A. Field, Matthew I. Gibson

**Affiliations:** Department of Chemistry, University of Warwick, Gibbet Hill Road, Coventry CV4 7AL, UK; Department of Chemistry, University of Warwick, Gibbet Hill Road, Coventry CV4 7AL, UK; School of Chemistry, University of Birmingham, Edgbaston, Birmingham B15 2TT, UK; Department of Chemistry, University of Warwick, Gibbet Hill Road, Coventry CV4 7AL, UK; Iceni Diagnostics Ltd, Norwich Research Park, Norwich NR4 7GJ, UK; Department of Physics, University of Warwick, Gibbet Hill Road, Coventry CV4 7AL, UK; Iceni Diagnostics Ltd, Norwich Research Park, Norwich NR4 7GJ, UK; Department of Chemistry and Manchester Institute of Biotechnology, University of Manchester, Manchester M1 7DN, UK; Department of Chemistry, University of Warwick, Gibbet Hill Road, Coventry CV4 7AL, UK; Warwick Medical School, University of Warwick, Gibbet Hill Road, Coventry CV4 7AL, UK

**Keywords:** carbohydrates, diagnostics, glyco-assays, gold nanoparticles, lateral flow devices, lectins, polymers

## Abstract

Lateral flow immuno-assays, such as the home pregnancy test, are rapid point-of-care diagnostics that use antibody-coated nanoparticles to bind antigens/analytes (e.g., viruses, toxins or hormones). Ease of use, no need for centralized infrastructure and low-cost, makes these devices appealing for rapid disease identification, especially in low-resource environments. Here glycosylated polymer-coated nanoparticles are demonstrated for the sensitive, label-free detection of lectins in lateral flow and flow-through. The systems introduced here use glycans, not antibodies, to provide recognition: a “lateral flow glyco-assay,” providing unique biosensing opportunities. Glycans are installed onto polymer termini and immobilized onto gold nanoparticles, providing colloidal stability but crucially also introducing assay tunability and selectivity. Using soybean agglutinin and *Ricinus communis* agglutinin I (RCA_120_) as model analytes, the impact of polymer chain length and nanoparticle core size are evaluated, with chain length found to have a significant effect on signal generation—highlighting the need to control the macromolecular architecture to tune response. With optimized systems, lectins are detectable at subnanomolar concentrations, comparable to antibody-based systems. Complete lateral flow devices are also assembled to show how these devices can be deployed in the “real world.” This work shows that glycan-binding can be a valuable tool in rapid diagnostics.

## 1 Introduction

Lateral flow devices (LFDs), such as the home pregnancy test,^[1]^ can be used to provide rapid point of care testing at low cost. The cost-effectiveness and clinical usefulness of LFDs has been well demonstrated by malaria rapid diagnostic tests,^[2,3]^ in the diagnosis of cutaneous leishmaniasis^[4]^ and in comparisons with reverse transcription polymerase chain reaction (RT-PCR) approaches for Ebola diagnosis.^[5]^ More recently LFDs have been used to detect SARS-COV-2, as rapid and low-cost diagnostics allowing for early detection when deployed appropriately.^[6]^ LFDs are chromatographic paper-based devices which function by flowing the analyte past a functionalized stationary phase with affinity for the analyte. As the analyte passes through the device it is bound by both the stationary phase and the signal generating mobile phase, most commonly gold nanoparticles (AuNPs) functionalized with receptors for the analyte, “sandwiching” the analyte.^[7]^ This leads to a visible color forming at the test line, indicating a positive test. AuNPs are the most commonly^[7]^ used mobile phase due to their strong coloration associated with their localized surface plasmon resonance band,^[8–10]^ and ease of functionalization by nonspecific surface passivation (e.g., absorption of proteins), or through aurophilic functionalities such as thiols. AuNPs are also easy to synthesize by reduction of gold salts. Notably, other signal generating units such as; quantum dots,^[11]^ graphene oxide,^[12,13]^ and carbon nanotubes^[14]^ have also been used in LFDs.

Typically, the capture units for the analyte, on both the test line and nanoparticle surface, are antibodies, owing to their high affinity and selectivity. However, antibodies are not essential components in LFDs. Other recognition units such as; nucleic acids,^[15]^ lectins,^[16]^ and glycans can also be used, so long as the analyte is bound with sufficient affinity and specificity. Glycans are ubiquitous in biological systems^[17]^ with over half of all mammalian proteins estimated to be glycosylated^[18]^ and glycoconjugates playing a variety of roles from cell signaling^[19]^ to mediating immune responses.^[20]^ They are also the site of pathogen adhesion during many viral infections,^[21,22]^ especially respiratory viruses such as influenzas.^[23]^

The diverse range of biological recognition processes driven by glycans presents many opportunities to either target the glycans themselves or the proteins that sense for them (lectins) in biosensing or diagnostic applications. Lectins are found in a wide-array of environments, for example the cholera toxin,^[24]^ ricin,^[25]^ lectins in snake venoms^[26]^ and as biocides in algae.^[27]^ Furthermore, lectins have been used for decades as histological stains, to identify diseased tissue based on glycosylation,^[28]^ while lectin-containing biosensors have been extensively reviewed.^[29–31]%^ Damborský et al. have reported a LFD that utilizes immobilized lectins (in place of antibodies) as test lines for prostate specific antigen,^[16,32]^ and Bayoumy et al. have used antibodies to target glycans.^[33]^ However, to the best of our knowledge, there are very few examples of the exploration of glycans as the detection units in lateral flow, that is, using glycans to sense for an analyte—rather than targeting glycans as an analyte or a glycosylated analyte. A mannose-functionalized *p*-acrylamidophenyl polymer-coated AuNP, with an antibody as the test line, was used by Ishii et al. to detect Concanavalin A (ConA) in a LFD.^[34]^ We believe this is the first report of glycans forming part of an LFD. Miura and coworkers built on this work further by preparing a small panel of glycopolymer functionalized AuNPs for the detection of ConA—again using an antibody as the stationary phase.^[35]^ These two examples demonstrated that glyco-nanoparticles could be deployed in LFDs, however, both reports relied on using antibodies in part, and were only demonstrated against the plant lectin ConA. Baker et al., discovered that SARS-COV-2 (the causative agent of COVID-19) spike protein has affinity toward sialic acids,^[36]^ as had been reported for previous coronaviruses^[37,38]^ including the coronavirus that causes Middle East respiratory syndrome.^[39]^ Using *N*-acetyl neuraminic acid-terminated polymer ligands, immobilized onto AuNPs, it was demonstrated that a SARS-COV-2 spike protein bearing pseudovirus could be detected in a lateral flow glycoassay (using a BSA-glycoconjugate test-line), and that a flowthrough assay (LFD without a test line) device could be used for the detection of S1 spike protein.^[36]^ This clearly demonstrated that lateral flow glyco-assays, LFDs that use glycans as capture agents (on the test line and particle) for an analyte, have potential applications in rapid diagnostics, surveillance, and as accessible research tools for evaluating glycan-binding protein function. Further work utilizing a flow-through glyco-assay demonstrated that glycans could be used as capture agents to sense for the SARS-COV-2 virus in patient samples and that these tests were thermally robust,^[40]^ which could be an advantage of glycan-based devices versus antibody-based devices.^[41]^ To advance the study of glyco-LFD technology, LFDs that use glycans as capture agents on test lines and/or particles, it is crucial to understand how each component (particle, surface) impacts performance.

Herein, we explore how the role of polymer chain length, glycan density, and nanoparticle size affect the performance of lateral flow glyco-assays, for the detection of lectin analytes, as a model system to further validate glyco-LFDs. This study reveals that the outputs (signal, nonspecific binding, and background) were dependent on the nanoparticle’s structural parameters. In particular, the precise chain length of the polymeric tether required for optimal detection of different lectins (soybean agglutinin [SBA] and RCA_120_ [*Ricinus communis* agglutinin I]) was shown to be different. This provides the opportunity to introduce selectivity not just through the glycan, but also through macromolecular engineering, which is a unique feature ofthis technology. Guided by these results, complete diagnostic devices were fabricated and used to detect SBA in 10 min at concentrations as low as 5 μg mL^−1^.

## 2 Results and Discussion

**Figure 1:**
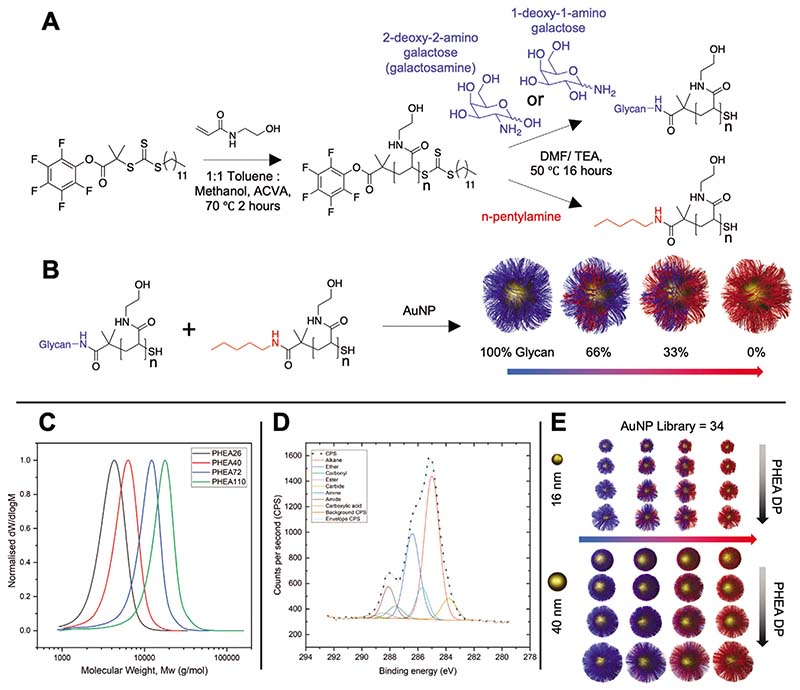
Synthesis of gold nanoparticle library functionalized with glycan-terminated polymeric tethers at various densities. A) Polymerization of *N*-hydroxyethyl acrylamide (HEA) by RAFT, followed by displacement of the PFP ester with amino-glycans. B) Assembly of polymers onto preformed gold nanoparticles to give variable glycan densities. C) Normalized size exclusion chromatography analysis of PHEA polymers from Table 1. D) C 1*s* x-ray photoelectron spectrum of 100% GalPHEA_72_ @AuNP_16_. E) Graphical representation of AuNP library illustrating the three variables of diameter, coating DP, and glycan density.

The primary aim of this work was to develop lateral flow technology to enable the sensitive detection of lectins, using glycosylated polymer-stabilized AuNPs, as an alternative to traditional antibody-based detection systems. To achieve this, an understanding of how particle/polymer structure impacts lateral flow performance was required. Therefore, a library-based screening approach was undertaken, with SBA chosen as the model lectin for detection. The precise chain length, surface glycan density, and particle size have been previously shown to be crucial in plasmonic (aggregation) glyco-assays, by modulating particle/analyte interactions and outcomes, while also ensuring colloidal stability in complex media.^[42,43]^ Reversible addition–fragmentation chain transfer (RAFT) polymerization was used to synthesize a panel of poly(hydroxyethyl acrylamide)s (PHEA) using pentafluorophenyl-2-(dodecylthiocarbonothioylthio)-2-methylpropanoate (PFP-DMP) as the RAFT agent to install a pentafluorophenyl group at the *ω*-chain end, and a protected thiol at the *α*-end (for AuNP immobilization), Figure 1. PHEA was chosen because of its solubility and colloidal stability when immobilized onto AuNPs.^[44]^ The polymers were characterized (Table 1) by size exclusion chromatography (SEC, Figure 1C) showing low dispersity values, and the structure confirmed by ^1^H,^13^C, and ^19^F NMR (Supporting Information). Galactosamine (2-deoxy-2-amino-galactose) was conjugated to the polymer by displacement of the PFP end-group, to mimic the structure of *N*-acetylgalactosamine (GalNAc) due to formation of the amide linkage. Glycan addition was confirmed by FTIR, ^19^F, and ^1^H NMR. Non-glycosylated polymers were produced by reaction with n-pentylamine and used (below) to dilute the density of glycans on the particle surface.

Citrate stabilized 16 and 40 nm AuNPs were synthesized by a seeded growth approach and characterized by dynamic light scattering (DLS), transmission electron microscopy (Figure S12, Supporting Information), and UV–vis analysis.^[45,46]^ The AuNPs were then functionalized with varying ratios of glycosylated and non-glycosylated polymers to produce 100%, 66%, 33%, and 0% glycan-densities on the AuNPs for each polymer length and AuNP size, to produce a library of 34 particles (including the two citrate-stabilized AuNPs), Figure 1. UV–vis spectroscopy and DLS confirmed functionalization (Figures S13–S21 and Table S1, Supporting Information). In some cases, the particles were unstable (fully aggregated): all GalPHEA_26_@AuNP_40_’s and all GalPHEA_40_@AuNP_40_’s except 100% sugar functionalized; hence these particles were excluded from further analysis. It is worth noting that a hydrophobic amine was used in place of the glycan for the nonglycosylated polymers (to remove the PFP group) which contributes to the observed aggregation. A hydrophobic amine was chosen as some aggregation of the particles with both antigen and test line in an LFD may aid detection, and therefore even the dispersed samples showed some populations of larger particles in the DLS (see Supporting Information) but were all suitable for this screening step. Therefore, this does not prevent their application here where the LFD performance is the primary outcome. To further characterize the surface of the particles, x-ray photoelectron spectroscopy (XPS) was conducted on dried particles (Figures S36–S46 and Tables S15 and S16, Supporting Information). XPS confirmed the presence of amide (C(O)NC) and amine (C(O)NC) peaks in the C 1*s* (Figure 1D), and in the N 1*s* scans (amine and amides have similar/overlapping binding energies so were not distinguishable), showing the presence of the PHEA, which were not present in the naked AuNP samples. Similarly, ether (XPS cannot easily distinguish ether from alcohol and are combined in the model employed here) peaks in the C 1*s* scans were far larger in samples containing 100% sugar than in the citrate-stabilized AuNPs with no polymer functionalization. It is important to note the presence of carbonyls and carboxylic acid carbons are from atmospheric contaminants, and the presence of carbide likely from the silicon wafer particle interface.

**Figure 2:**
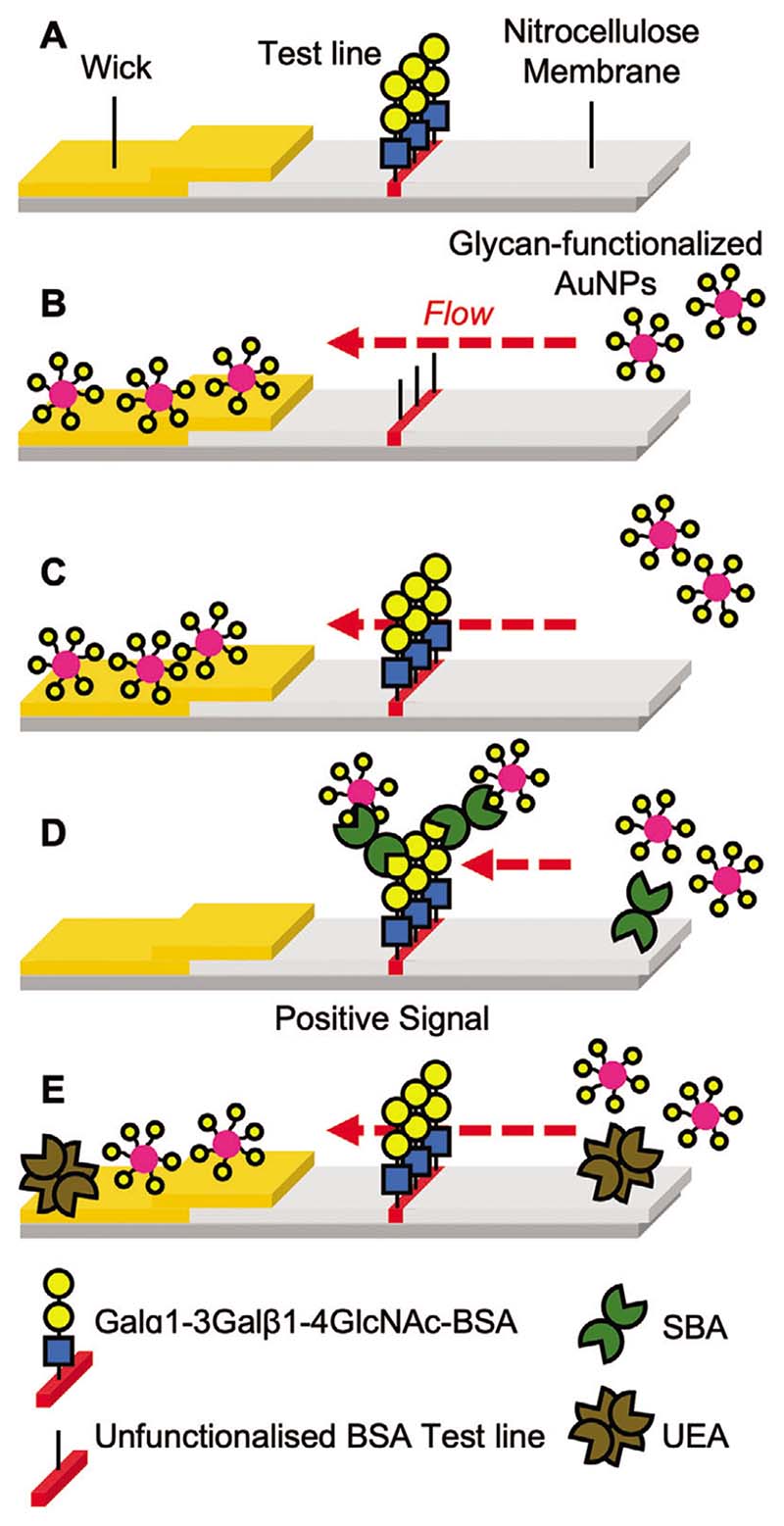
Schematic of dipstick lateral flow assay. A) Design of dipstick. B) Lateral flow with unfunctionalized BSA where particles flow without engaging the test line. C) Lateral flow with Galαl-3Gal*β*1-4GlcNAc-BSA test line and no analyte; particles do not engage test line. D) Lateral flow with Galαl-3Gal*β*1-4GlcNAc-BSA test line and SBA (analyte) resulting in capture and signal generation. E) Lateral flow with Galαl-3Gal*β*1-4GlcNAc-BSA test line and UEA (negative control), hence no signal generation.

With this library of glycoparticles to hand, their function was screened in a lateral flow assay. Figure 2 shows the setup of the assay. A dipstick was made, where the test line (to capture the lectin analyte) was made by depositing 1 μL of 1 mg mL^−1^ Galα1-3Gal*β*1-4GlcNAc-bovine serum albumin conjugate (Galα1-3Gal*β*1-4GlcNAc-BSA) which has affinity for SBA (Figure 2A). For this evaluation no control line was employed, which would be essential for a real diagnostic to demonstrate a device is functioning (and is used in the final devices at the end of this study, below).^[47]^ The mobile phase was SBA (0.05 mg mL^−1^, ≈0.4 nmol mL^−1^) and OD = 1 (optical density at UV_max_, the standard measurement for concentration) AuNPs (Figure 2D). Notably the OD used was kept constant (OD = 1) for all dipsticks and devices to provide a constant concentration across and between assays allowing for easy comparison. Negative controls were run of the AuNPs versus Galα1-3Gal*β*1-4GlcNAc-BSA only (Figure 2C) and unfunctionalized BSA only (Figure 2B) test lines to determine if any off-target binding to the test line itself occurred. Further negative controls were run using AuNPs versus Galα1-3Gal*β*1-4GlcNAc-BSA test lines with *Ulex Europaeus* Agglutinin I (UEA, 0.05 mg mL^−1^, Figure 2E), a lectin with no affinity for GalNAc.

**Figure 3:**
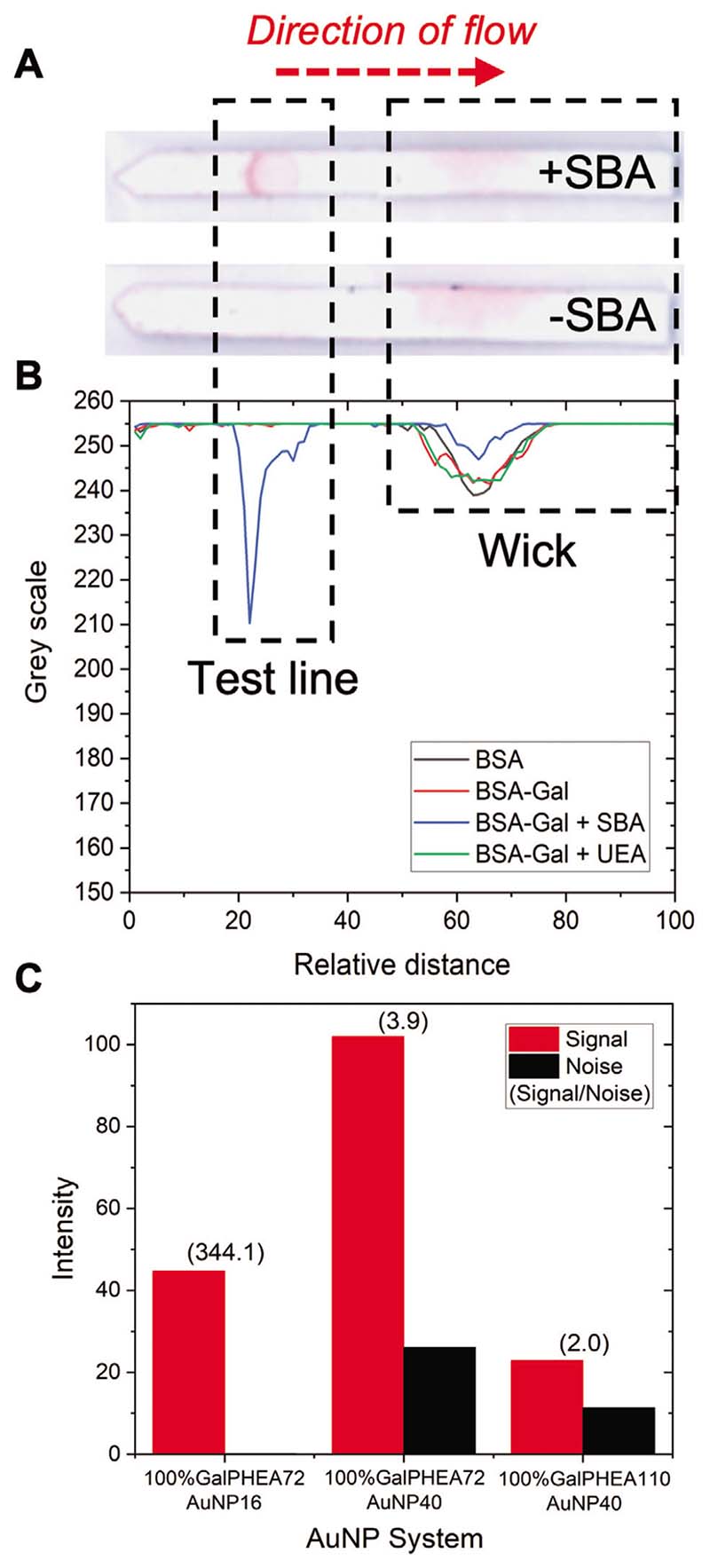
Optimization of the gold nanoparticle in dipstick format using SBA as the analyte. A) Example lateral flow dipsticks showing test line (Galα1-3Gal*β*1-4GlcNAc-BSA, 1 mg mL^−1^) and direction of flow. B) Example image analysis result using 100% GalPHEA_72_@AuNP_16_. C) Summary of selected nanoparticle performance from image analysis. Signal to noise ratio is indicated above each pair of bars. Images shown have been enhanced for clarity and all original dipstick photos and image analyses are included in the Supporting Information. Test lines for (B) are unfunctionalized BSA (BSA, 1 mg mL^−1^), and Galα1-3Gal*β*1-4GlcNAc-BSA (BSA-Gal, 1 mg mL^−1^) with (or without) lectins in solution (SBA or UEA, 0.05 mg mL^−1^).

All dipsticks were run in triplicate for 20 min before being scanned and analyzed with image analysis software^[48]^ to evaluate binding (photographs and image analysis of all strips are in the Tables S2–S12 and Figures S22–S32, Supporting Information). This process of running in triplicate and averaging (mean) the data was carried out for all dipsticks and devices in this study. The test line is situated on the strip around 15 to 35 relative distance units (i.e., *x*-axis output from image analysis) along the strip, noting that the strip length is set to 100 relative distance units. An example of positive (with SBA as analyte) and negative (buffer alone) dipsticks are shown in Figure 3A, with the direction of flow, test line area, and the wick area labeled. The wick area is where unbound nanoparticles gather (at the end of the assay) and is typically “hidden” in the housing of a full lateral flow cassette. An example image analysis of these dipsticks is shown in Figure 3B and a summary of the best performing systems is show in Figure 3C. Full analysis of all strips as a function of nanoparticle composition and original images are included in the Supporting Information.

Consideration of the data revealed three trends; i) as polymer length increases the total amount of binding to SBA decreases, but the nonspecific binding in negative controls was also reduced; ii) decreasing the density of the glycan on the particles decreases binding to SBA but also leads to some increases in nonspecific binding; and iii) increasing AuNP diameter led to increased signal intensity but also increased noise from the background. Taking this into account, the particles that gave optimal performance against SBA were 100% glycan-functionalized GalPHEA_72_@AuNP_16_ and GalPHEA_72_@AuNP_40_. While GalPHEA_110_@AuNP_40_ gave higher signals, the background signal was also very high. These three particle systems were further analyzed by considering their signal to noise ratios (Figure 3C, and Figures S27 and S32 and Tables S7 and S12, Supporting Information); 100% glycan-functionalized GalPHEA_72_@AuNP_16_ was found to have the highest signal to noise ratio despite producing less signal than 100% glycan-functionalized GalPHEA_72_ @AuNP_40_.

There are limited examples of lateral flow assays based only on glycans, but in our previous report of a system for SARS-COV-2 detection, larger nanoparticles (35 nm) were optimal.^[36]^ This highlights how each system can be fine-tuned to the detection challenge, with this data illustrating how tuning the particle/polymer/ligand interfaces enables modulation of the observable outputs. Notably buffer conditions, and materials used in the LFD were kept constant in this work but could also be further optimized to modulate output.

**Figure 4:**
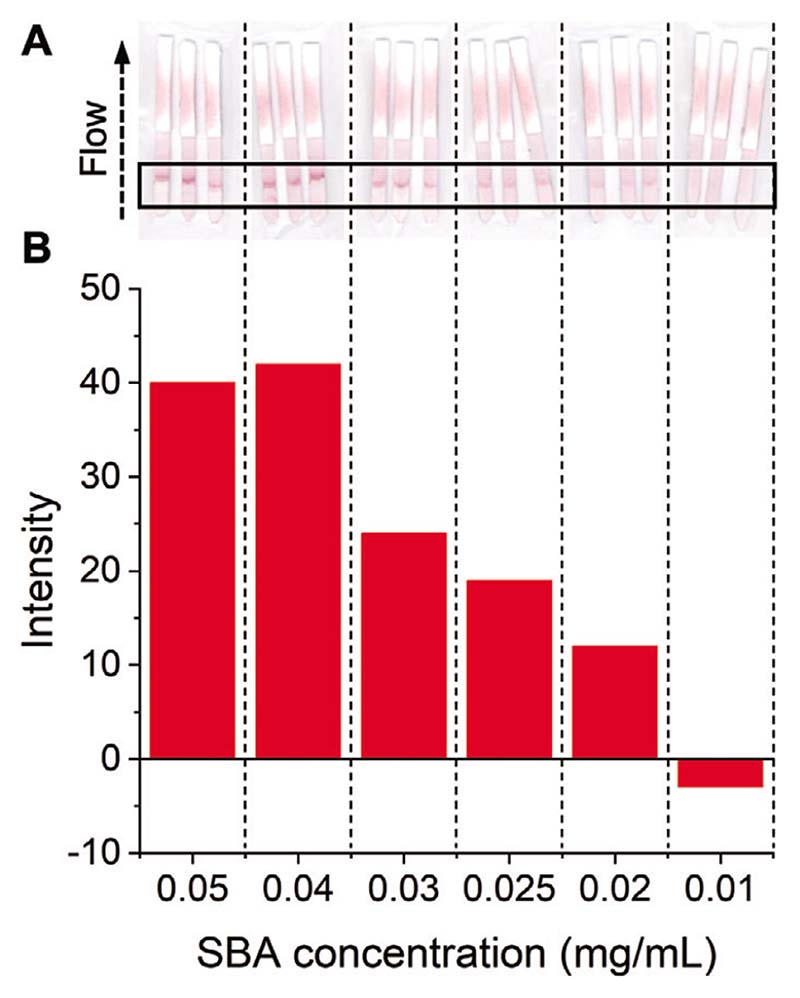
Lateral flow data from SBA dipstick assays to determine limit of detection. A) Lateral flow dipsticks run with the indicated concentrations of SBA using 100% GalPHEA_72_@AuNP_16_. B) Analyzed lateral flow intensity data from the lateral flow strips in A.

The identified optimum particle, 100% glycan-functionalized GalPHEA_72_ @AuNP_16_, was next explored for its limit of detection (LoD) in the dipstick assays. A serial dilution of SBA was prepared in the buffer and run, Figure 4 (Table S13 and Figure S33, Supporting Information). The LoD was found to be 0.02 mg mL^−1^ (0.17 nmol mL^−1^). This is similar to a commercial pregnancy test (≈0.7–0.07 nmol mL^−1^)^[49]^ showing that glycans can achieve the necessary LoD to be a viable alternative/companion, to antibody-based LFDs. It should be noted that no attempts to reduce background (via buffer tuning) were made here, but a lower background was achieved in the final device (below).

The above data showed that the lateral flow glyco-assay approach can be used to detect SBA and that the exact nanoparticle used (size, coating, and density of ligands) can be easily tuned and is a key determinant in their output. Therefore, another lectin was also explored, RCA_120_, which has affinity toward galactose and GalNAc.^[50,51]^ PHEA_40_, PHEA_72_, and PHEA_110_ were functionalized with 1-deoxy-1-amino-galactose due to known affinity of this isomer toward RCA_120_ (Note, this is a different galactosamine isomer than used for the SBA study above). Shorter polymers, less than 100% sugar functionalization and 40 nm AuNPs were not explored based on the experiments with SBA where there was significant particle aggregation.

**Figure 5:**
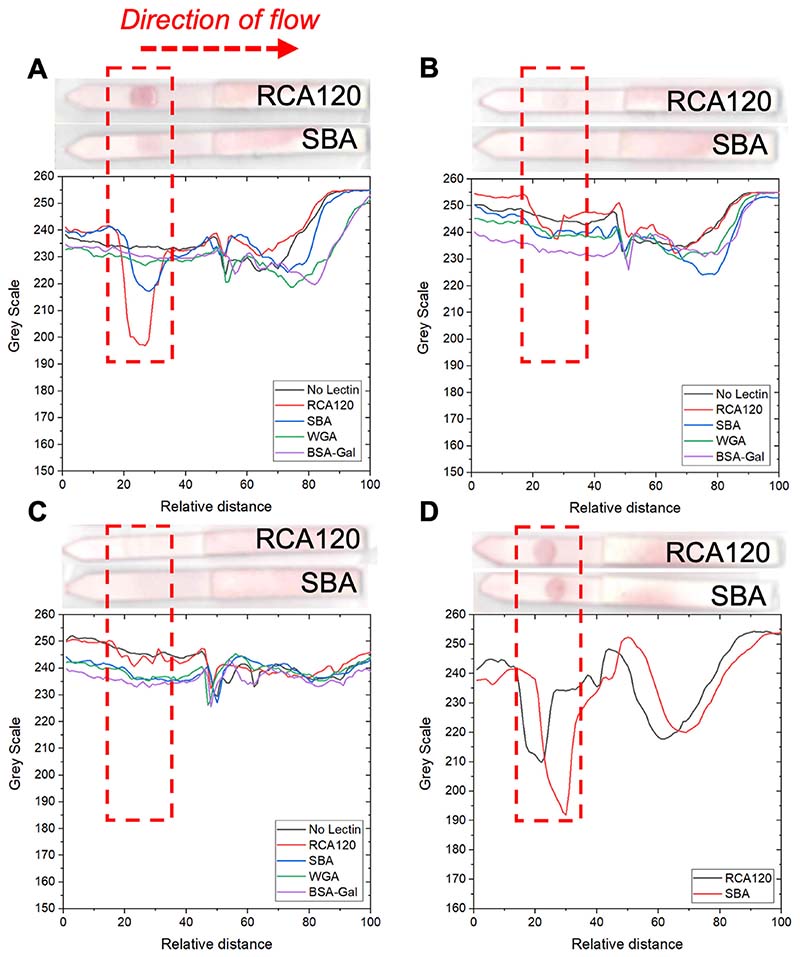
Analyzed flow-through data from RCA_120_ screen and inset are example dipstick photos. A) GalPHEA_40_@AuNP_16_; B) GalPHEA_72_@AuNP_16_; C) GalPHEA_110_@AuNP_16_; D) 2-deoxy-2-amino-GalPHEA_72_@AuNP_16_. Test lines were RCA_120_, SBA, or WGA at 5 mg mL^−1^, or BSA-Gal = Galα1-3Gal*β*1-4GlcNAc-BSA at 1 mg mL^−1^.

It was not possible to find a commercially available BSA-glycoconjugate with sufficient affinity for RCA_120_ to generate a test line. Therefore, an alternative approach, a “flow-through assay,”^[40,52,53]^ was used based on direct deposition of the target (RCA_120_ at 5 mg mL^−1^) onto the test line, followed by running the dipstick. Whilst unconventional, we have previously used this methodology in S1 spike protein detection.^[36]^ The dipsticks were run in the same manner as the SBA system and the results are summarized in Figure 5. In addition to RCA_120_ the following controls were tested; Wheat Germ Agglutinin (WGA) at 5 mg mL^−1^, a lectin with known affinity for *N*-acetyl-glucosamine,^[54]^ used to assess off-target binding; Galα1-3Gal*β*1-4GlcNAc-BSA at 1 mg mL^−1^, used to determine if a BSA glycoconjugate may serve as a viable test line in the future; and SBA at 5 mg mL^−1^. SBA was used as it has a known affinity to galactose residues,^[55]^ providing a challenge to design a flowthrough assay that only generates signal against RCA_120_. All images and analysis are available in the Figure S49 and Table S18, Supporting Information.

In contrast to what was observed with SBA, the averaged triplicate dipstick data for GalPHEA_40_@AuNP_16_ (Figure 5A) showed binding to RCA_120_ (and SBA) while the longer polymer PHEA_72_ (Figure 5B) showed very weak binding to RCA_120_ only (GalPHEA_110_@AuNP_16_ [Figure 5C] showed no clear binding to any lectins or controls). Notably 2-deoxy-2-amino-GalPHEA_72_@AuNP_16_ (Figure 5D) showed binding to both RCA_120_ and SBA but gave a stronger signal with SBA. This further shows that the optimal presentation of the glycan for each lectin is subtly different; but offers opportunities for tuning selectivity and affinity. Two additional polymers were therefore synthesized to fall between the 40–72 range of chain lengths already tested, to improve the assay, Table 2.

**Figure 6:**
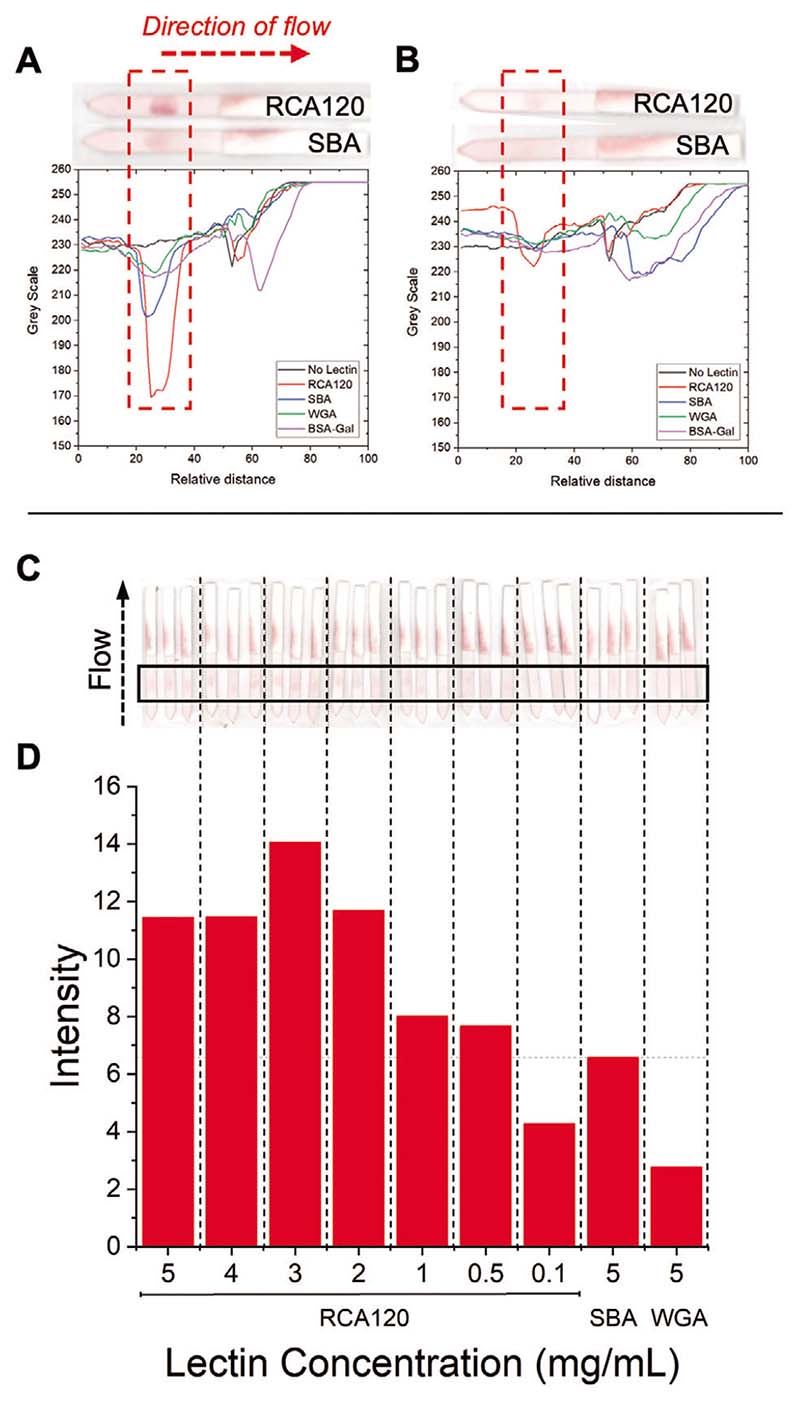
Flow-through dipstick assays against RCA_120_. A) Data from GalPHEA_50_ @AuNP_16_ and inset example dipsticks. B) Data from GalPHEA_58_@AuNP_16_ and inset example dipsticks. C) GalPHEA_58_@AuNP_16_ dipstick assays to determine limit of detection of RCA_120_. D) Analyzed limit of detection data of GalPHEA_58_@AuNP_16_ for RCA_120_. Test lines for (A) and (B) were RCA_120_, SBA, or WGA at 5 mg mL^−1^; or BSA-Gal = Galα1-3Gal*β*1-4GlcNAc-BSA at 1 mg mL^−1^.

The two additional polymers, PHEA_50_ and PHEA_58_ (Table 2) were functionalized with 1-deoxy-1-amino-galactose, immobilized onto 16 nm AuNPs, as described above, and fully characterized (Tables S17, S20, and S21 and Figures S47, S48, S52, and S57, Supporting Information). Subsequent evaluation in the same dipstick format found both bound to RCA_120_, generating positive test lines. Whilst both AuNPs were bound to the RCA_120_, the GalPHEA_58_@AuNP_16_ generated significantly weaker signal intensity against SBA and WGA controls (Figure 6B) compared to GalPHEA_50_@AuNP_16_ (Figure 6A). This confirmed that precision tuning the polymer chain length enables control of the overall signal generated and can provide additional discriminatory power to the assay. The identified optimum particle, 100% glycan-functionalized GalPHEA_58_@AuNP_16_, was next explored for its LoD in the dipstick assay. A serial dilution of RCA_120_ was prepared and deposited onto the strips (Figure 6C,D, and Table S19 and Figure S50, Supporting Information). The lowest concentration that could be detected, above the signal of a 5 mg mL^−1^ SBA control, was found to be 0.5 mg mL^−1^ (4.2 nmol mL^−1^).^[56]^

The dipsticks used above demonstrate the principle of lateral flow and flow-through glyco-assays for detecting lectins. However, a full device in a cassette format is required for a diagnostic which can be packaged, stored, distributed, and used easily. Therefore, cassettes designed to detect SBA (for which valid test and control lines were available) were assembled as proof of principle and prototype for a complete lateral flow glyco-assay for lectin detection.

**Figure 7:**
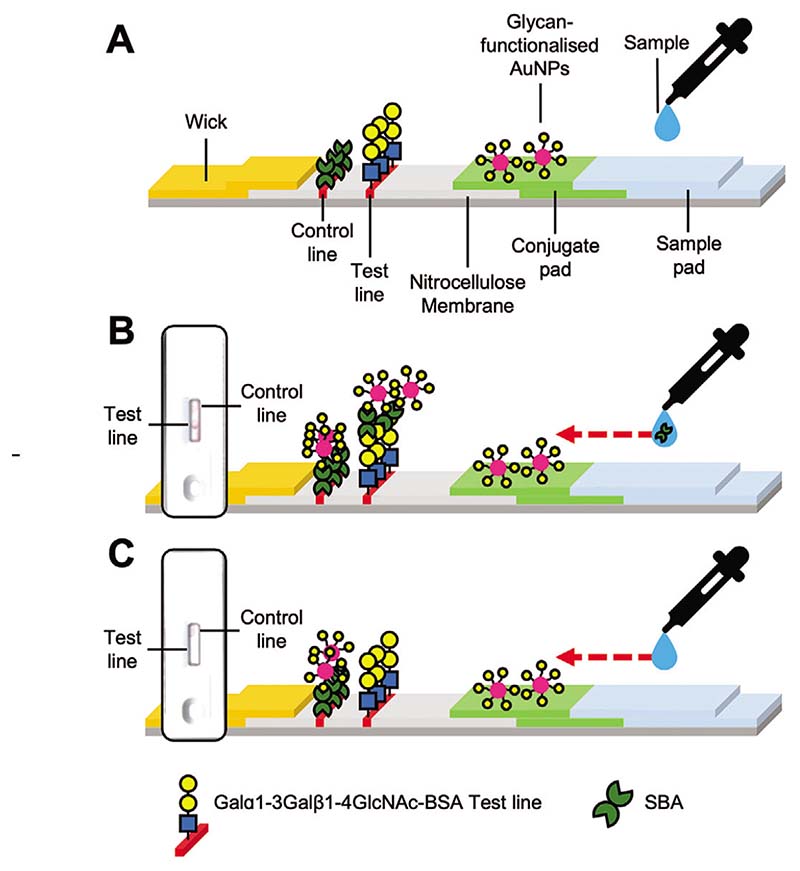
Schematic of complete cassette lateral flow for SBA binding and inlaid images of example cassettes. A) Labeled schematic of cassette. B) Lateral flow with SBA target in sample buffer. C) Lateral flow with no protein in buffer.

**Figure 8:**
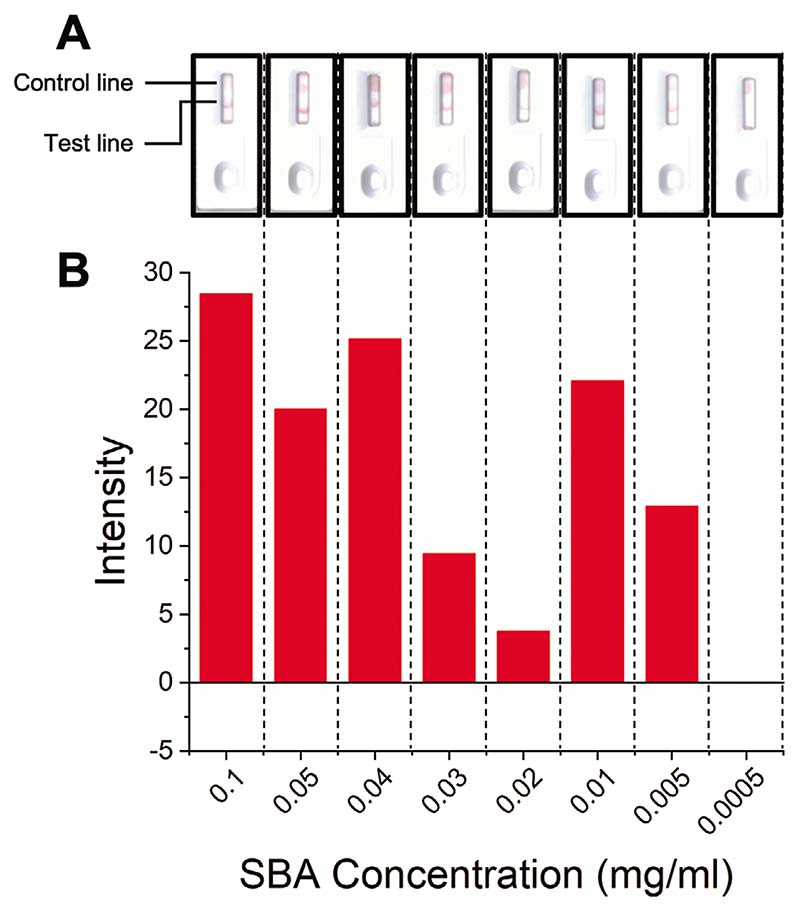
Lateral flow data from SBA cassette assays after 10 min to determine limit of detection. A) Example lateral flow cassette photographs for varying concentrations of SBA. B) Analyzed lateral flow intensity data for varying concentrations of SBA.

2-deoxy-2-amino-GalPHEA_72_@AuNP_16_ was selected as the optimal particle setup (from above), so particles were dried onto conjugate pads (from which they are released when the analyte solution is applied) and integrated into a complete cassette. A control line of 1 μL (5 mg mL^−1^) SBA was also added to the cassettes. A control line is essential in a functioning device to prove the device is running correctly (e.g., to distinguish between a negative result, and one where the particles did not flow) but was not used in the screening experiments above. Design schematics (Figure 7) and images of complete cassettes are shown in Figure 8 (and in full detail in the Figure S35 and Table S14, Supporting Information). Using this set up, concentrations of SBA as low as 5 μg mL^−1^ (0.042 nmol mL^−1^) could be detected in the buffer in 10 min (Figure 8). The drop in binding at 0.03 and 0.02 mg mL^−1^ indicates the difficulty in scanning the cassettes (when visually compared to the strips after removal from the devices, Table S14, Supporting Information) and variability between the hand-made devices. Notably all devices in the triplicates produced an observable signal and when averaged gave the values presented in Figure 8. In summary, Figure 8 validates the principle of the lateral flow glyco-assay, which can be adapted to other glycan-binding antigens, such as toxins or viruses. In each cassette a control line was also visible, confirming the devices ran correctly.

## 3 Conclusions

Here the emerging concept of lateral flow glyco-assays, as a tool for rapid diagnostics/sensing of glycan-binding analytes is validated. Polymeric ligands were used to install glycans onto AuNPs (which are the signal generating units) and provide both colloidal stability in solution while ensuring that the particles resuspend and flow in the LFDs. A library of polymer linker lengths (synthesized using RAFT polymerization), glycan density (by using polymers without glycans), and nanoparticle size was assembled and the impact of each feature on performance evaluated. A crucial observation was that the optimal polymer-coating required for the detection of SBA was not the same as required for RCA_120_. This is a unique advantage of employing the polymeric tethers, in that the final device’s performance and specificity can be tuned by macromolecular engineering, in addition to varying the exact glycan used. In general, too short polymers increase nonspecific binding, longer polymers reduced nonspecific binding but could reduce signal intensity also, while larger gold particles increase the signal of both nonspecific and specific binding. Therefore, tuning is essential to ensure that accurate and specific diagnostics can be developed.

The optimized glyconanoparticles were incorporated into “real” lateral flow cassettes, that is, a single device where a solution of analyte is applied to a well and run without any additional machine/user interfaces. Using this setup, SBA could be detected as low as 5 μg mL^−1^ (0.042 nmol mL^−1^) which is below the (molar) detection limits of commercial lateral flow pregnancy tests which use antibody-functionalized AuNPs and falls within the range of values (microgram to nanogram per milliliter) for antibody-based LFDs.^[57]^ Taken together, this work demonstrates the power of using glycans in easy to use, disposable, paper-based lateral flow glyco-assay diagnostics. By using glycans it is possible to probe function (e.g., is the antigen folded) and may provide opportunities for monitoring pathogenic state, rather than simply identifying if a pathogen is present.

## 4 Experimental Section

### Materials

All chemicals were used as supplied unless otherwise stated. *N*-Hydroxyethyl acrylamide (97%), 4,4’-azobis(4-cyanovaleric acid) (ACVA, 98%), 4-dimethylaminopyridine (>98%), mesitylene (reagent grade), triethylamine (>99%), sodium citrate tribasic dihydrate (>99%), gold(III) chloride trihydrate (99.9%), ammonium carbonate (reagent grade), potassium phosphate tri basic (≥98%, reagent grade), potassium hexafluorophosphate (99.5%), deuterium oxide (D_2_O, 99.9%), deuterated chloroform (CDCl_3_, 99.8%), diethyl ether (≥99.8%, ACS reagent grade), methanol (≥99.8%, ACS reagent grade), toluene (≥99.7%,), Tween-20 (molecular biology grade), HEPES, PVP40 (poly(vinyl pyrrolidone)_400_ [Average Mw ≈ 40 000]), sucrose (Bioultra grade), carbon disulfide (≥99.8%), acetone (≥99%), 1-dodecane thiol (>98%), n-pentylamine (99%), and pentafluorophenol (≥ 99%, reagent plus) were purchased from Sigma-Aldrich. Anhydrous trehalose was purchased from Alfa Aesar. DMF (>99%) and 2-bromo-2-methyl propionic acid (98%) were purchased from Acros Organics. Galactosamine HCl and 1-ethyl-3-(3-dimethylaminopropyl)carbodiimide hydrochloride (>98%), were purchased from Carbosynth. HPLC grade acetonitrile (≥99.8%), glucose (labreagent grade), hexane fraction from petrol (lab reagent grade), DCM (99% lab reagent grade), sodium hydrogen carbonate (≥99%), ethyl acetate (≥99.7%, analytical reagent grade), sodium chloride (≥99.5%), calcium chloride, 40–60 petroleum ether (lab reagent grade), hydrochloric acid (≈37%, analytical grade), glacial acetic acid (analytical grade), and magnesium sulfate (reagent grade) were purchased from Thermo Fisher Scientific.

Nitrocellulose Immunopore RP 90–150 s/4 cm 25mm was purchased from GE Healthcare. Lateral flow backing cards 60 mm by 301.58 mm (KN-PS1060.45 with KN211 adhesive) and lateral flow cassettes (KN-CT105) were purchased from Kenosha Tapes. Cellulose fiber wick material 20 cm by 30 cm by 0.825 mm (290 gsm and 180 mL min^−1^) (Surewick CFSP223000) was purchased from EMD Millipore. Glass fiber conjugate pads (GFCP103000) 10 mm by 300 mm and unfunctionalized BSA were purchased from Merck. Thick chromatography paper (for sample pads), Grade 237, Ahlstrom 20 cm by 20 cm was purchased from VWR International.

SBA, *Ricinus Communis* Agglutinin I (RCA_120_), *Ulex Europaeus* Agglutinin I, and WGA were purchased from Vector Laboratories. Galα1-3Gal*β*1-4GlcNAc-BSA (3 atom spacer, NGP0334) was purchased from Dextra Laboratories.

Ultrapure water used for buffers was MilliQ grade 18.2 mΩ resistance.

### Representative Polymerization of 2-Hydroxyethyl Acrylamide

PHEA40 as representative example. 2.0 g (17.37 mmol) of 2-hydroxyethyl acrylamide, 0.043 g (0.15 mmol) of ACVA, and 0.368 g (0.69 mmol) of PFP-DMP was added to 16 mL 1:1 toluene:methanol and degassed with nitrogen for 30 min. The reaction vessel was stirred and heated to 70 °C for 2 h. The solvent was removed under vacuum. The crude product was dissolved in the minimum amount of methanol. Diethyl ether cooled in liquid nitrogen was added to the methanol to form a precipitate. The mixture was centrifuged for 2 min at 13 krpm and the liquid decanted off. The solid was dissolved in methanol and removed under vacuum to give ayellowcrystalline solid.

PHEA40 — *δ*_H_ (300 MHz, D_2_O) 8.35–7.95 (21H, m, NH), 3.97–3.56 (78H, m, NHCH_2_), 3.56–3.03 (80H, m, CH_2_OH & SCH_2_), 2.41–1.90 (41H, m, CH_2_CHC(O) & C(CH_3_)_2_), 1.90 – 0.99 (108H, m, CH_2_CHC(O) & CH_2_CH_2_CH_2_CH_2_CH_2_CH_2_CH_2_CH_2_CH_2_CH_2_CH_3_), 0.83–0.72 (5H, m, CH_2_CH_3_); *δ*_F_ (300 MHz, D_2_O) – 152.0– –164.3 (5F, m, C_6_F_5_). FTIR (cm^−1^) – 3263 (OH, broad), 3088 & 2924 (C(O)NH and NH), 1638 & 1541 (C(O)NH) Yield – 73%

PHEA26 — *δ*_H_ (300 MHz, D_2_O) 8.38–7.88 (13H, m, NH), 3.96–3.54 (55H, m, NHCH_2_), 3.55–3.09 (78H, m, CH_2_OH & SCH_2_), 2.53–1.90 (31H, m, CH_2_CHC(O) & C(CH_3_)_2_), 1.90–1.01 (86H, m, CH_2_CHC(O) & CH_2_CH_2_CH_2_CH_2_CH_2_CH_2_CH_2_CH_2_CH_2_CH_2_CH_3_), 0.84–0.73 (5H, m, CH_2_CH_3_)

PHEA50 — *δ*_H_ (300 MHz, D_2_O) 8.31–7.97 (23H, m, NH), 3.99–3.55 (86H, m, NHCH_2_), 3.55–3.09 (100H, m, CH_2_OH & SCH_2_), 2.49–1.90 (46H, m, CH_2_CHC(O) & C(CH_3_)_2_), 1.90–0.98 (110H, m, CH_2_CHC(O) & CH_2_CH_2_CH_2_CH_2_CH_2_CH_2_CH_2_CH_2_CH_2_CH_2_CH_3_), 0.84–0.72 (5H, m, CH_2_CH_3_)

PHEA58 — *δ*_H_ (300 MHz, D_2_O) 8.36–7.98 (29H, m, NH), 4.00–3.55 (H, 108H, m, NHCH_2_), 3.55–3.15 (127H, m, CH_2_OH & SCH_2_), 2.36–1.88 (56H, m, CH_2_CHC(O) & C(CH_3_)_2_), 1.87–1.09 (128H, m, CH_2_CHC(O) & CH_2_CH_2_CH_2_CH_2_CH_2_CH_2_CH_2_CH_2_CH_2_CH_2_CH_3_), 0.83–0.72 (5H, m, CH_2_CH_3_)

PHEA72 — *δ*_H_ (300 MHz, D_2_O) 8.30–7.96 (34H, m, NH), 3.96–3.52 (126H, m, NHCH_2_), 3.52–3.07 (155H, m, CH_2_OH & SCH_2_), 2.36–1.88 (70H, m, CH_2_CHC(O) & C(CH_3_)_2_), 1.88–1.03 (148H, m, CH_2_CHC(O) & CH_2_CH_2_CH_2_CH_2_CH_2_CH_2_CH_2_CH_2_CH_2_CH_2_CH_3_), 0.82–0.70 (5H, m, CH_2_CH_3_)

PHEA110 — *δ*_H_ (300 MHz, D_2_O) 8.24–8.02 (28H, m, NH), 3.83–3.51 (239H, m, NHCH_2_), 3.51–3.08 (293H, m, CH_2_OH & SCH_2_), 2.40–1.90 (117H, m, CH_2_CHC(O) & C(CH_3_)_2_), 1.90–1.03 (273H, m, CH_2_CHC(O) & CH_2_CH_2_CH_2_CH_2_CH_2_CH_2_CH_2_CH_2_CH_2_CH_2_CH_3_), 0.86–0.73 (5H, m, CH_2_CH_3_)

### Representative Poly(N-Hydroxyethyl Acrylamide) (PHEA40) Glycan Functionalization

0.25 g (0.088 mmol) of poly(2-hydroxyethyl acrylamide)and 0.090 g (0.50 mmol) of galactosamine HCl were added to 25 mL of DMF containing 0.05 M M TEA. The reaction was stirred at 50 °C for 16 h. Solvent was removed under vacuum. The crude product was dissolved in the minimum amount of methanol at RTP before cooling in a liquid nitrogen bath. Diethyl ether cooled in liquid nitrogen was added to the methanol to form a precipitate. The mixture was centrifuged for 2 min at 13 krpm and the liquid decanted off. The solid was dissolved in methanol and removed under vacuum to give an orange/brown crystalline solid. *δ*_H_ (300 MHz, D_2_O) 8.03–7.86 (6H, m, NH), 4.96–4.87 (2H, anomeric protons), 4.13–3.51 (≈90H, m, NHCH_2_ & glycan protons), 3.51–3.09 (≈80H, m, CH_2_OH & SCH_2_ & glycan protons), 2.47–1.90 (≈50H, m, CH_2_CHC(O), C(CH_3_)_2_ & glycan protons), 1.90–1.42 (98H, m, CH_2_CHC(O) & CH_2_CH_2_CH_2_CH_2_CH_2_CH_2_CH_2_CH_2_CH_2_CH_3_), 0.93–0.72 (5H, m, CH_2_CH_3_). FTIR (cm^−1^) — 3267 (OH, broad), 3094 & 2926 (C(O)NH and NH), 1638 & 1545 (C(O)NH).

### Representative Poly(2-Hydroxyethyl Acrylamide) (PHEA40) PFP Removal with n-Pentylamine

0.4 g (0.14 mmol) of poly(2-hydroxyethyl acrylamide)and 0.05 mL (3.28 mmol) of n-pentylamine were added to 40 mL of DMF containing 0.05 M TEA. The reaction was stirred at 50 °C for 16 h. Solvent was removed under vacuum. The crude product was dissolved in the minimum amount ofmethanol at RTP before cooling in a liquid nitrogen bath. The crude product was dissolved in the minimum amount ofmethanol. Diethyl ether cooled in liquid nitrogen was added to the methanol to form a precipitate. The mixture was centrifuged for 2 min at 13 krpm and the liquid decanted off. The solid was dissolved in methanol and removed under vacuum to give a pale yellow crystalline solid. Removal of PFP was determined by ^19^F NMR.

### Gold Nanoparticle Polymer Coating Functionalization—16 nm

100 mg of glycopolymer was agitated overnight with 10 mL of 16 nm AuNPs ≈1 Abs at UV_max_. The solution was centrifuged at 13 krpm for 30 min and the pellet resuspended in 10 mL of water; the solution was centrifuged again at 13 krpm for 30 min and the pellet resuspended in 1 mL aliquots and centrifuged at 14.5 krpm for 10 min. The pellets were combined into a 1 mL solution with an absorbance at 520 nm of ≈10 Abs.

### Gold Nanoparticle Polymer Coating Functionalization—40 nm

100 mg of glycopolymer was agitated overnight with 10 mL of 40 nm AuNPs ≈1 Abs at UV_max_. The solution was centrifuged at 8 krpm for 30 min and the pellet resuspended in 10 mL of water, the solution was centrifuged again at 8 krpm for 30 min and the pellet resuspended in 1 mL aliquots and centrifuged at 8 krpm for 10 min. The pellets were combined into a 1 mL solution with an absorbance at UV_max_ of ≈10 Abs.

### Summary of Lateral Flow Strip Running Protocol and Analysis

Testlines were added and dried onto the dipsticks; in flow-through, the analyte was deposited in place ofa test line.

50 μL of running buffer (either with or without analyte) was agitated on a roller for 5 min. 45 μL of running buffer was added to a PCR tube; a dipstick was added to the tube, so the dipstick protruded from the top and the immobile phase (1 cm from nonwick end) was not below the solvent line. There was one test per tube and each test was run for 20 min before drying at room temperature for 5 min. All tests were run in triplicate.

The cassette running followed a similar procedure but used a total volume of 80 μL of running buffer and the tests run for 10 min before analysis ofthe triplicates.

A more detailed summary of dipstick and cassette manufacture, running, and analysis can be found in the Supporting Information.

### Statistical Analysis

All strips (dipsticks) and cassettes were run in triplicate.

All strips were attached to an acetate sheet and scanned using a Kyocera TASKalfa 5550ci printer to a pdf file that was converted to a jpeg; scans were taken within 1 h of strip drying. The jpeg was analyzed in ImageJ 1.51^[48]^ using the plot profile function to create a data set exported to Microsoft Excel for Mac. The data was exported to Origin 2019 64Bit and trimmed to remove pixel data not from the strip surface. The data was aligned and averaged (mean). The data was then reduced by number of groups to 100 data points (nitrocellulose and wick) and plotted as grey value (scale) versus relative distance along the 100 data points.

Signal to noise was determined for the strips as follows. Relative distance pixel 15–35 (area around the test line) was averaged (mean) to provide average noise around the test line for strips versus Galα1-3Gal*β*1-4GlcNAc-BSA (BSA-Gal) (1 mg mL^−1^) as a test line. The signal value was determined by selecting the lowest grey value between 15 and 35 relative distance pixels as a test line. Equation S1, Supporting Information, was then used to determine the signal to noise ratio.

Signal intensity was determined for the strips as follows. Relative distance pixel 15–35 (area around the test line), excluding pixels that contributed to the signal peak were averaged (mean). This average was subtracted from the lowest grey value between 15 and 35.

Signal intensity was determined for the cassettes as follows. Relative distance pixel 1–10 and 51–60 (area around the test line), excluding pixels that contributed to the signal peak were averaged (mean). This average was subtracted from the lowest grey value between 11 and 50 (test line region).

## Supplementary Material

Supporting Information is available from the Wiley Online Library or from the author.

Supporting Information

## Figures and Tables

**Table 1:** Polymers prepared for detecting SBA.

Polymer	[M]:[CTA]	*M*_n(theo)_ [g mol^−1^]^a)^	*M*_n(SEC)_ [g mol^−1^]^b)^	*M*_n(NMR)_ [g mol^−1^]^c)^	*Ð*_M_^b)^
PHEA_26_	10	1700	3600	4100	1.17
PHEA_40_	20	2800	5100	5000	1.19
PHEA_72_	40	5100	8900	8600	1.28
PHEA_110_	70	8600	13 000	14 000	1.27

a) ^Calculatedfromthefeed^ratio of monomer to chain transfer agent

b) Calculated against poly(methyl methacrylate) standards using 5 mM NH_4_BF_4_ in DMF as eluent

c) Determined from ^1^H NMR end-group analysis

**Table 2:** Additional polymers prepared for detecting RCA_120_.

Polymer	[M]:[CTA]	*M*_n(theo)_ [g mol^−1^]^a)^	*M*_n(SEC)_ [g mol^−1^]^b)^	*M*_n(NMR)_ [g mol^−1^]^c)^	*Ð*_M_^b)^
PHEA_50_	25	3400	6400	5500	1.27
PHEA_58_	30	4000	7200	6700	1.26

a) Calculated from the feed ratio of monomer to chain transfer agent

b) Calculated from SEC using poly (methyl methacrylate) standards

c) Determined from ^1^H NMR end-group analysis

## Data Availability

The data that support the findings of this study are available in the supplementary material of this article.
