# Supplementary material for: Lateral Flow Glyco-Assays for the Rapid and Low-Cost Detection of Lectins–Polymeric Linkers and Particle Engineering Are Essential for Selectivity and Performance
Source: Adv Healthc Mater. Author manuscript; Available in PMC 2022 Feb 17. (PMC7612396; doi:10.1002/adhm.202101784)
Supplement: Supplementary file 1 [file EMS140595-supplement-Supporting_Information.pdf]

Supplementary Information

Lateral Flow Glyco-Assays for the Rapid and Low-Cost Detection of Lectins - Polymeric Linkers and Particle Engineering are Essential for Selectivity and Performance

*Alexander N. Baker,[a] Asier R. Muguruza,[a,d] Sarah-Jane Richards,[a] Panagiotis G. Georgiou,[a] Stephen Goetz,[e] Marc Walker,[c] Simone Dedola,[e] Robert A. Field[f] and Matthew I. Gibson[a,b]**

[a] Department of Chemistry, University of Warwick, Gibbet Hill Road, CV4 7AL, Coventry, UK

[b] Warwick Medical School, University of Warwick, Gibbet Hill Road, CV4 7AL, Coventry, UK

[c] Department of Physics, University of Warwick, Gibbet Hill Road, CV4 7AL, Coventry, UK

[d] School of Chemistry, The University of Birmingham, Edgbaston, B15 2TT, Birmingham, UK

[e] Iceni Diagnostics Ltd, Norwich Research Park, Norwich, NR4 7GJ, UK

[f] Department of Chemistry and Manchester Institute of Biotechnology, University of Manchester, Manchester, M1 7DN, UK

**Corresponding Author*: m.i.gibson@warwick.ac.uk

Contents

Contents 2

Physical and Analytical Methods 5

NMR Spectroscopy 5

Mass Spectrometry 5

FTIR Spectroscopy 5

Size Exclusion Chromatography 5

X-ray Photoelectron Spectroscopy (XPS) 6

Dynamic Light Scattering 6

UV-vis Spectroscopy 7

Transmission Electron Microscopy 7

Image Collection of Lateral Flow Dipsticks and Devices 7

Materials 8

Synthetic Methods 10

Synthesis of 2-(dodecylthiocarbanothionylthio)-2-methyl propionic acid (DMP) 10

Synthesis of pentafluorophenyl-2-dodecylthiocarbonothioylthio)-2-methylpropanoate (PFP-DMP) 11

Synthesis of 1-deoxy-1-amino-galactose 12

Citrate-Stabilised 16 nm Gold Nanoparticle Synthesis[5] 12

Citrate-Stabilised 40 nm Gold Nanoparticle Synthesis 13

Lateral Flow Strip Production, Running and Analysis Protocols 14

Protocol for Manufacturing Lateral Flow Strips....14
Protocol for Test Line Addition to the Lateral Flow Strips....14
Protocol for Running Lateral Flow Test Without Target Analyte in Buffer....15
Protocol for Running Lateral Flow Test with Target Analyte in Buffer....15
Standard Protocol for Lateral Flow Strip Analysis....16
Lateral Flow Signal to Noise Analysis....17
Lateral Flow Signal Intensity Analysis....17
Lateral Flow Assay Buffer - 10× HEPES buffer (10% PVP_{400}) in 100 mL H_2O....17
Lateral Flow Complete Device Production, Running and Analysis Protocols....18
Protocol for Manufacturing Lateral Flow Complete Devices/Cassettes....18
Protocol for Conjugate Pad Production....19
10× Conjugate Pad Buffer....19
Protocol for Running Lateral Flow Test Without Target Analyte in Buffer....20
Protocol for Running Lateral Flow Test with Target Analyte in Buffer....20
Lateral Flow Signal Intensity Analysis....21
Additional Data and Figures....22
SBA-Targeting AuNP Data....27
DLS and UV-vis Data....27
Lateral Flow Strip Data....39
Lateral Flow Cassette Data....63
XPS Data....65

RCA120-Targeting AuNP Data 78

DLS and UV-vis Data 78

Flow-Through Strip Data 81

XPS Data 86

References 94

Physical and Analytical Methods

NMR Spectroscopy

^{1}H-NMR, ^{13}C-NMR and ^{19}F-NMR spectra were recorded at 300 MHz or 400 MHz on a Bruker DPX-300 or DPX-400 spectrometer respectively, with chloroform-*d* ($CDCl_3$) or deuterium oxide (D_2O) as the solvent. Chemical shifts of protons are reported as δ in parts per million (ppm) and are relative to either $CDCl_3$ (7.26) or D_2O (4.79).

Mass Spectrometry

Low resolution mass spectra (LRMS) were recorded on a Bruker Esquire 2000 spectrometer using electrospray ionisation (ESI). M/z values are reported in Daltons.

FTIR Spectroscopy

Fourier Transform-Infrared (FT-IR) spectroscopy measurements were carried out using an Agilent Cary 630 FT-IR spectrometer, in the range of 650 to 4000 cm^{-1}.

Size Exclusion Chromatography

Size exclusion chromatography (SEC) analysis was performed on an Agilent Infinity II MDS instrument equipped with differential refractive index (DRI), viscometry (VS), dual angle light scatter (LS) and variable wavelength UV detectors. The system was equipped with 2 x PLgel Mixed D columns (300 x 7.5 mm) and a PLgel 5 μm guard column. The mobile phase used was DMF (HPLC grade) containing 5 mM NH_4BF_4 at 50 °C at flow rate of 1.0 $mL.min^{-1}$. Poly(methyl methacrylate) (PMMA) standards (Agilent EasyVials) were used for calibration between 955,000 – 550 $g.mol^{-1}$. Analyte samples were filtered through a nylon membrane with 0.22 μm pore size before injection. Number average molecular weights (M_n), weight average molecular weights (M_w) and dispersities ($Đ_M = M_w/M_n$) were determined by conventional calibration and universal calibration using Agilent GPC/SEC software.

X-ray Photoelectron Spectroscopy (XPS)

The samples were attached to electrically-conductive carbon tape, mounted on to a sample bar and loaded into a Kratos Axis Ultra DLD spectrometer which possesses a base pressure below 1×10^{-10} mbar. XPS measurements were performed in the main analysis chamber, with the sample being illuminated using a monochromated Al Kα x-ray source. The measurements were conducted at room temperature and at a take-off angle of 90° with respect to the surface parallel. The core level spectra were recorded using a pass energy of 20 eV (resolution approx. 0.4 eV), from an analysis area of 300 μm x 700 μm. The spectrometer work function and binding energy scale of the spectrometer were calibrated using the Fermi edge and $3d_{5/2}$ peak recorded from a polycrystalline Ag sample prior to the commencement of the experiments. In order to prevent surface charging the surface was flooded with a beam of low energy electrons throughout the experiment and this necessitated recalibration of the binding energy scale. To achieve this, the C-C/C-H component of the C 1*s* spectrum was referenced to 285.0 eV. The data was analysed in the CasaXPS package, using Shirley backgrounds and mixed Gaussian-Lorentzian (Voigt) lineshapes. For compositional analysis, the analyser transmission function has been determined using clean metallic foils to determine the detection efficiency across the full binding energy range.

Dynamic Light Scattering

Hydrodynamic diameters (D_h) and size distributions of particles were determined by dynamic light scattering (DLS) using a Malvern Zetasizer Nano ZS with a 4 mW He-Ne 633 nm laser module operating at 25 °C. Measurements were carried out at an angle of 173° (back scattering), and results were analysed using Malvern DTS 7.03 software. All determinations were repeated 5 times with at least 10 measurements recorded for each run. D_h values were calculated using the Stokes-Einstein equation where particles are assumed to be spherical.

UV-vis Spectroscopy

Absorbance measurements were recorded on an Agilent Cary 60 UV-Vis Spectrophotometer and on a BioTek Epoch microplate reader.

Transmission Electron Microscopy

Dry-state stained TEM imaging was performed on a JEOL JEM-2100Plus microscope operating at an acceleration voltage of 200 kV. All dry-state samples were diluted with deionized water and then deposited onto formvar-coated copper grids.

Image Collection of Lateral Flow Dipsticks and Devices

All devices were scanned using a Kyocera TASKalfa 5550ci printer to a pdf file that was converted to a jpeg. The jpeg was analysed in ImageJ 1.51.[1] None of the images in this ESI have been image adjusted i.e. no changes/enhancements have been made from the original scan images.

Materials

All chemicals were used as supplied unless otherwise stated. *N*-Hydroxyethyl acrylamide (97%), 4,4'-azobis(4-cyanovaleric acid) (ACVA, 98%), 4-dimethylaminopyridine (DMAP, > 98%), mesitylene (reagent grade), triethylamine (> 99%), sodium citrate tribasic dihydrate (> 99 %), gold(III) chloride trihydrate (99.9%), ammonium carbonate (reagent grade), potassium phosphate tri basic (≥ 98%, reagent grade), potassium hexafluorophosphate (99.5%), deuterium oxide (D_2O, 99.9%), deuterated chloroform ($CDCl_3$, 99.8%), diethyl ether (≥ 99.8%, ACS reagent grade), methanol (≥ 99.8%, ACS reagent grade), toluene (≥ 99.7%,), Tween-20 (molecular biology grade), HEPES, PVP40 (poly(vinyl pyrrolidone)$_{400}$ (Average Mw ~40,000)), sucrose (Bioultra grade), carbon disulphide (≥ 99.8%), acetone (≥ 99%), 1-dodecane thiol (≥ 98%), n-pentylamine (99%) and pentafluorophenol (≥ 99%, reagent plus) were purchased from Sigma-Aldrich. Anhydrous trehalose was purchased from Alfa Aesar. DMF (> 99%), 2-bromo-2-methyl propionic acid (98%) were purchased from Acros Organics. Galactosamine HCl and 1-Ethyl-3-(3-dimethylaminopropyl)carbodiimide hydrochloride (EDCI, > 98%),was purchased from Carbosynth. HPLC grade acetonitrile (≥ 99.8%), glucose (lab-reagent grade), hexane fraction from petrol (lab reagent grade), DCM (99% lab reagent grade), sodium hydrogen carbonate (≥ 99%), ethyl acetate (≥ 99.7%, analytical reagent grade), sodium chloride (≥ 99.5%), calcium chloride, 40-60 petroleum ether (lab reagent grade), hydrochloric acid (~37%, analytical grade), glacial acetic acid (analytical grade) and magnesium sulphate (reagent grade) were purchased from Thermo Fisher Scientific.

Nitrocellulose Immunopore RP 90-150 s/4cm 25mm was purchased from GE Healthcare. Lateral flow backing cards 60mm by 301.58mm (KN-PS1060.45 with KN211 adhesive) and lateral flow cassettes (KN-CT105) were purchased from Kenosha Tapes. Cellulose fibre wick material 20 cm by 30 cm by 0.825 mm (290 gsm and 180 mL/min) (Surewick CFSP223000) was purchased from EMD Millipore. Glass fibre conjugate pads (GFCP103000) 10 mm by 300

mm and unfunctionalised BSA were purchased from Merck. Thick Chromatography Paper (for sample pads), Grade 237, Ahlstrom 20 cm by 20 cm was purchased from VWR International.

Soybean agglutinin, *Ricinus Communis* Agglutinin I (RCA_{120}), *Ulex Europaeus* Agglutinin I and wheat germ agglutinin (WGA) were purchased from Vector Laboratories. Galα1-3Galβ1-4GlcNAc-BSA (3 atom spacer, NGP0334) was purchased from Dextra Laboratories.

Ultra-pure water used for buffers was MilliQ grade 18.2 mΩ resistance.

Synthetic Methods

Synthesis of 2-(dodecylthiocarbanothionylthio)-2-methyl propionic acid (DMP)

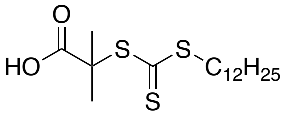

This was synthesised, according to a previously published procedure.[2] 2.00 g (9.88 mmmol) of 1-dodecane thiol was added dropwise to stirring 2.10 g (9.89 mmol) of K_3PO_4 in 30mL of acetone at RTP, the mixture was left to stir for 25 minutes to form a white suspension. 2.05 g (26.93 mmol) of carbon disulphide was then added and left for 10 minutes, a yellow solution formed. 1.5 g (8.98 mmol) of 2-bromo-2-methyl-propionic acid was then added and the solution left to stir for 16 hours. The solvent was removed under vacuum. The crude product was dissolved in 100 mL of 1M HCl and extracted with DCM (2×100 mL). The organic layer was washed with 200 mL water and 200 mL brine. The organic layer was dried with $MgSO_4$ and filtered under gravity. The solvent was then removed from the filtrate under vacuum. The crude product was purified using a silica column (40-60 PET:DCM:glacial acetic acid 75:24:1) and recrystalised in n-hexane to give a yellow solid (58%). δ_H (300 MHz, $CDCl_3$) 3.28 (2H, t, *J* 7.5, SCH_2CH_2), 1.80 - 1.45 (8H, m, $C(CH_3)_2$ and SCH_2CH_2), 1.45 - 1.2 (18H, m, $(CH_2)_9CH_3$), 0.87 (3H, t, *J* 6.0, CH_3). δ_C (400 MHz, $CDCl_3$) 221.0 (1C, SC(S)S), 178.3 (1C, C(O)), 55.7 (1C, $C(CH_3)_2$), 37.7 (1C, SCH_2), 32.1 - 28.0 (9C, $SCH_2(CH_2)_9$), 25.4 (2C, $C(CH_3)_2$), 22.8 (1C, CH_2CH_3), 14.3 (1C, CH_2CH_3). m/z calculated as 364.16; found for ESI $[M+H]^+$ 365.3 and $[M+Na]^+$ 387.3. FTIR (cm^{-1}) – 2956, 2916.6 & 2850 (methyl and methylene), 1702 (ester C=O), 1459, 1437 & 1413 (methyl and methylene), 1280 ($C(CH_3)_2$), 1064 (S-C(S)-S).

Synthesis of pentafluorophenyl-2-dodecylthiocarbonothioylthio)-2-methylpropanoate (PFP-DMP)

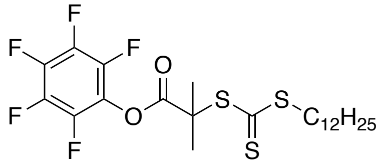

This was synthesised, according to a previously published procedure.[2] 4.06 g (11.13 mmol) of DMP, 3.65 g (19.04 mmol) of EDC and 2.30 g (18.82 mmol) of DMAP were dissolved in 160 mL of DCM and degassed for 30 minutes. 7.28 g (39.55 mmol) of pentaflurophenol was added in 20 mL of DCM and the mixture stirred for 18 hours at RTP. The organic layer was washed with 3 M HCl (200mL), 1 M $NaHCO_3$ (200 mL) and 0.5 M NaCl (200 mL). The organic layer was dried with $MgSO_4$ and filtered under gravity. The solvent was then removed from the filtrate under vacuum. The crude product was recrystallised in ethyl acetate (or hexane) overnight at -8°C and dried to give yellow crystals (90.9%). δ_H (300 MHz, $CDCl_3$) 3.31 (2H, t, *J* 7.5, SCH_2CH_2), 1.86 (6H, s, $C(CH_3)_2$), 1.69 (2H, qn, *J* 7.5, SCH_2), 1.48 - 1.16 (18H, m, $CH_2CH_2CH_2CH_2CH_2CH_2CH_2CH_2CH_2CH_3$), 0.94 - 0.82 (3H, m, CH_3). δ_C (300 MHz, $CDCl_3$) 220.1 (1C, SC(S)S), 169.7 (1C, C(O)), 143.1 (2C, meta C), 139.8 (1C, ipso C), 139.6 (1C, para C), 136.3 (2C, Ortho C), 55.5 (1C, $C(CH_3)_2$), 37.3 (1C, SCH_2), 32.0 - 22.8 (10C, $SCH_2(CH_2)_{10}$), 25.4 (2C, $C(CH_3)_2$), 14.1 (1C, CH_2CH_3). δ_F (300 MHz, $CDCl_3$) -151.44 - -151.61 (2F, m, $OCC_2H_2C_2H_2CH$), -148.50 (1F, t, *J* 21.5, $OCC_2H_2C_2H_2CH$), -162.23 - -162.47 (2F, m, $OCC_2H_2C_2H_2CH$). m/z calculated as 530.14; found for ESI $[M+Na]^+$ 553.3 and $[M+CH_3CN+Na]^+$ 593.5. FTIR (cm^{-1}) – 2956, 2917 & 2850 (methyl and methylene), 1702 (ester C=O), 1519 (aromatic C=C or C-F), 1460, 1437 & 1413 (methyl and methylene), 1280 ($C(CH_3)_2$), 1068 (S-C(S)-S).

Synthesis of 1-deoxy-1-amino-galactose

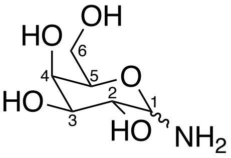

This was synthesised, according to previously published procedures.[3,4] 0.36 g (2 mmol) of galactose and 0.158 g (2 mmol) ammonium hydrogen carbonate were added to a 10 mL solution of 16 mol.dm^{-3} ammonia solution. The mixture was heated for 16 hours at 42 °C. The liquid was removed under vacuum and resuspended in 5mL of water. The solution was lyophilised to give a cream or off-white crystalline solid, that did not smell of ammonia. (94.6%) (α:β 0.71:0.38). δ_H (300 MHz, $CDCl_3$) 4.24 (0.38H, d, *J* 8.81, C-1H$^{\alpha}$), 4.03 (0.71H, d, *J* 8.75, C-1H$^{\beta}$), 3.93 (1H, d, *J* 3.00, C-3H or C-4H), 3.90-3.45 (8H, m, C-3H or C-4H, C-6H$_2$, C-5H and hydroxyls), 3.39 (1H, t, *J* 9.0, C-2H). δ_C (300 MHz, $CDCl_3$) 87.6 (C-1H$^{\alpha}$), 85.5 (C-1H$^{\beta}$), 76.0 (C-5), 73.4 (C-3 or C-4), 72.0 (C-2), 68.96 (C-3 or C-4), 61.1 (C-6). m/z calculated as 179.171; found for ESI [2M+Na]$^+$ 381.3 and [M-H$^+$]$^-$ 178.1. FTIR (cm^{-1}) – 3650 - 2500 (hydroxyl), 2924 & 2878 (alkane), 1646 & 1584 (amine), 1465 & 1420 (hydroxyl).

Citrate-Stabilised 16 nm Gold Nanoparticle Synthesis[5]

To 500 mL of water was added 0.163 g (0.414 mmol) of gold(III) chloride trihydrate, the mixture was heated to reflux and 14.6 mL of water containing 0.429 g (1.46 mmol) of sodium citrate tribasic dihydrate was added. The reaction was allowed to reflux for 30 minutes before cooling to room temperature over 3 hours. The solution was centrifuged at 13 krpm for 30 minutes and the pellet resuspended in 40 mL of water to give an absorbance at 520 nm of ~1Abs.

Citrate-Stabilised 40 nm Gold Nanoparticle Synthesis

40 nm gold nanoparticles were synthesised by a modified step growth method developed by Bastús *et al.*.[6] A solution of 2.2 mM sodium citrate in Milli-Q water (150 mL) was heated under reflux for 15 min under vigorous stirring. After boiling had commenced, 1 mL of $HAuCl_4$ (25 mM) was injected. The colour of the solution changed from yellow to bluish gray and then to soft pink in 10 min, 1 mL was taken for DLS and UV/Vis analysis. Immediately after the synthesis of the Au seeds and in the same reaction vessel, the reaction was cooled until the temperature of the solution reached 90 °C. Then, 1 mL of a $HAuCl_4$ solution (25 mM) was injected. After 20 min, the reaction was finished. This process was repeated twice. After that, the sample was diluted by adding 85 mL of MilliQ water and 3.1 mL of 60 mM sodium citrate. This solution was then used as a seed solution, and three further portions of 1.6 mL of 25 mM $HAuCl_4$ were added with 20 min between each addition. Following completion of this step 1 mL was taken for DLS and UV/Vis analysis. The sample was diluted by adding 135 mL of MilliQ water and 4.9 mL of 60 mM sodium citrate. This solution was then used as a seed solution, and the process was repeated with three further additions of 2.5 mL of 25 mM $HAuCl_4$, this solution was analysed by DLS and UV/Vis and target size of 40 nm was reached, so the solution was allowed to cool.

Lateral Flow Strip Production, Running and Analysis Protocols

The procedure to produce flow-through and lateral flow devices was identical, apart from the deposition of the analyte directly to the nitrocellulose (flow-through), versus application of tests lines to the nitrocellulose (lateral flow).

Protocol for Manufacturing Lateral Flow Strips

Backing cards were cut to size by removal of 20 mm using a guillotine. Nitrocellulose was added to the backing card by attaching the plastic backing of the nitrocellulose to the self-adhesive on the card. The wick material was then added to the backing card so it overlaps with the nitrocellulose by ~5 mm. The lateral flow strips were cut to size of width 2-3 mm.

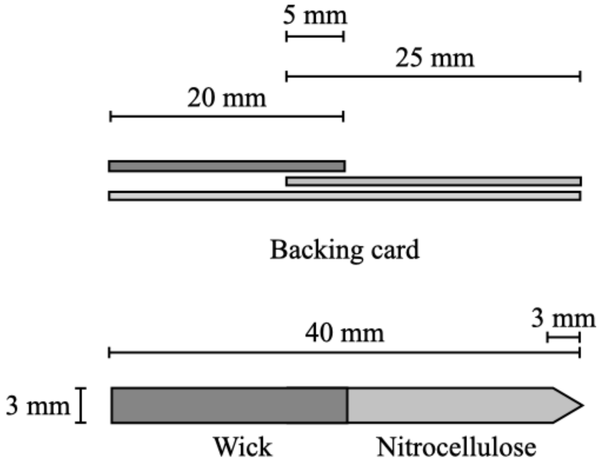

Figure S1. – Lateral flow strip dimensions

Protocol for Test Line Addition to the Lateral Flow Strips

1 μL of the test line solution was added to the test strip using a micropipette fitted with 10 μL tip, the test line was spotted ~1 cm from the non-wick end of the strip. The strips were dried at 37 °C in an oven for 30 minutes. The tests strips were allowed to cool to room temperature before testing.

Protocol for Running Lateral Flow Test Without Target Analyte in Buffer

The running buffer of total volume 50 µL was made as follows; 5 µL AuNPs (OD10), 5 µL lateral flow assay buffer – 10 × HEPES buffer, 40 µL water. The running solution was then agitated on a roller for 5 minutes. 45 µL of this solution was added to a 0.2 mL PCR tube, standing vertically.

A small "v" (~3 mm) was cut into the test strips at the non-wick end and the strips added to the PCR tubes, so they protrude from the top and the immobile phase (1 cm from non-wick end) is not below the solvent line. There was one test per tube. All tests were run in triplicate.

The tests were run for 20 minutes before removal from the tubes. The test strips were allowed to dry at room temperature for ~5 minutes. The test strips were mounted test-face down onto a clear and colourless piece of acetate sheeting.

The *Protocol for Running Lateral Flow Test Without Target Analyte in Buffer* was used for the flow-through assays as the target analyte is deposited on the nitrocellulose as a "test line" i.e. the analyte is not in the running buffer.

Protocol for Running Lateral Flow Test with Target Analyte in Buffer

The running buffer of total volume 50 µL was made as follows; 5 µL AuNPs (OD10), 5 µL lateral flow assay buffer – 10 × HEPES buffer, 40 µL of water - x µL, where x is the volume of target analyte added to make the required concentration of the lectin. The running solution was then agitated on a roller for 5 minutes. 45 µL of this solution was added to a 0.2 mL PCR tube, standing vertically.

A small "v" (~3 mm) was cut into the test strips at the non-wick end and the strips added to the PCR tubes, so they protrude from the top and the immobile phase (1 cm from non-wick end) is not below the solvent line. There was one test per tube. All tests were run in triplicate.

The tests were run for 20 minutes before removal from the tubes. The test strips were allowed to dry at room temperature for ~5 minutes. The test strips were mounted test-face down onto a clear and colourless piece of acetate sheeting.

Standard Protocol for Lateral Flow Strip Analysis

The acetate sheets were scanned using a Kyocera TASKalfa 5550ci printer to a pdf file that was converted to a jpeg, scans were taken within 1 hour of strip drying. The jpeg was analysed in ImageJ 1.51[1] using the plot profile function to create a data set exported to Microsoft Excel for Mac. The data was exported to Origin 2019 64Bit and trimmed to remove pixel data not from the strip surface. The data was aligned and averaged (mean). The data was then reduced by number of groups to 100 data points (nitrocellulose and wick) and plotted as Grey value (scale) vs Relative distance along the 100 data points.

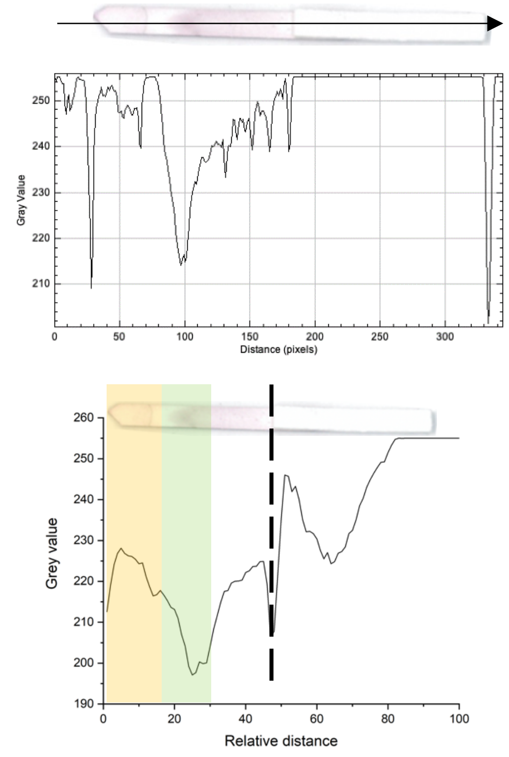

Figure S2. – Representative dipstick (Top), raw grey value plot (Middle) and processed grey value plot (Bottom)

Lateral Flow Signal to Noise Analysis

Relative distance pixel 15 to 35 (area around the test line) was averaged (mean) to provide average noise around the test line for strips vs. Galα1-3Galβ1-4GlcNAc-BSA (BSA-Gal) (1 mg/mL) as a test line. The signal value was determined by selecting the lowest grey value between 15 to 35 relative distance pixels as a test line. Equation 1 was then used to determine the signal to noise ratio.

$$Signal\ to\ Noise = \frac{255 - Signal}{255 - Noise}$$

Equation 1. – Equation for determining signal to noise ratio

NB: 255 is the grey value for the blank nitrocellulose surface.

Lateral Flow Signal Intensity Analysis

Relative distance pixel 15 to 35 (area around the test line), excluding pixels that contributed to the signal peak were averaged (mean). This average was subtracted from the lowest grey value between 15 to 35.

Lateral Flow Assay Buffer - 10× HEPES buffer (10% PVP_{400}) in 100 mL H_2O

2.38 g (100 mmol.dm^{-3}) of HEPES, 8.77 g (1.50 mol.dm^{-3}) of NaCl, 0.011 g (1.0 mmol.dm^{-3}) of $CaCl_2$, 0.8 g (0.8% w/v., 123 mmol.dm^{-3}) of NaN_3, 0.5 g (0.5% w/v., 4.07 mmol.dm^{-3}) of Tween-20 and 10 g (10% w/v.) of poly(vinyl pyrrolidone)$_{400}$ (PVP_{400}, Average Mw ~40,000) were dissolved in 100 mL of water. The buffer was not pH adjusted.

Lateral Flow Complete Device Production, Running and Analysis Protocols

Protocol for Manufacturing Lateral Flow Complete Devices/Cassettes

Nitrocellulose was added to the backing card by attaching the plastic backing of the nitrocellulose to the self-adhesive on the card. The wick material was then added to the backing card so it overlaps with the nitrocellulose by ~5 mm. The strips were then cut to size of width ~3 mm so they sit in the cassettes without the need for excess force to fit. Tests lines were then added before addition of the conjugate pad. 1 µL of the test line solution was added to the nitrocellulose strip using a micropipette fitted with 10 µL tip, the test line was spotted ~1 cm from the non-wick end of the nitrocellulose surface. A control line was added ~1.5 cm from non-wick end of the nitrocellulose surface. The control for galactosamine systems was 1 µL (5 $mg.mL^{-1}$) SBA. The strips were dried at 37 °C in an oven for 30 minutes. The tests strips were allowed to cool to room temperature before addition of the conjugate pad. The conjugate pad was added to the backing card so it overlaps with the nitrocellulose by ~3.5 mm. The sample pad, was cut to size (20 mm by 6 mm) and added to the backing card, overlapping with the conjugate pad by ~6.5 mm and straddling the backing card evenly. The completed strip was then added to the cassettes and sealed.

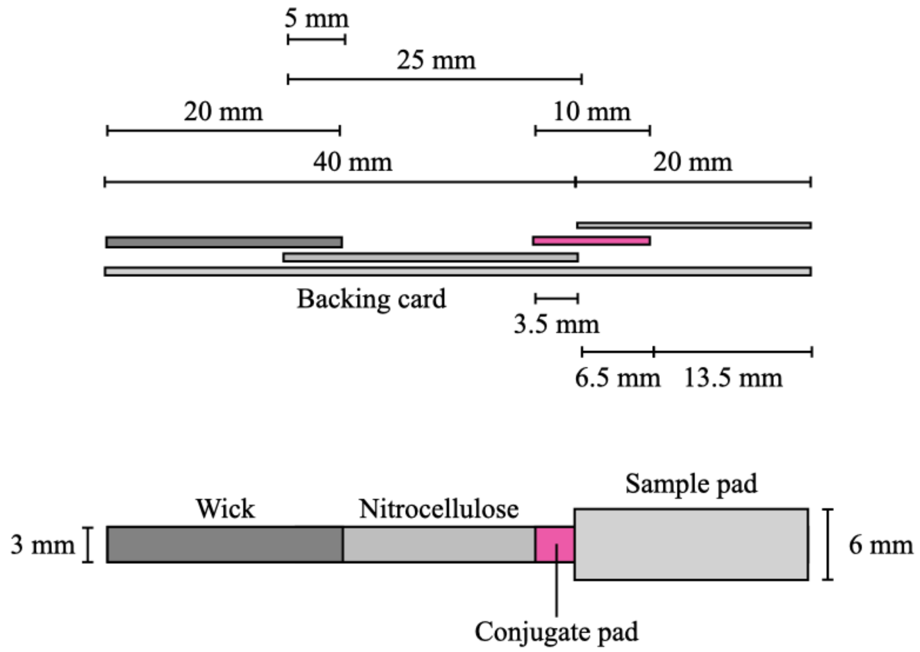

Figure S3. – Lateral flow complete strip dimensions

Protocol for Conjugate Pad Production

Strips of the conjugate pad material were agitated for 30 minutes in a solution of 0.1% Tween-20 (blocking solution). The strips were then patted dry and baked overnight at 37 °C in an oven. The conjugate pads were cut to size (3 mm width) and placed individually into the wells of a 384-well microplate. 20 µL 1× conjugate pad buffer solution containing OD1 AuNPs was added to the top of each conjugate pad in the wells. The pads were dried for 3 hours at 37 °C in an oven before curing overnight in an airtight box containing desiccant. The completed pads were always stored in an airtight box containing desiccant.

10× Conjugate Pad Buffer

10% w/v. of poly(vinyl pyrrolidone)$_{400}$ (Average Mw ~40,000 g.mol^{-1}), 50% w/v. trehalose, 10% w/v. sucrose and 0.1% w/v. Tween-20 were added to distilled water and allowed to dissolve.

Protocol for Running Lateral Flow Test Without Target Analyte in Buffer

8 µL 10× HEPES buffer (20% PVP_{400}) was added to 72 µL distilled water. 80 µL was added to the sample pad and allowed to absorb. The test was run for 10 minutes before scanning the cassettes using a Kyocera TASKalfa 5550ci printer, the images were exported to a pdf file that was converted to a jpeg. Within ~1 hour the strips were removed from the cassettes and added to acetate sheets. These were scanned using a Kyocera TASKalfa 5550ci printer to a pdf file that was converted to a jpeg, acetate scans were taken within 1 hour of strip drying. The jpegs were analysed in Image J 1.51 using the plot profile function to create a data set exported to Microsoft Excel for Mac. The data was exported to Origin 2019 64Bit and trimmed to remove pixel data not from the strip surface. The data was aligned and averaged (mean). The data was then reduced by number of groups to 100 data points (just the nitrocellulose surface) and plotted as Grey value (scale) vs Relative distance along the 100 data points.

Protocol for Running Lateral Flow Test with Target Analyte in Buffer

8 µL 10× HEPES buffer (20% PVP_{400}) was added to 72 µL of water - x µL, where x is the volume of target analyte added to make the required concentration of the lectin. 80 µL was added to the sample pad and allowed to absorb. The test was run for 10 minutes before scanning the cassettes using a Kyocera TASKalfa 5550ci printer, the images were exported to a pdf file that was converted to a jpeg. Within ~1 hour the strips were removed from the cassettes and added to acetate sheets. These were scanned using a Kyocera TASKalfa 5550ci printer to a pdf file that was converted to a jpeg, acetate scans were taken within 1 hour of strip drying. The jpegs were analysed in Image J 1.51 using the plot profile function to create a data set exported to Microsoft Excel for Mac. The data was exported to Origin 2019 64Bit and trimmed to remove pixel data not from the strip surface. The data was aligned and averaged (mean). The data was then reduced by number of groups to 100 data points (just the nitrocellulose surface) and plotted as Grey value (scale) vs Relative distance along the 100 data points.

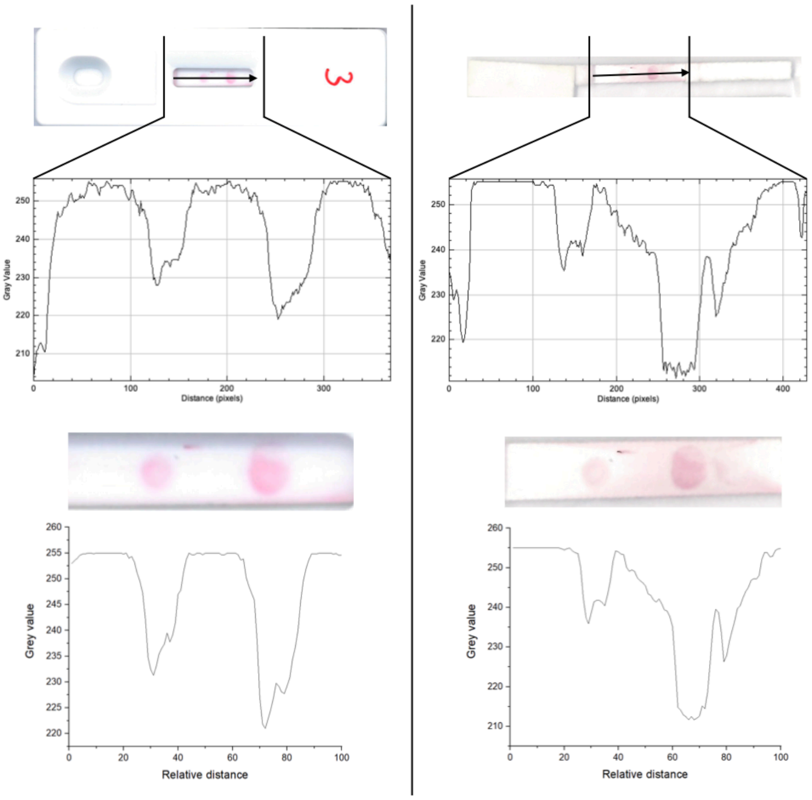

Figure S4. – Representative cassette (Top left) and strip (Top right), raw grey value plot (Middle) and processed grey value plot (Bottom)

Lateral Flow Signal Intensity Analysis

Relative distance pixel 1 to 10 and 51 to 60 (area around the test line), excluding pixels that contributed to the signal peak were averaged (mean). This average was subtracted from the lowest grey value between 11 to 50 (test line region).

Additional Data and Figures

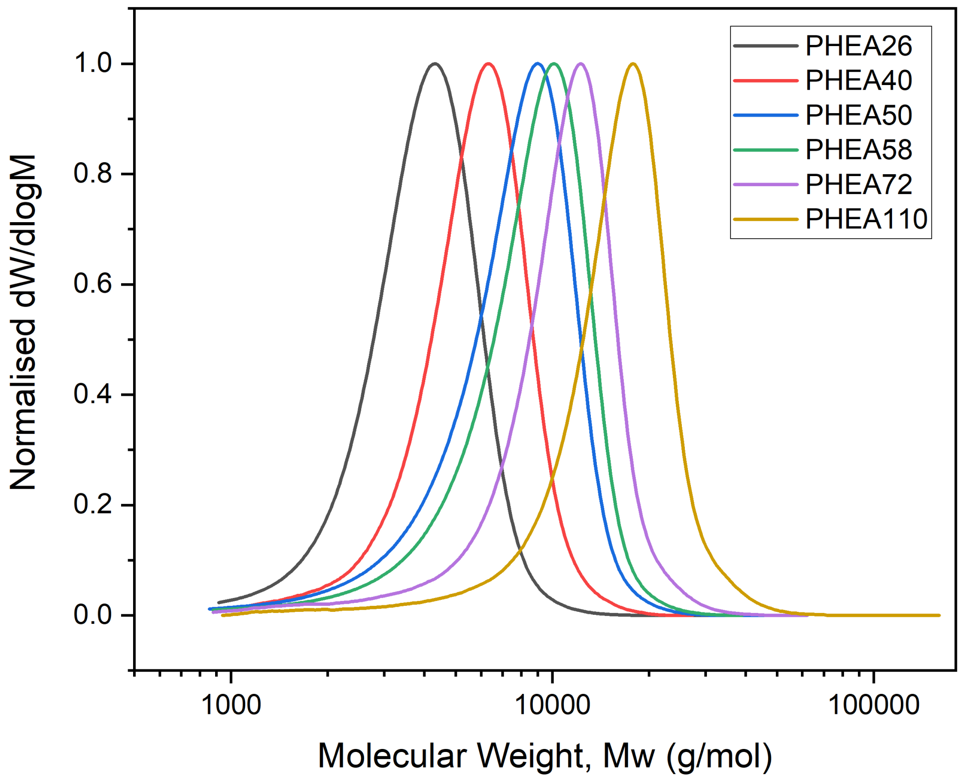

Figure S5. – Normalised size exclusion chromatography RI molecular weight distributions of telechelic PHEA obtained in DMF versus PMMA standards.

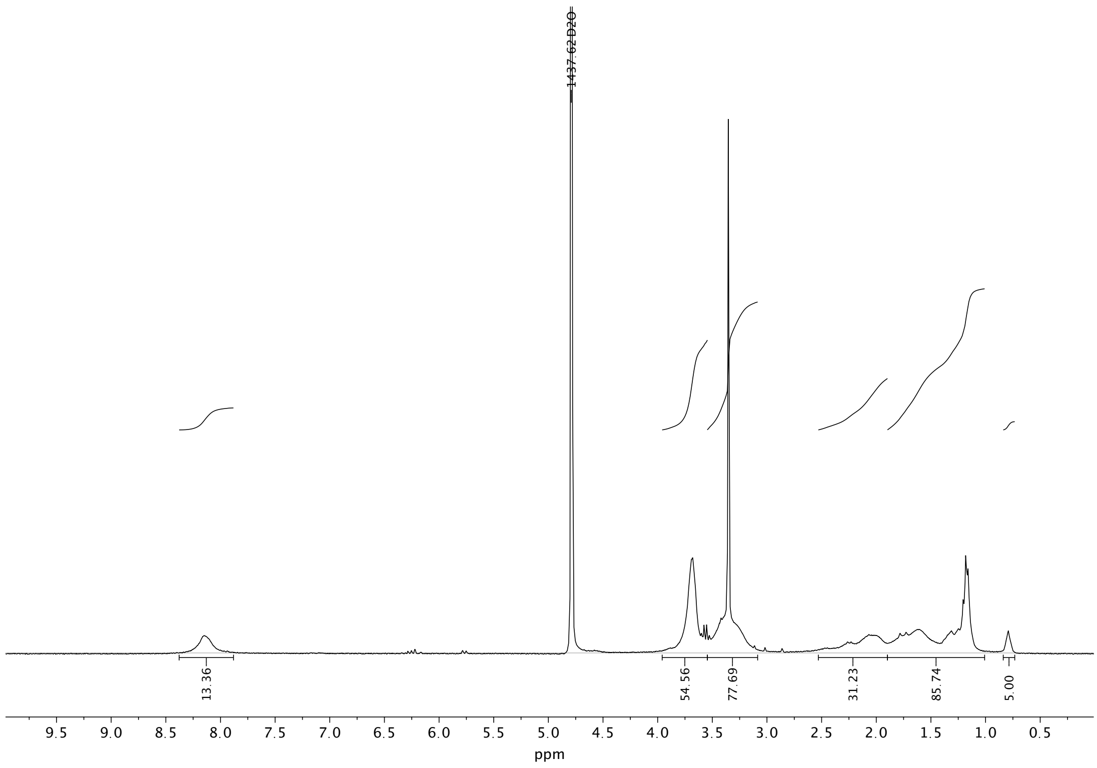

Figure S6. – ^{1}H NMR spectrum of PHEA26

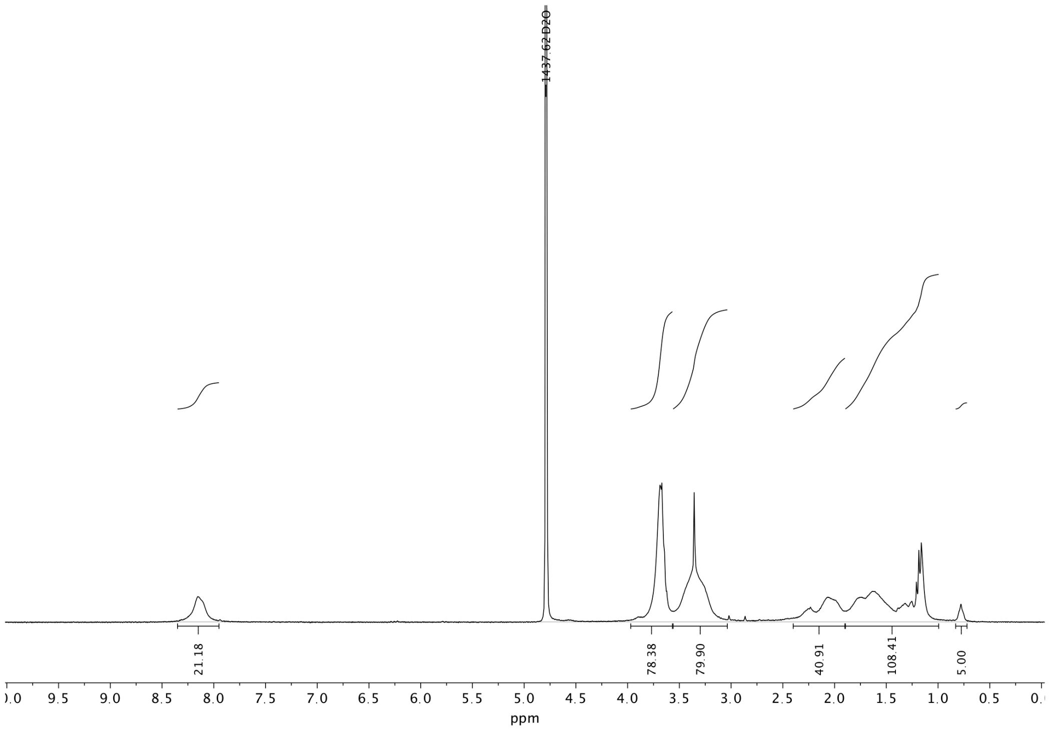

Figure S7. – ^{1}H NMR spectrum of PHEA40

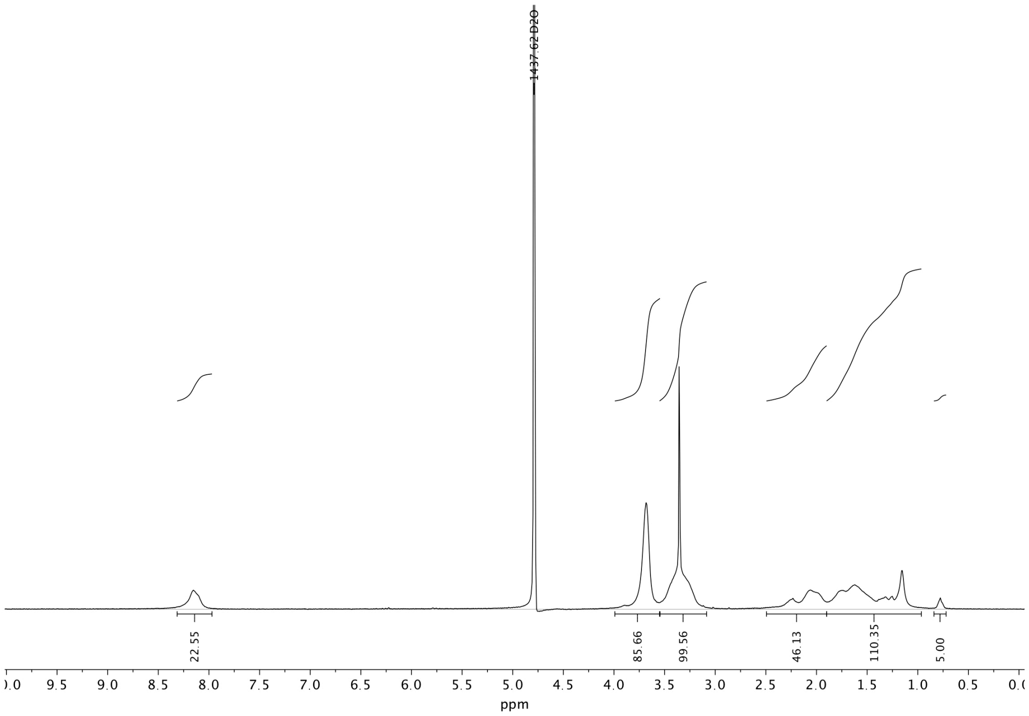

Figure S8. – ^{1}H NMR spectrum of PHEA50

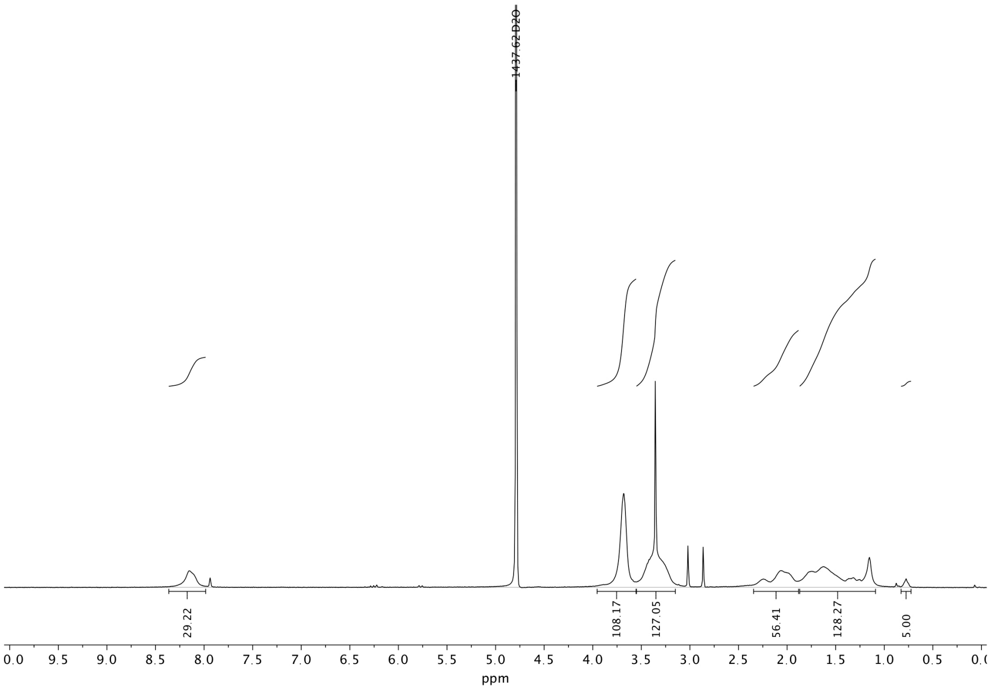

Figure S9. – ^{1}H NMR spectrum of PHEA58

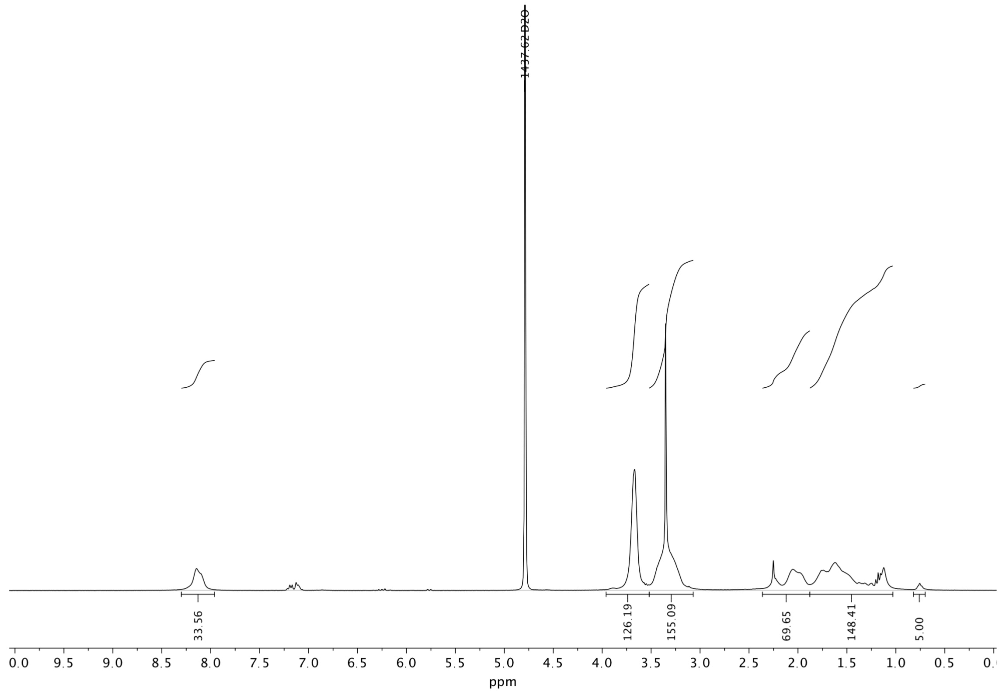

Figure S10. – ^{1}H NMR spectrum of PHEA72

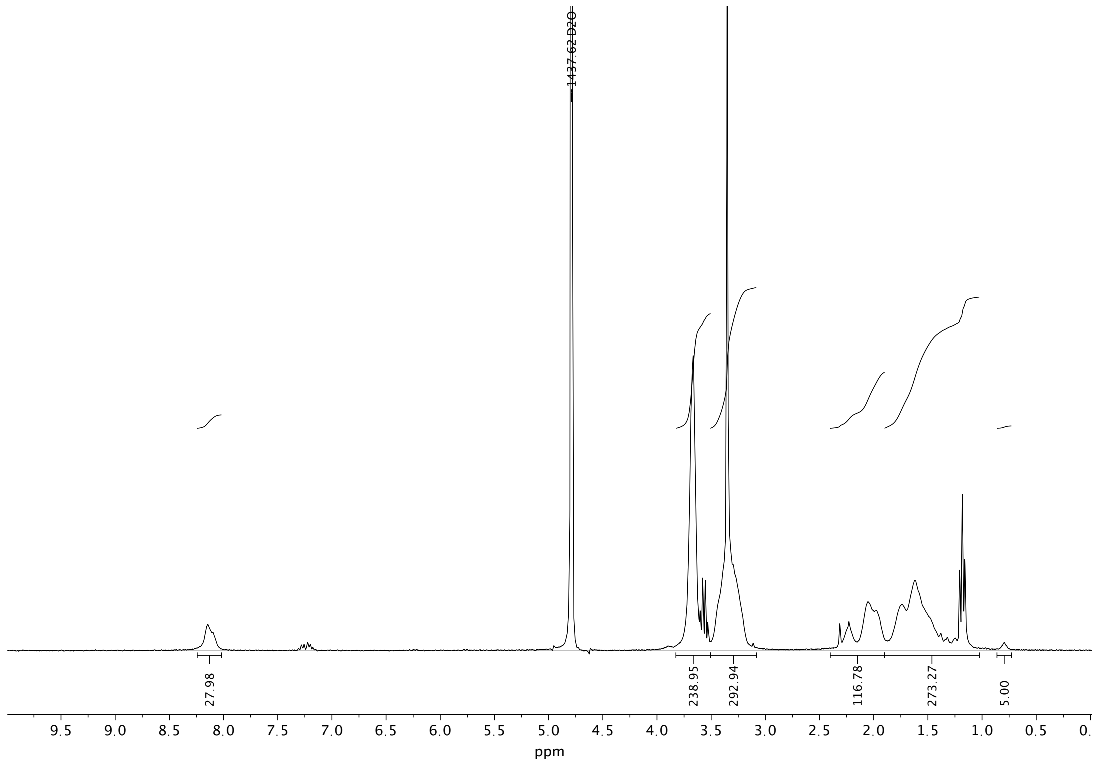

Figure S11. – ^{1}H NMR spectrum of PHEA110

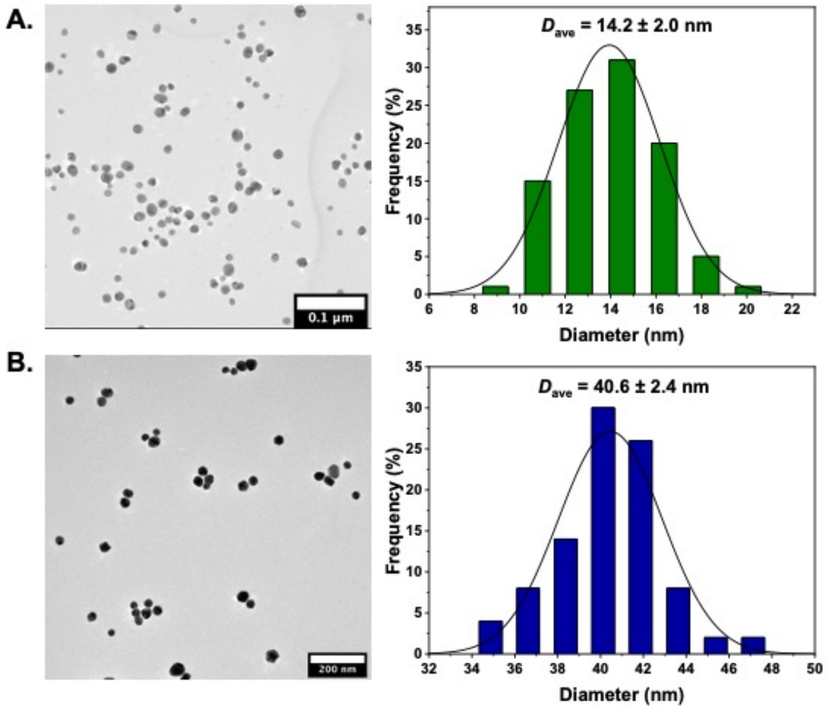

Figure S12. – TEM images (Left) and histograms (right) of citrate stabilized AuNPs. A) 16 nm AuNP and B) 40 nm AuNP. Histograms from analysis of over 100 particles.

SBA-Targeting AuNP Data

DLS and UV-vis Data

Table S1. – Nanoparticles Synthesised and Characterisation

Particle	Generation[a]	λ_{SPR}[b] (nm)	$\lambda_{SPR}/\lambda_{450}$[b] (nm)	DLS[c] (nm)
$AuNP_{16}$	1	518	1.56	20.69 ±0.8
$100\%GalPHEA_{26}@AuNP_{16}$	1	525	1.40	94.11 ±0.7
$66\%GalPHEA_{26}@AuNP_{16}$	1	526	1.81	142.2 ±20.2
$33\%GalPHEA_{26}@AuNP_{16}$	1	526	1.45	188.6 ±25.3
$0\%GalPHEA_{26}@AuNP_{16}$	1	528	1.49	293.9 ±35.1
$100\%GalPHEA_{40}@AuNP_{16}$	1	524	1.49	34.71 ±1.4
$66\%GalPHEA_{40}@AuNP_{16}$	1	525	1.48	54.81 ±0.8
$33\%GalPHEA_{40}@AuNP_{16}$	1	524	1.43	105.36 ±13.9
$0\%GalPHEA_{40}@AuNP_{16}$	1	523	1.51	136.3 ±36.5
$100\%GalPHEA_{72}@AuNP_{16}$	1	524	1.49	38.29 ±2.5
$66\%GalPHEA_{72}@AuNP_{16}$	1	524	1.49	62.45 ±5.7
$33\%GalPHEA_{72}@AuNP_{16}$	1	523	1.46	75.53 ±2.4
$0\%GalPHEA_{72}@AuNP_{16}$	1	524	1.45	103.9 ±6.0
$100\%GalPHEA_{110}@AuNP_{16}$	1	524	1.43	40.2 ±2.0
$66\%GalPHEA_{110}@AuNP_{16}$	1	524	1.46	66.26 ±7.9

33%GalPHEA$_{110}$@AuNP$_{16}$	1	524	1.43	89.57 ±8.5
0%GalPHEA$_{110}$@AuNP$_{16}$	1	522	1.51	110.8 ±3.8
AuNP$_{40}$	4	526	1.65	41.63 ±0.7
100%GalPHEA$_{26}$@AuNP$_{40}$	4	Unstable		
66%GalPHEA$_{26}$@AuNP$_{40}$	4	Unstable		
33%GalPHEA$_{26}$@AuNP$_{40}$	4	Unstable		
0%GalPHEA$_{26}$@AuNP$_{40}$	4	Unstable		
100%GalPHEA$_{40}$@AuNP$_{40}$	4	530	1.62	91.4 ±10.5
66%GalPHEA$_{40}$@AuNP$_{40}$	4	Unstable		
33%GalPHEA$_{40}$@AuNP$_{40}$	4	Unstable		
0%GalPHEA$_{40}$@AuNP$_{40}$	4	Unstable		
100%GalPHEA$_{72}$@AuNP$_{40}$	4	533	1.49	126.3 ±2.4
66%GalPHEA$_{72}$@AuNP$_{40}$	4	533	1.43	91.16 ±1.7
33%GalPHEA$_{72}$@AuNP$_{40}$	4	533	1.36	97.82 ±4.6
0%GalPHEA$_{72}$@AuNP$_{40}$	4	535	1.37	89.85 ±3.5
100%GalPHEA$_{110}$@AuNP$_{40}$	4	533	1.60	120.6 ±5.1
66%GalPHEA$_{110}$@AuNP$_{40}$	4	534	1.40	100.7 ±4.7
33%GalPHEA$_{110}$@AuNP$_{40}$	4	534	1.38	92.29 ±3.2
0%GalPHEA$_{110}$@AuNP$_{40}$	4	531	1.37	97.79 ±3.7

[a]Generation of nanoparticles used from the seeding synthetic methodology. [b]Maximum absorption wavelength from the surface plasmon resonance band of the particles and characteristic ratio. [c]Diameter from dynamic light scattering ± standard error from three measurements.

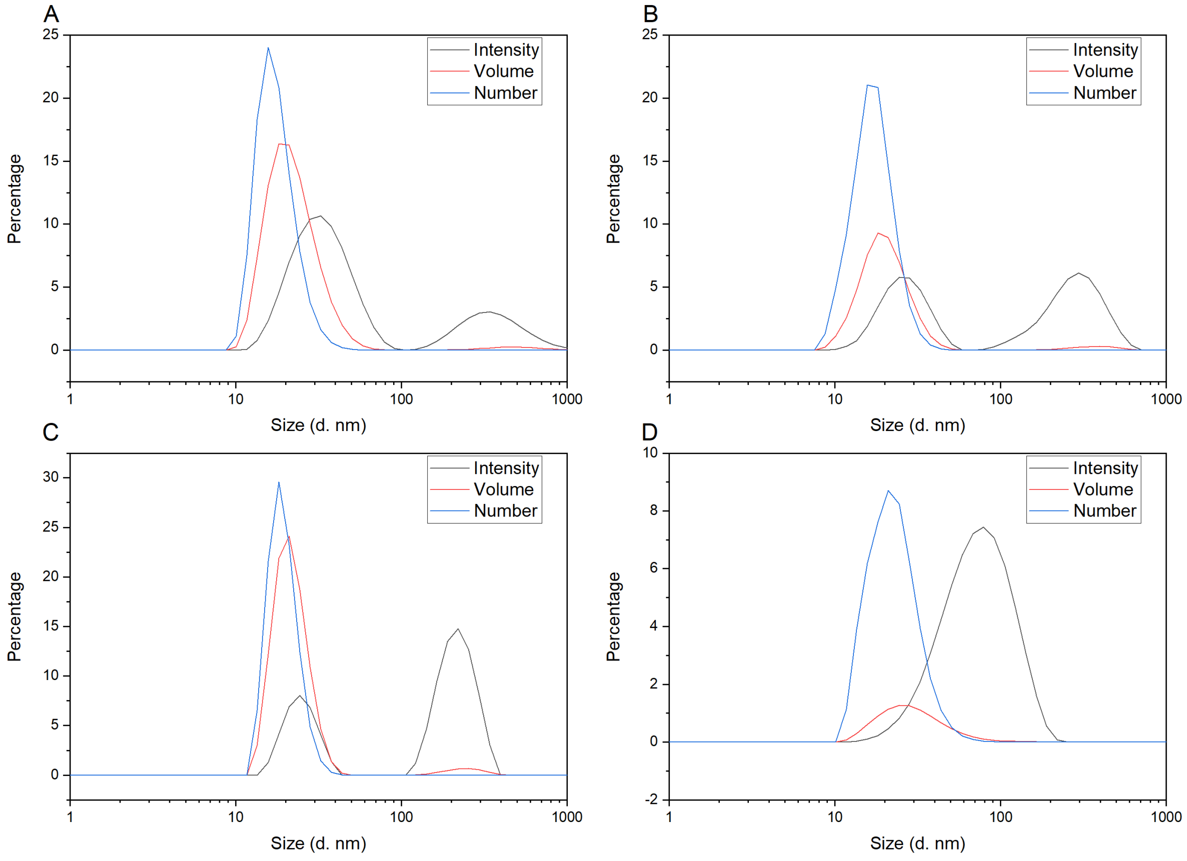

Figure S13. – DLS data for $GalPHEA_{26}@AuNP_{16}$ by percentage glycan functionalisation A) 100% B) 66% C) 33% D) 0%

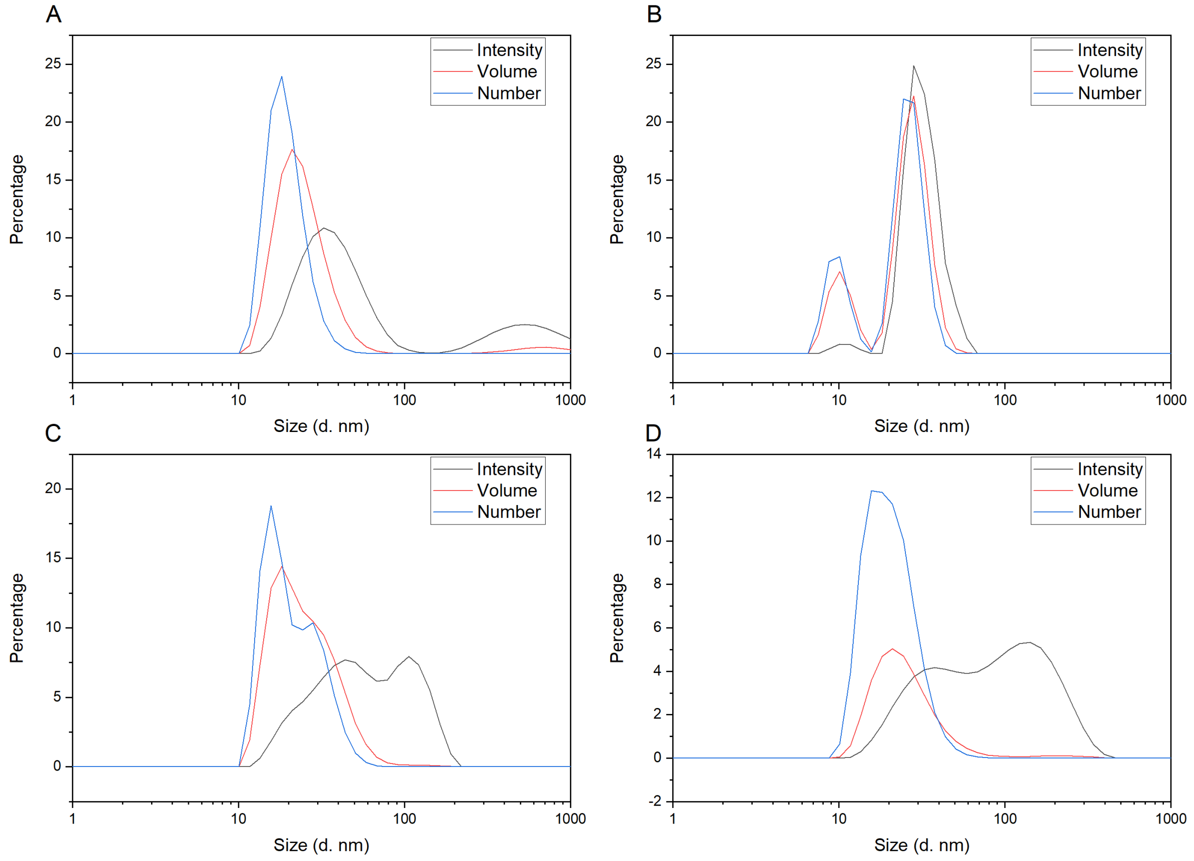

Figure S14. – DLS data for GalPHEA$_{40}$@AuNP$_{16}$ by percentage glycan functionalisation A) 100% B) 66% C) 33% D) 0%

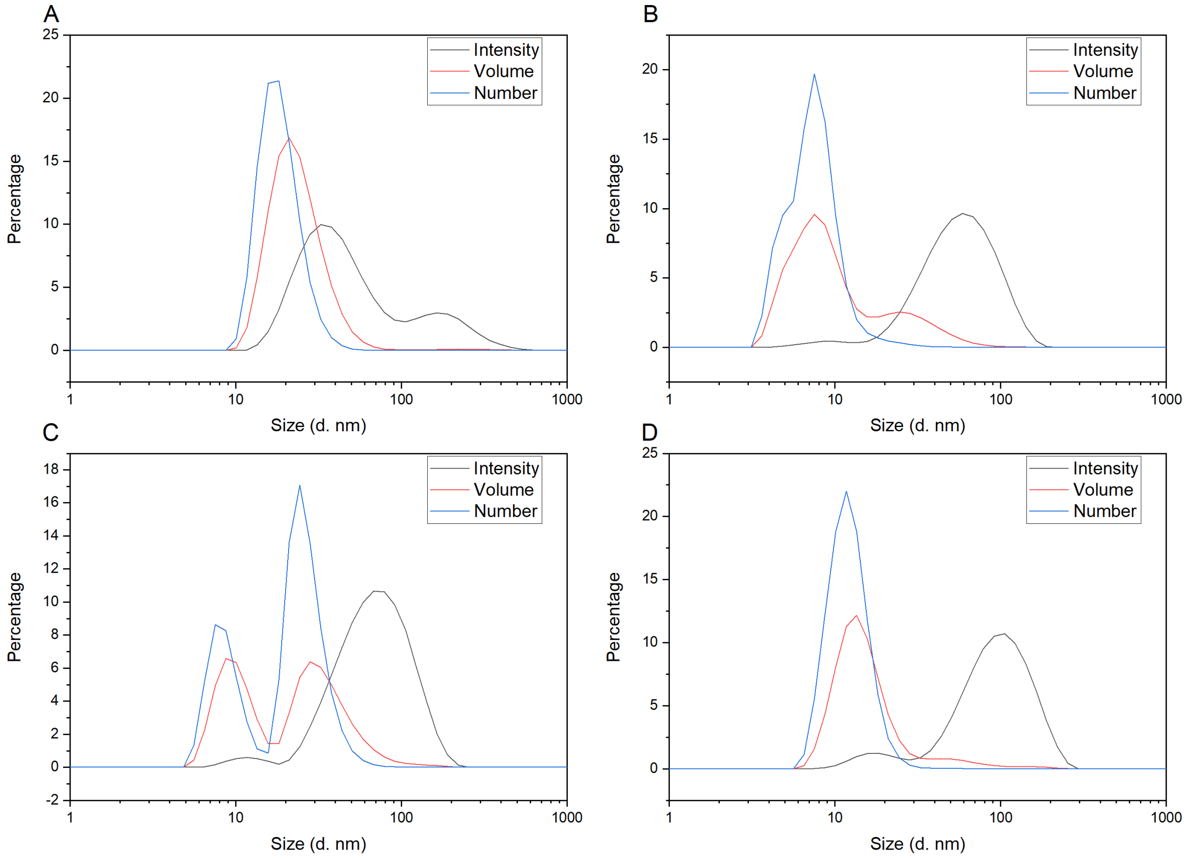

Figure S15. – DLS data for GalPHEA$_{72}$@AuNP$_{16}$ by percentage glycan functionalisation A) 100% B) 66% C) 33% D) 0%

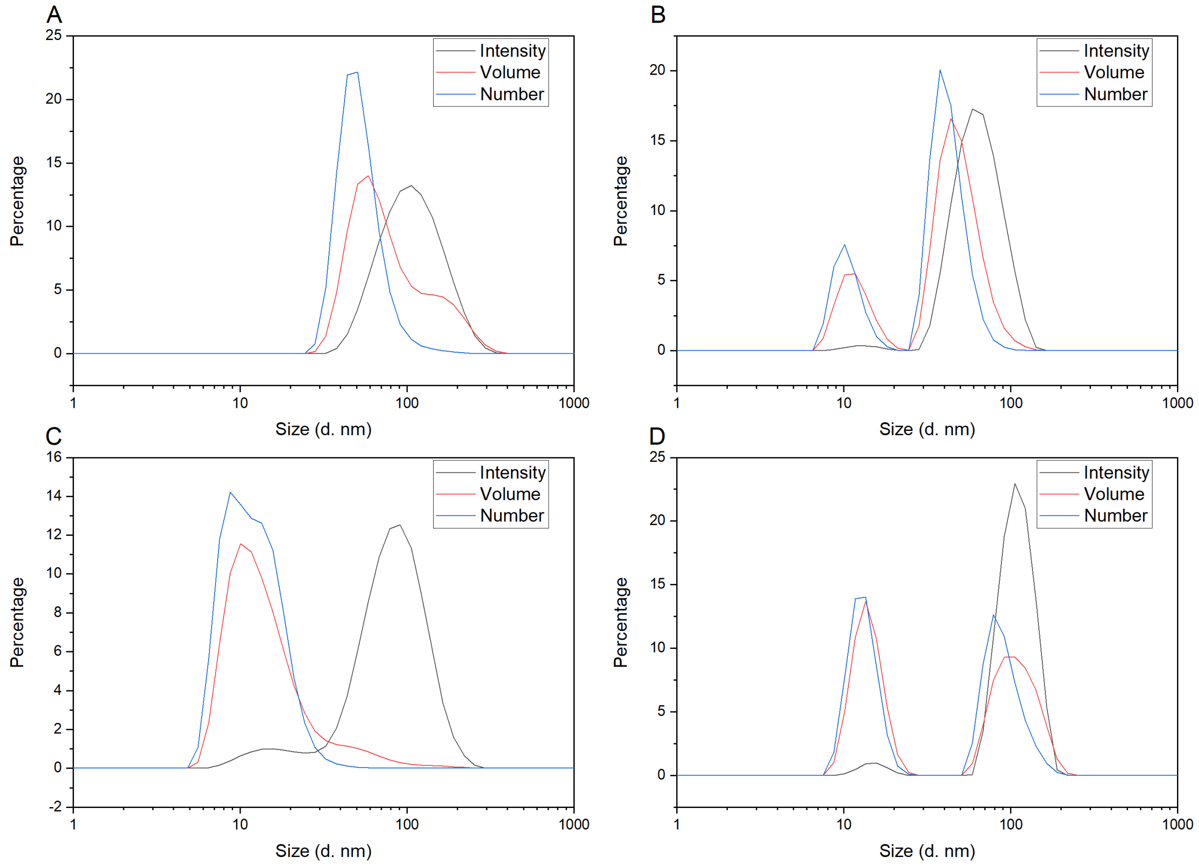

Figure S16. – DLS data for $GalPHEA_{110}@AuNP_{16}$ by percentage glycan functionalisation A) 100% B) 66% C) 33% D) 0%

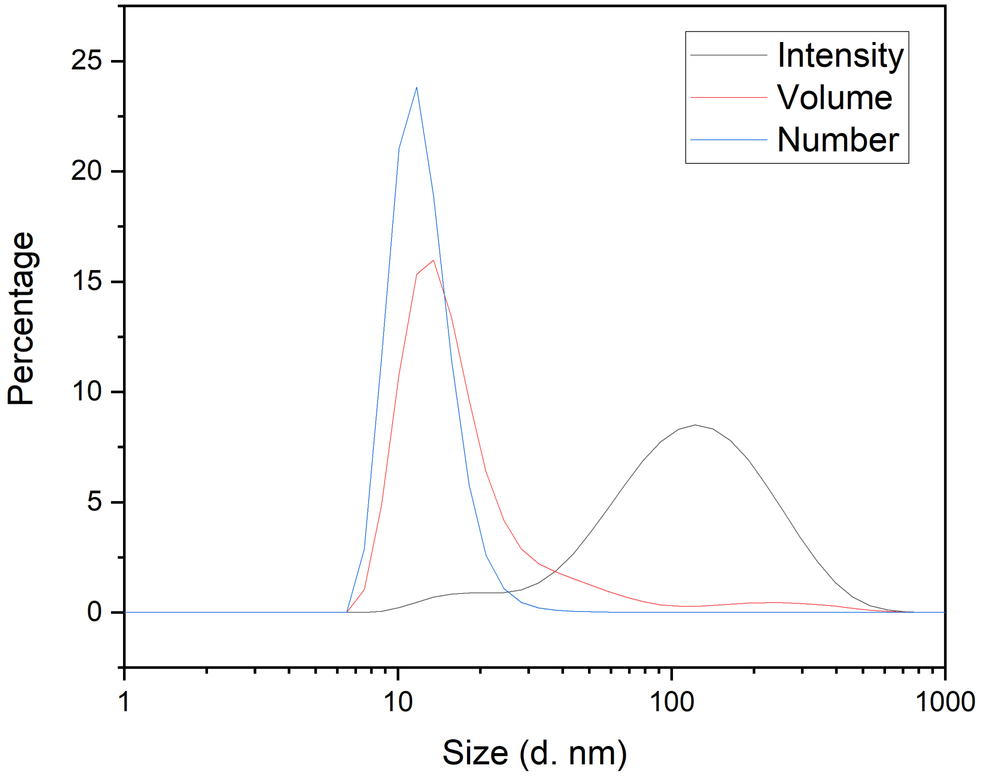

Figure S17. – DLS data for GalPHEA$_{40}$@AuNP$_{40}$ percentage glycan functionalisation 100%

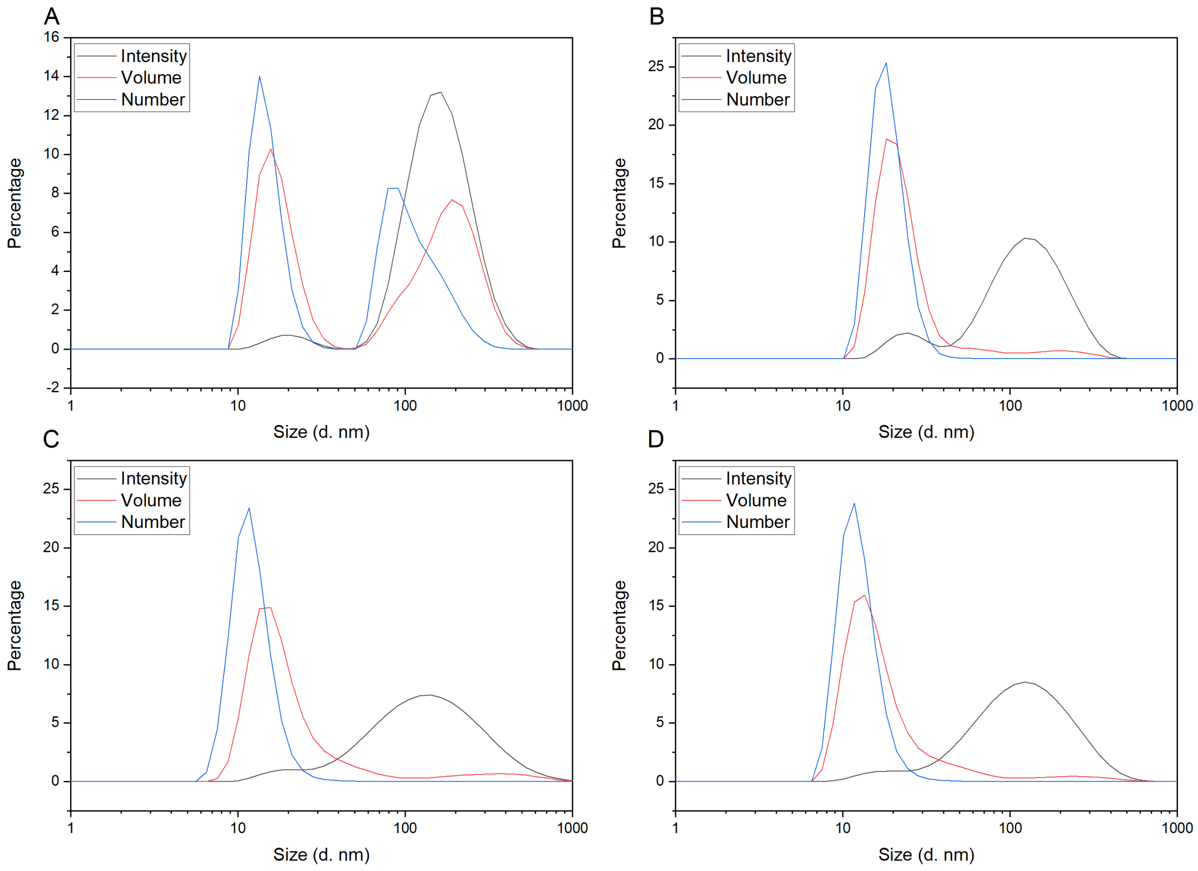

Figure S18. – DLS data for $GalPHEA_{72}@AuNP_{40}$ by percentage glycan functionalisation A) 100% B) 66% C) 33% D) 0%

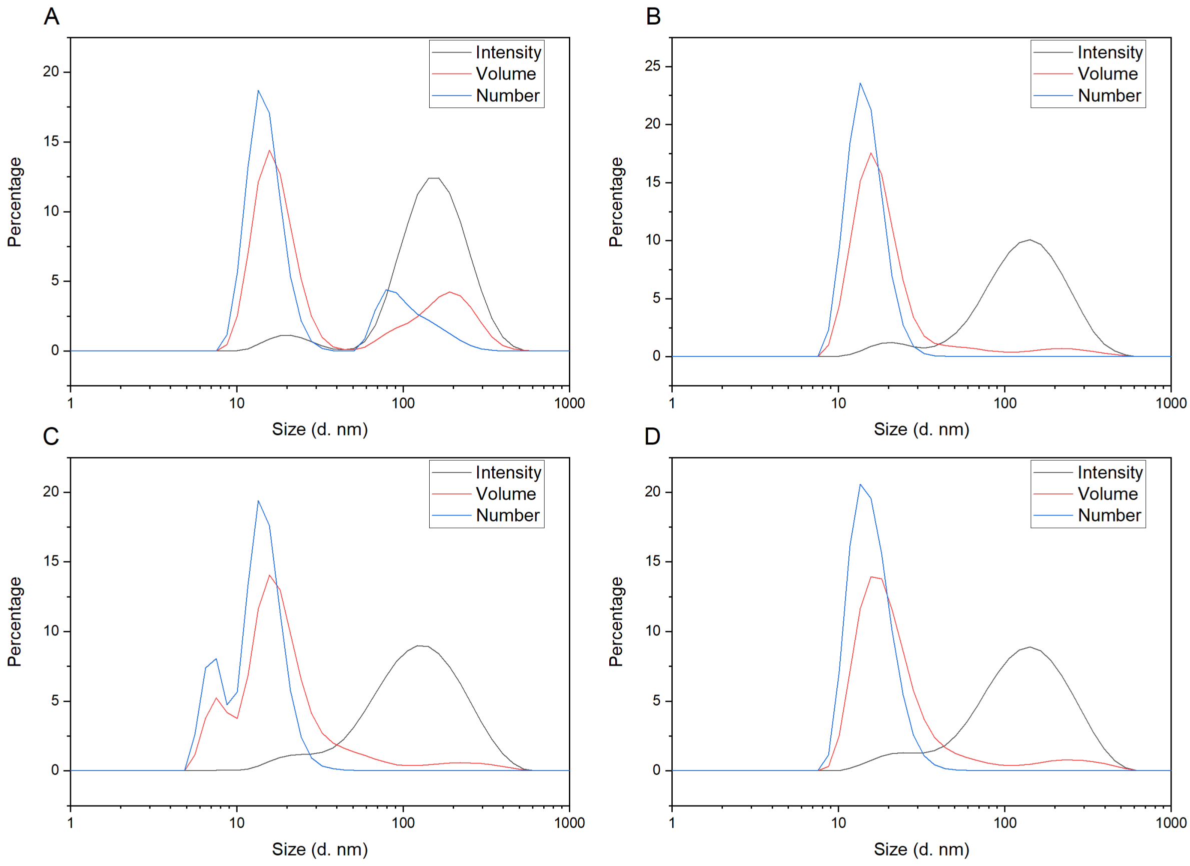

Figure S19. – DLS data for GalPHEA$_{110}$@AuNP$_{40}$ by percentage glycan functionalisation A) 100% B) 66% C) 33% D) 0%

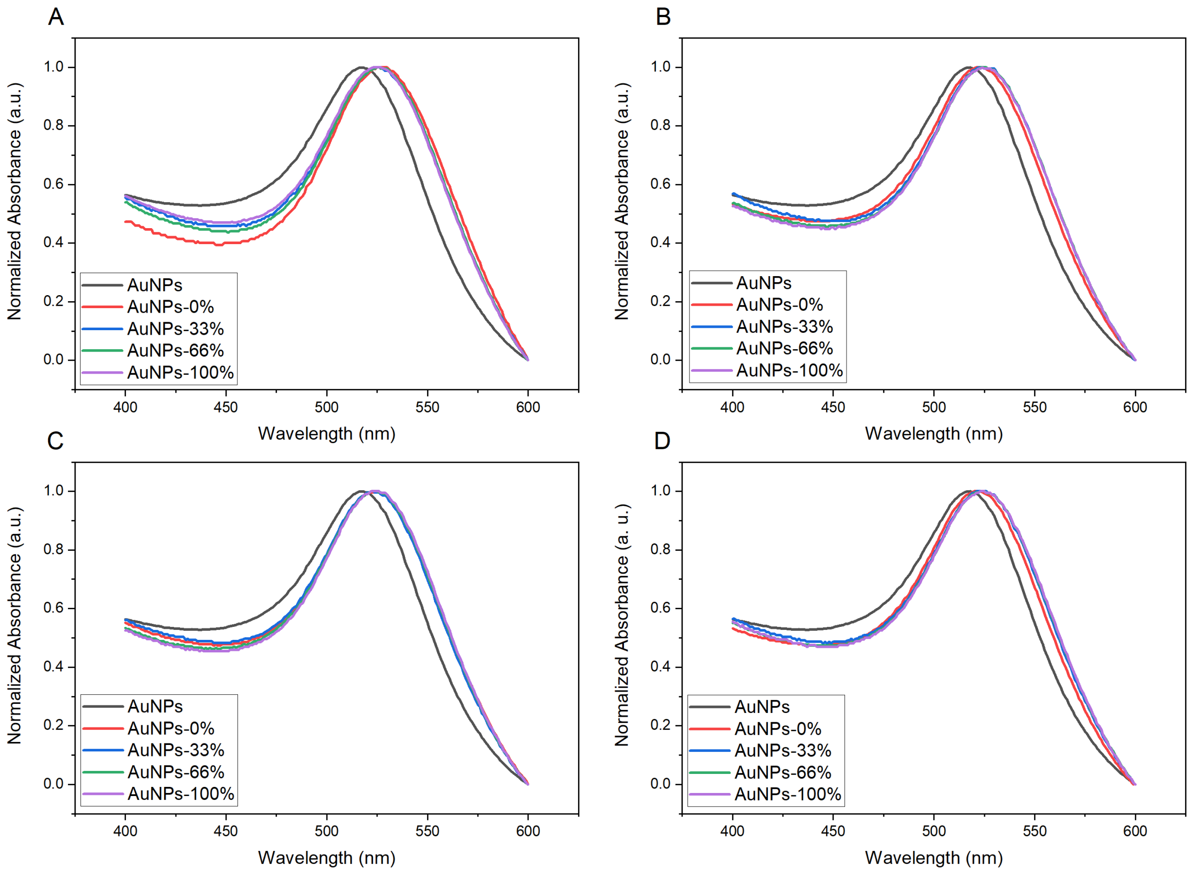

Figure S20. – UV-vis for 16nm AuNPs by degree of polymerisation A) 26 B) 40 C) 72 D) 110

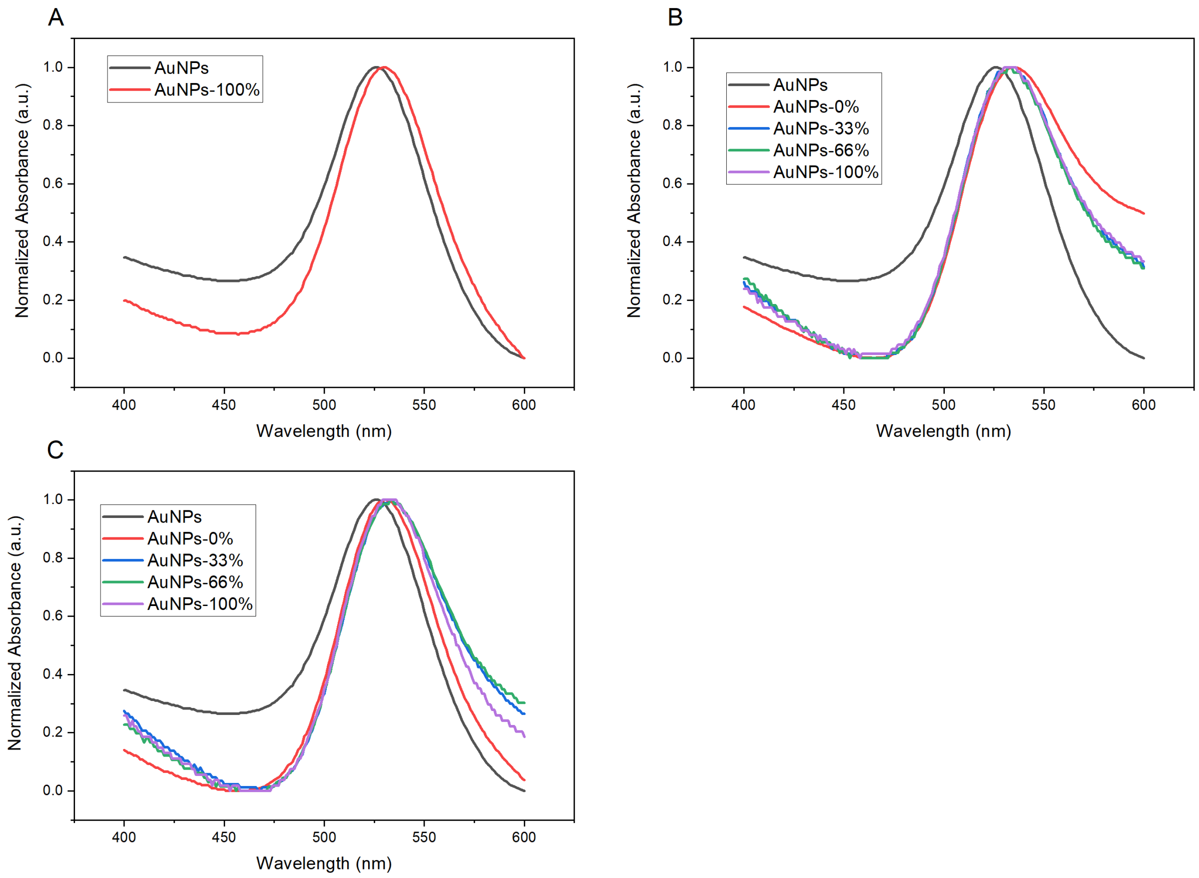

Figure S21. – UV-vis for 40nm AuNPs by degree of polymerisation A) 40 B) 72 C) 110

Lateral Flow Strip Data

Percentage sugar functionalisation (%)	BSA	BSA-Gal	SBA + BSA-Gal	UEA + BSA-Gal
0	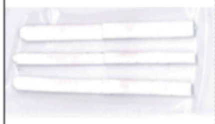	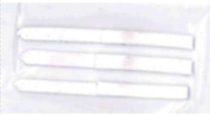	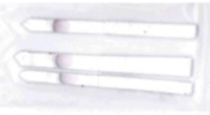	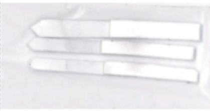

Table S2. – Scans of lateral flow strips using $AuNP_{16}$. Test lines are unfunctionalised BSA (BSA, 1 mg.mL^{-1}), and Galα1-3Galβ1-4GlcNAc-BSA (BSA-Gal, 1 mg.mL^{-1}) with (or without) lectins in solution (SBA or UEA, 0.05 mg.mL^{-1}).

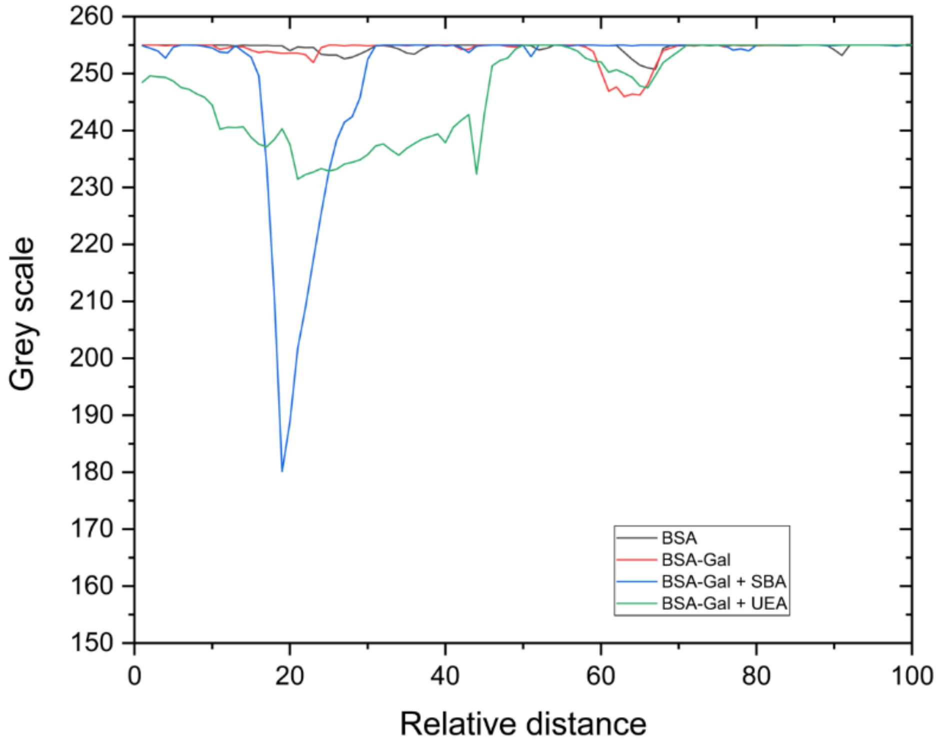

Figure S22. – Analysis of scanned lateral flow strips using $AuNP_{16}$. Test lines are unfunctionalised BSA (BSA, 1 mg.mL^{-1}), and Galα1-3Galβ1-4GlcNAc-BSA (BSA-Gal, 1 mg.mL^{-1}) with (or without) lectins in solution (SBA or UEA, 0.05 mg.mL^{-1}).

Percentage sugar functionalisation (%)	BSA	BSA-Gal	SBA + BSA-Gal	UEA + BSA-Gal
100	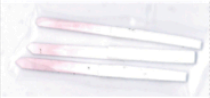	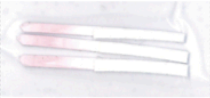	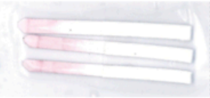	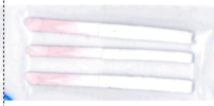
66	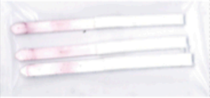	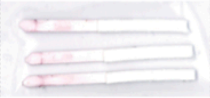		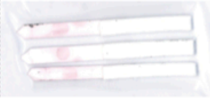
33	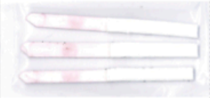	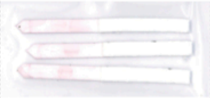	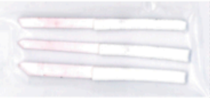	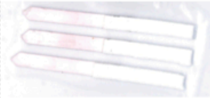
0	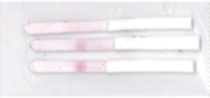	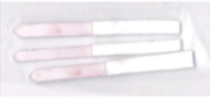	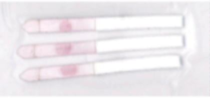	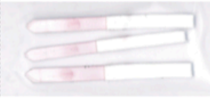

Table S3. – Scans of lateral flow strips using GalPHEA$_{26}$@AuNP$_{16}$ particles of varying glycan functionalisation. Test lines are unfunctionalised BSA (BSA, 1 mg.mL^{-1}), and Galα1-3Galβ1-4GlcNAc-BSA (BSA-Gal, 1 mg.mL^{-1}) with (or without) lectins in solution (SBA or UEA, 0.05 mg.mL^{-1}).

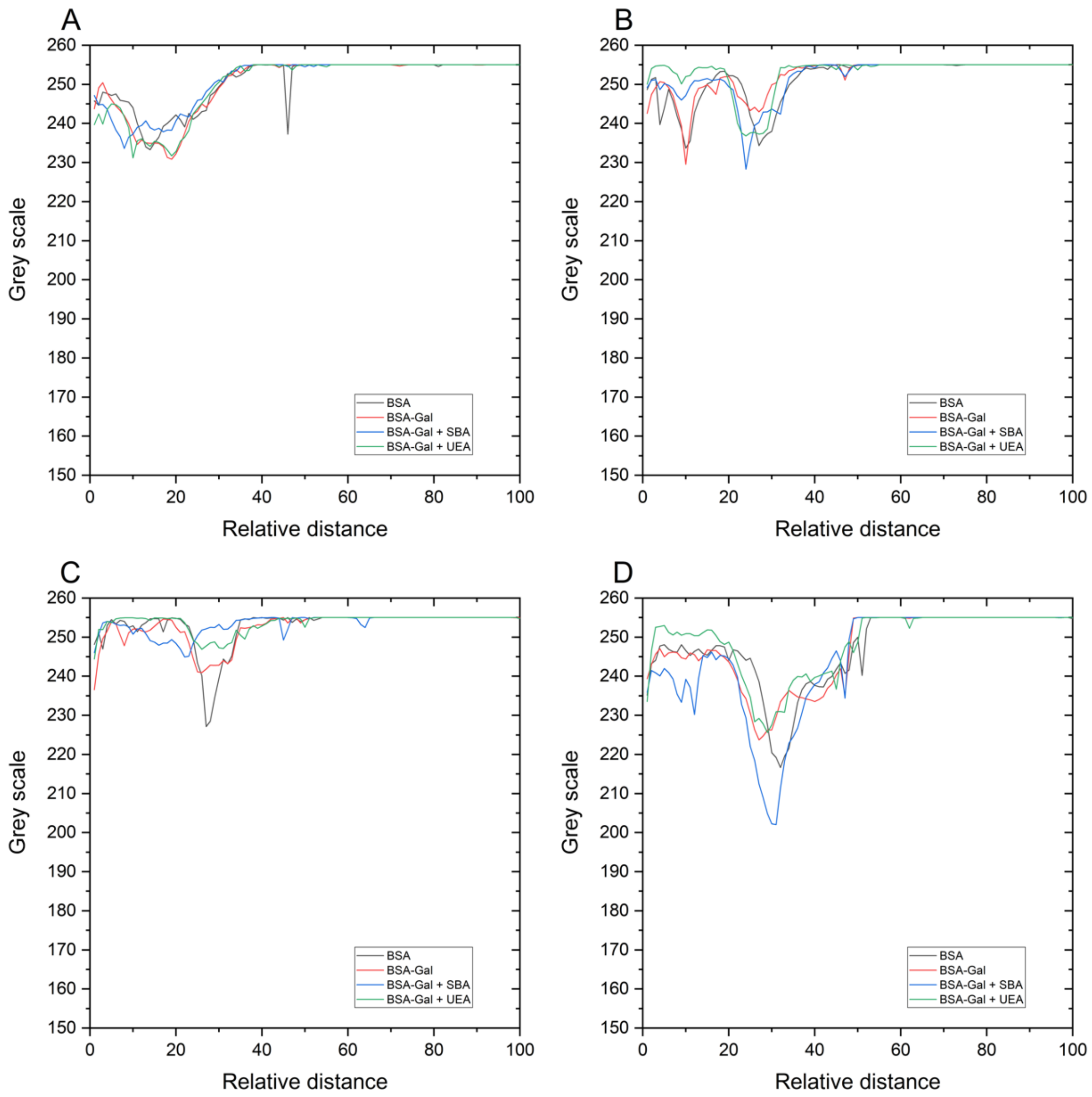

Figure S23. – Analysis of scanned lateral flow strips using GalPHEA$_{26}$@AuNP$_{16}$ particles of varying glycan functionalisation A) 100%, B) 66%, C) 33% and D) 0%. Test lines are unfunctionalised BSA (BSA, 1 mg.mL^{-1}), and Galα1-3Galβ1-4GlcNAc-BSA (BSA-Gal, 1 mg.mL^{-1}) with (or without) lectins in solution (SBA or UEA, 0.05 mg.mL^{-1}).

Percentage sugar functionalisation (%)	BSA	BSA-Gal	SBA + BSA-Gal	UEA + BSA-Gal
100	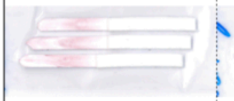	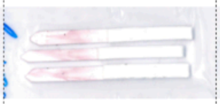	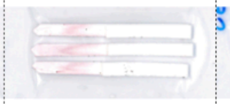	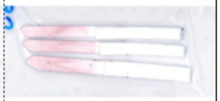
66	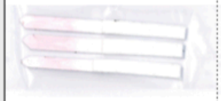	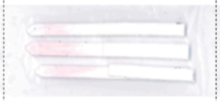	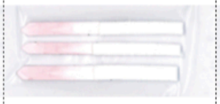	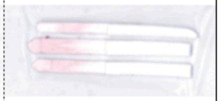
33	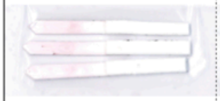	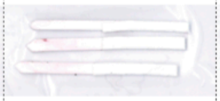	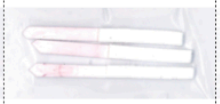	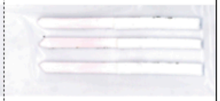
0	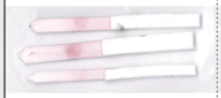	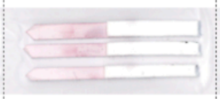	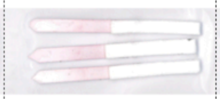	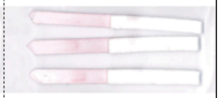

Table S4. – Scans of lateral flow strips using GalPHEA$_{40}$@AuNP$_{16}$ particles of varying glycan functionalisation. Test lines are unfunctionalised BSA (BSA, 1 mg.mL^{-1}), and Galα1-3Galβ1-4GlcNAc-BSA (BSA-Gal, 1 mg.mL^{-1}) with (or without) lectins in solution (SBA or UEA, 0.05 mg.mL^{-1}).

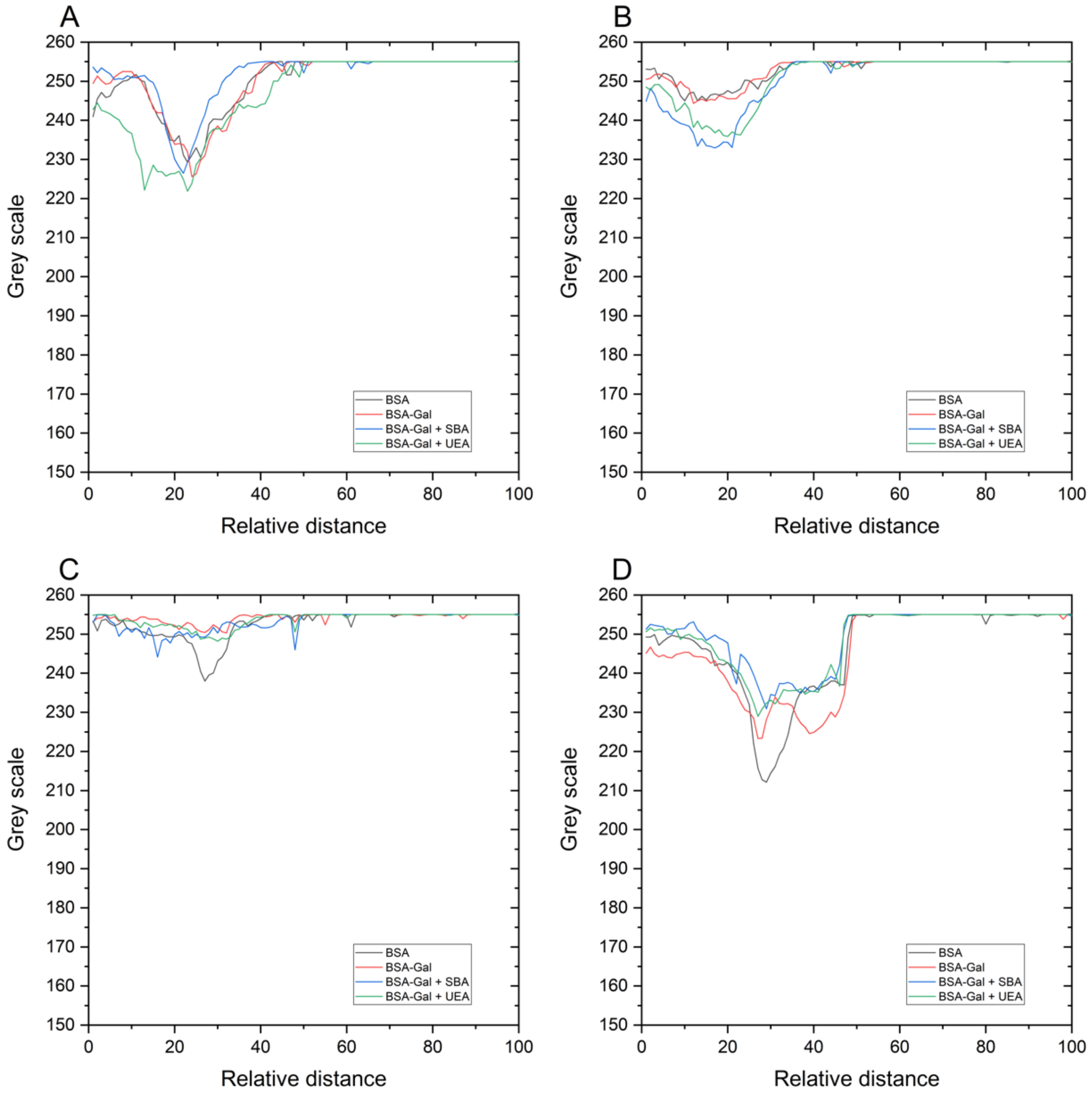

Figure S24. – Analysis of scanned lateral flow strips using GalPHEA$_{40}$@AuNP$_{16}$ particles of varying glycan functionalisation A) 100%, B) 66%, C) 33% and D) 0%. Test lines are unfunctionalised BSA (BSA, 1 mg.mL^{-1}), and Galα1-3Galβ1-4GlcNAc-BSA (BSA-Gal, 1 mg.mL^{-1}) with (or without) lectins in solution (SBA or UEA, 0.05 mg.mL^{-1}).

Percentage sugar functionalisation (%)	BSA	BSA-Gal	SBA + BSA-Gal	UEA + BSA-Gal
100	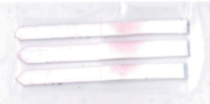	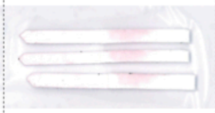	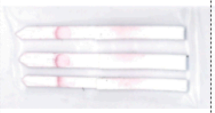	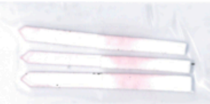
66	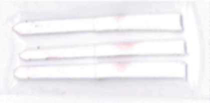	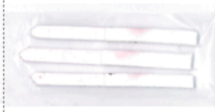	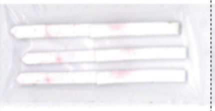	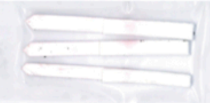
33	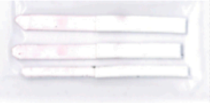	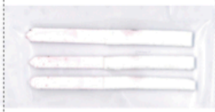	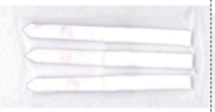	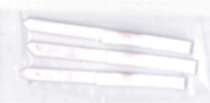
0	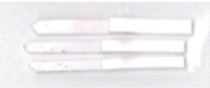	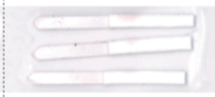	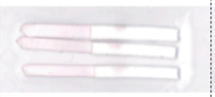	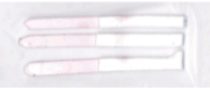

Table S5. – Scans of lateral flow strips using GalPHEA$_{72}$@AuNP$_{16}$ particles of varying glycan functionalisation. Test lines are unfunctionalised BSA (BSA, 1 mg.mL^{-1}), and Galα1-3Galβ1-4GlcNAc-BSA (BSA-Gal, 1 mg.mL^{-1}) with (or without) lectins in solution (SBA or UEA, 0.05 mg.mL^{-1}).

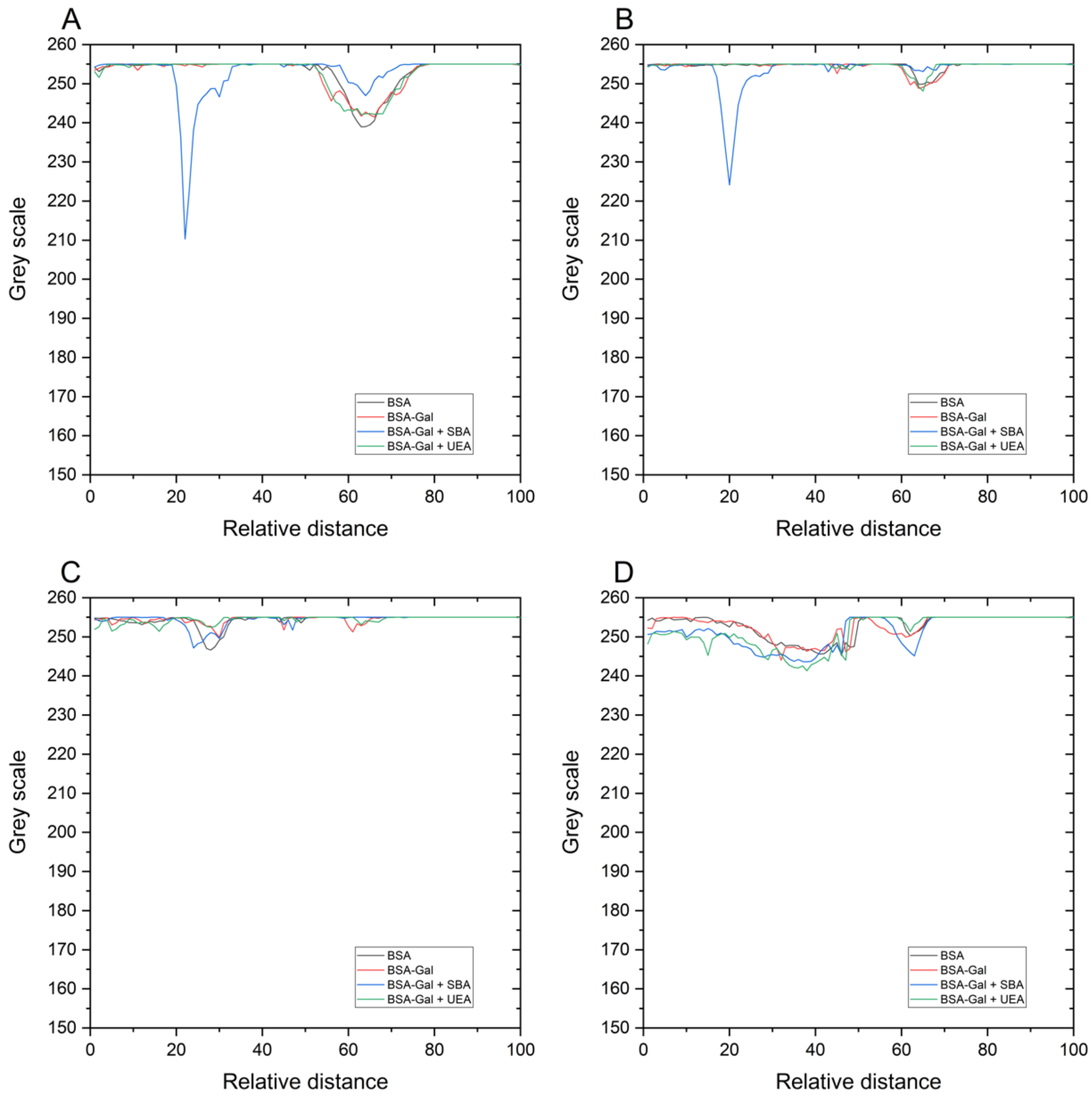

Figure S25. – Analysis of scanned lateral flow strips using GalPHEA$_{72}$@AuNP$_{16}$ particles of varying glycan functionalisation A) 100%, B) 66%, C) 33% and D) 0%. Test lines are unfunctionalised BSA (BSA, 1 mg.mL^{-1}), and Galα1-3Galβ1-4GlcNAc-BSA (BSA-Gal, 1 mg.mL^{-1}) with (or without) lectins (SBA or UEA, 0.05 mg.mL^{-1}).

Percentage sugar functionalisation (%)	BSA	BSA-Gal	SBA + BSA-Gal	UEA + BSA-Gal
100	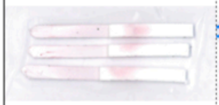	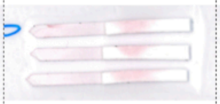	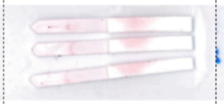	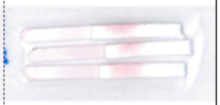
66	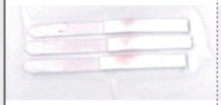	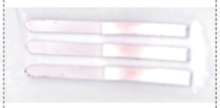	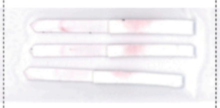	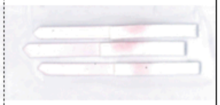
33	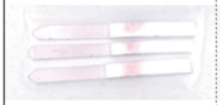	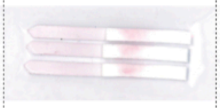	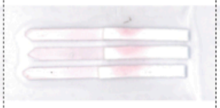	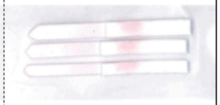
0	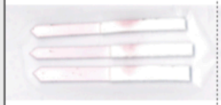	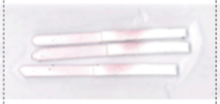	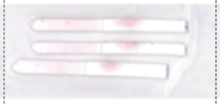	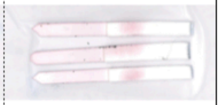

Table S6. – Scans of lateral flow strips using GalPHEA$_{110}$@AuNP$_{16}$ particles of varying glycan functionalisation. Test lines are unfunctionalised BSA (BSA, 1 mg.mL^{-1}), and Galα1-3Galβ1-4GlcNAc-BSA (BSA-Gal, 1 mg.mL^{-1}) with (or without) lectins in solution (SBA or UEA, 0.05 mg.mL^{-1}).

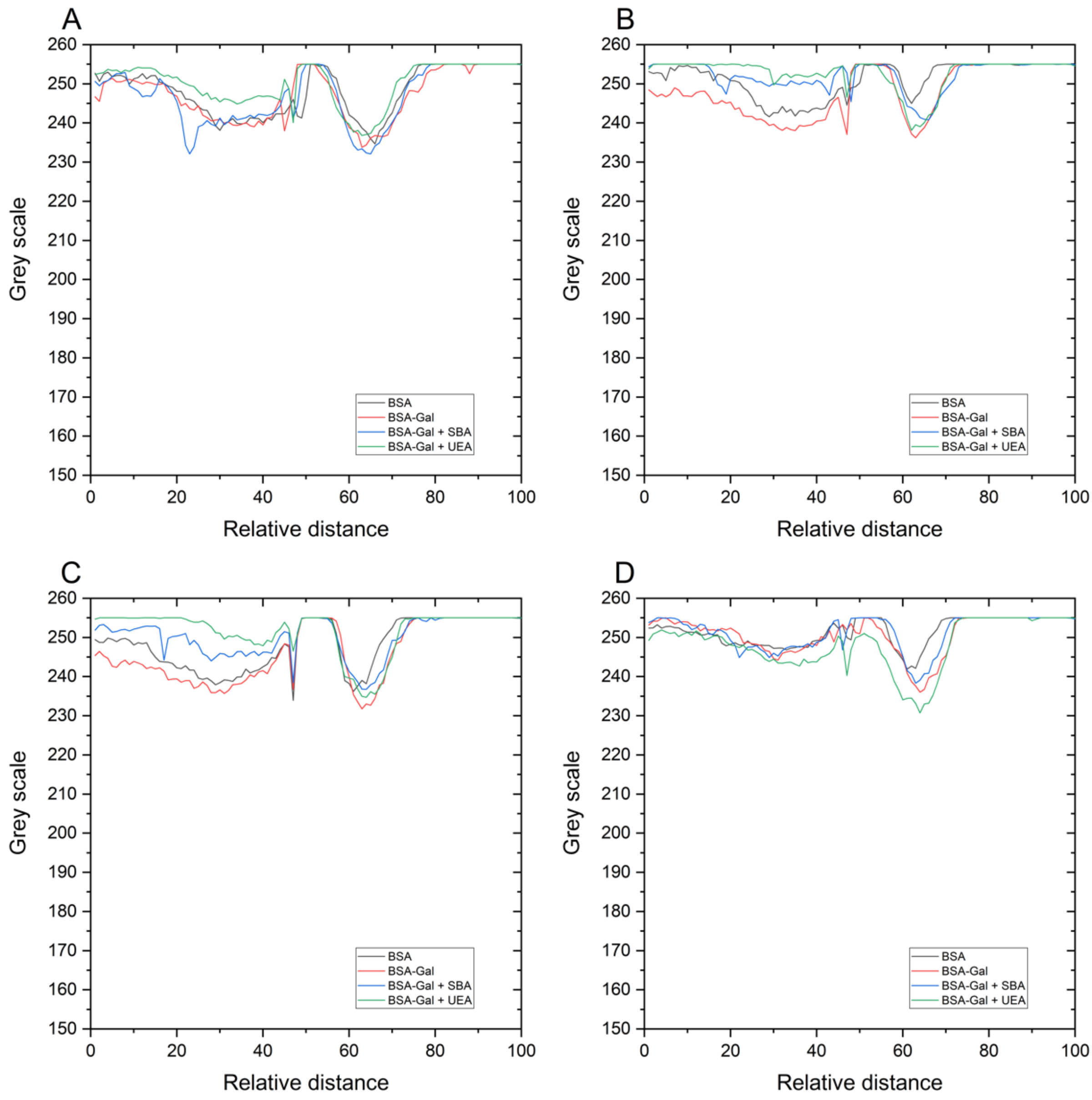

Figure S26. – Analysis of scanned lateral flow strips using GalPHEA$_{110}$@AuNP$_{16}$ particles of varying glycan functionalisation A) 100%, B) 66%, C) 33% and D) 0%. Test lines are unfunctionalised BSA (BSA, 1 mg.mL^{-1}), and Galα1-3Galβ1-4GlcNAc-BSA (BSA-Gal, 1 mg.mL^{-1}) with (or without) lectins (SBA or UEA, 0.05 mg.mL^{-1}).

AuNP System	Signal:Noise
$AuNP_{16}$ vs. BSA	252.6
$AuNP_{16}$ vs. BSA-Gal + SBA	180.1
$AuNP_{16}$ vs. BSA-Gal + UEA	231.5
100% $GalPHEA_{26}$@$AuNP_{16}$ vs. BSA	1.6
100% $GalPHEA_{26}$@$AuNP_{16}$ vs. BSA-Gal + SBA	1.4
100% $GalPHEA_{26}$@$AuNP_{16}$ vs. BSA-Gal + UEA	1.8
66% $GalPHEA_{26}$@$AuNP_{16}$ vs. BSA	3.3
66% $GalPHEA_{26}$@$AuNP_{16}$ vs. BSA-Gal + SBA	4.3
66% $GalPHEA_{26}$@$AuNP_{16}$ vs. BSA-Gal + UEA	2.9
33% $GalPHEA_{26}$@$AuNP_{16}$ vs. BSA	3.9
33% $GalPHEA_{26}$@$AuNP_{16}$ vs. BSA-Gal + SBA	1.4
33% $GalPHEA_{26}$@$AuNP_{16}$ vs. BSA-Gal + UEA	1.1
0% $GalPHEA_{26}$@$AuNP_{16}$ vs. BSA	2.0
0% $GalPHEA_{26}$@$AuNP_{16}$ vs. BSA-Gal + SBA	2.8
0% $GalPHEA_{26}$@$AuNP_{16}$ vs. BSA-Gal + UEA	1.5
100% $GalPHEA_{40}$@$AuNP_{16}$ vs. BSA	1.4
100% $GalPHEA_{40}$@$AuNP_{16}$ vs. BSA-Gal + SBA	1.5
100% $GalPHEA_{40}$@$AuNP_{16}$ vs. BSA-Gal + UEA	1.8
66% $GalPHEA_{40}$@$AuNP_{16}$ vs. BSA	1.8
66% $GalPHEA_{40}$@$AuNP_{16}$ vs. BSA-Gal + SBA	3.9
66% $GalPHEA_{40}$@$AuNP_{16}$ vs. BSA-Gal + UEA	3.4
33% $GalPHEA_{40}$@$AuNP_{16}$ vs. BSA	6.3
33% $GalPHEA_{40}$@$AuNP_{16}$ vs. BSA-Gal + SBA	4.0
33% $GalPHEA_{40}$@$AuNP_{16}$ vs. BSA-Gal + UEA	2.5
0% $GalPHEA_{40}$@$AuNP_{16}$ vs. BSA	2.0
0% $GalPHEA_{40}$@$AuNP_{16}$ vs. BSA-Gal + SBA	1.1
0% $GalPHEA_{40}$@$AuNP_{16}$ vs. BSA-Gal + UEA	1.2
100% $GalPHEA_{72}$@$AuNP_{16}$ vs. BSA	2.5
100% $GalPHEA_{72}$@$AuNP_{16}$ vs. BSA-Gal + SBA	344.1
100% $GalPHEA_{72}$@$AuNP_{16}$ vs. BSA-Gal + UEA	1.0
66% $GalPHEA_{72}$@$AuNP_{16}$ vs. BSA	2.8

66% $GalPHEA_{72}@AuNP_{16}$ vs. BSA-Gal + SBA	254.3
66% $GalPHEA_{72}@AuNP_{16}$ vs. BSA-Gal + UEA	2.6
33% $GalPHEA_{72}@AuNP_{16}$ vs. BSA	7.4
33% $GalPHEA_{72}@AuNP_{16}$ vs. BSA-Gal + SBA	6.9
33% $GalPHEA_{72}@AuNP_{16}$ vs. BSA-Gal + UEA	3.2
0% $GalPHEA_{72}@AuNP_{16}$ vs. BSA	1.9
0% $GalPHEA_{72}@AuNP_{16}$ vs. BSA-Gal + SBA	2.9
0% $GalPHEA_{72}@AuNP_{16}$ vs. BSA-Gal + UEA	3.3
100% $GalPHEA_{110}@AuNP_{16}$ vs. BSA	1.5
100% $GalPHEA_{110}@AuNP_{16}$ vs. BSA-Gal + SBA	2.0
100% $GalPHEA_{110}@AuNP_{16}$ vs. BSA-Gal + UEA	0.9
66% $GalPHEA_{110}@AuNP_{16}$ vs. BSA	1.0
66% $GalPHEA_{110}@AuNP_{16}$ vs. BSA-Gal + SBA	0.6
66% $GalPHEA_{110}@AuNP_{16}$ vs. BSA-Gal + UEA	0.4
33% $GalPHEA_{110}@AuNP_{16}$ vs. BSA	1.0
33% $GalPHEA_{110}@AuNP_{16}$ vs. BSA-Gal + SBA	0.7
33% $GalPHEA_{110}@AuNP_{16}$ vs. BSA-Gal + UEA	0.3
0% $GalPHEA_{110}@AuNP_{16}$ vs. BSA	1.3
0% $GalPHEA_{110}@AuNP_{16}$ vs. BSA-Gal + SBA	1.6
0% $GalPHEA_{110}@AuNP_{16}$ vs. BSA-Gal + UEA	1.9

Table S7. – Signal to noise ratios of all AuNP16 systems

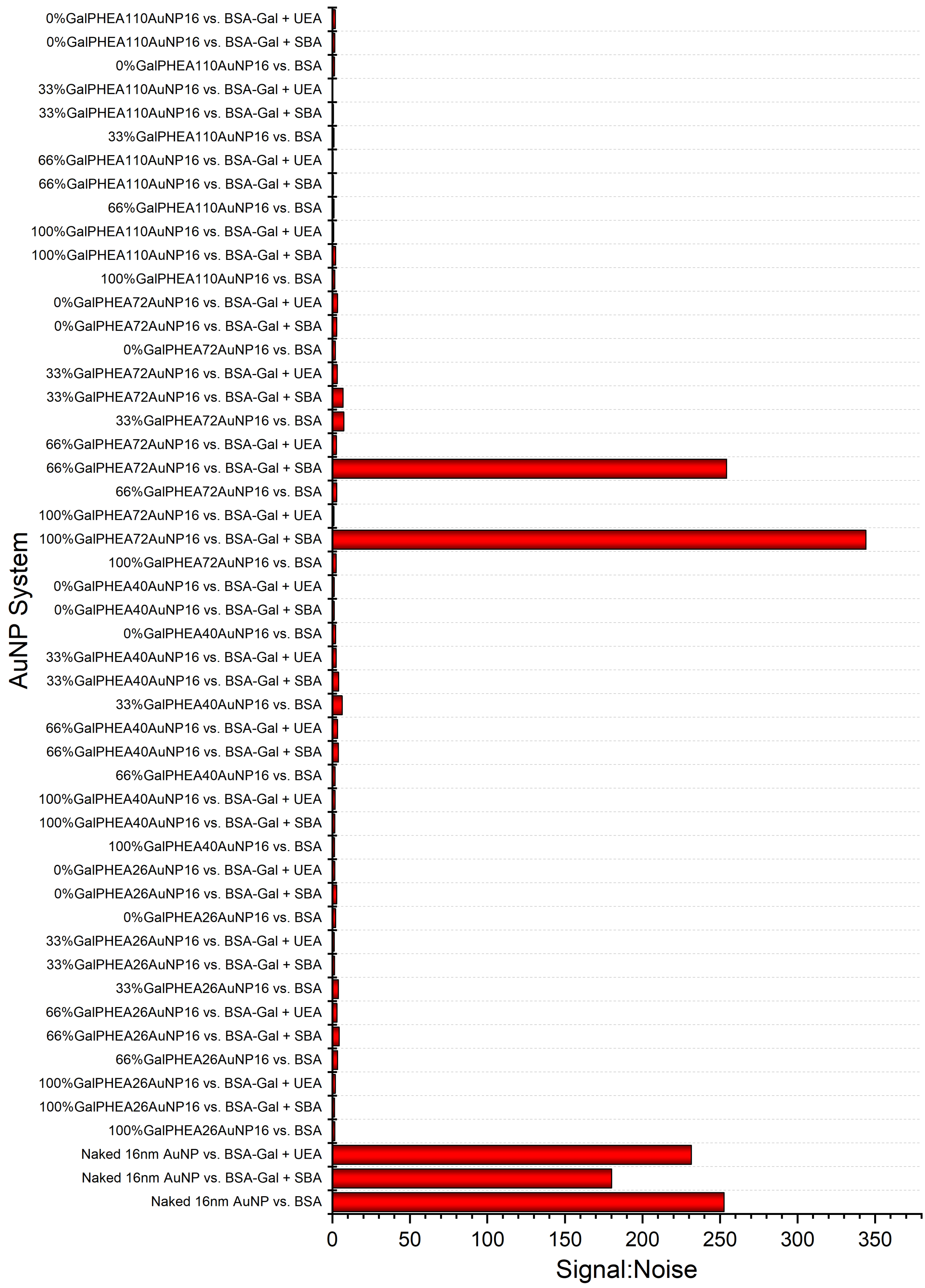

Figure S27. – Signal to noise ratios of all 16nm AuNPs

Percentage sugar functionalisation (%)	BSA	BSA-Gal	SBA + BSA-Gal	UEA + BSA-Gal
0	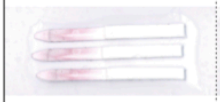	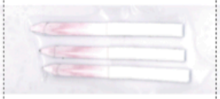	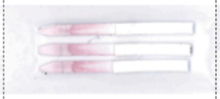	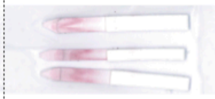

Table S8. – Scans of lateral flow strips using $AuNP_{40}$. Test lines are unfunctionalised BSA (BSA, 1 mg.mL^{-1}), and Galα1-3Galβ1-4GlcNAc-BSA (BSA-Gal, 1 mg.mL^{-1}) with (or without) lectins in solution (SBA or UEA, 0.05 mg.mL^{-1}).

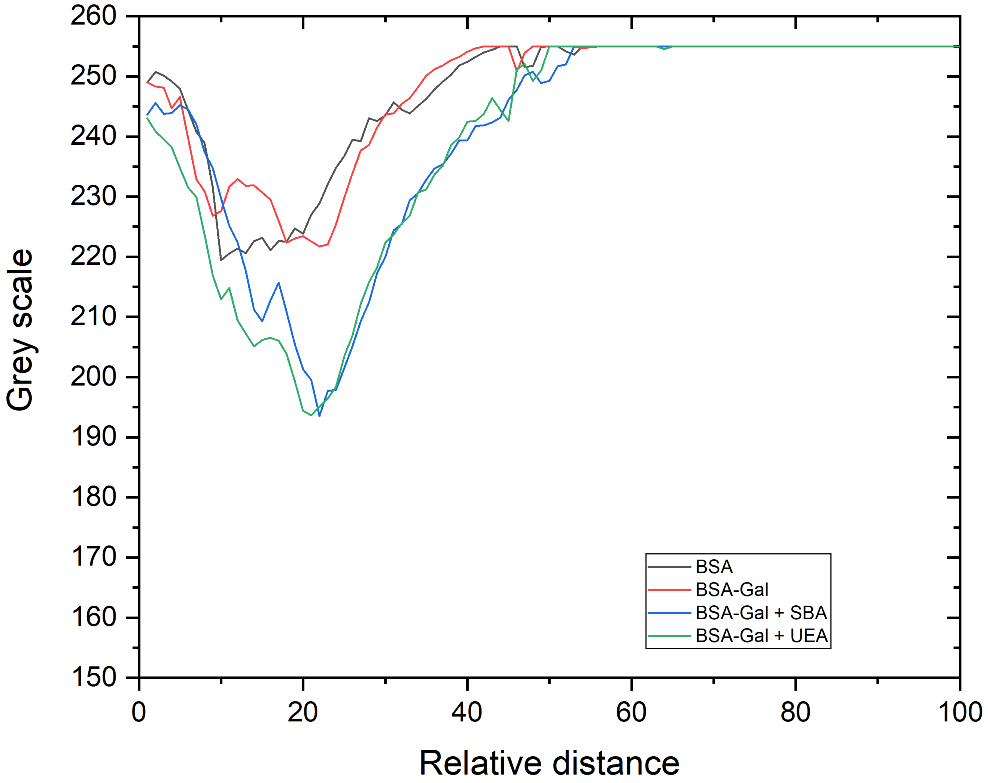

Figure S28. – Analysis of scanned lateral flow strips using $AuNP_{40}$. Test lines are unfunctionalised BSA (BSA, 1 mg.mL^{-1}), and Galα1-3Galβ1-4GlcNAc-BSA (BSA-Gal, 1 mg.mL^{-1}) with (or without) lectins in solution (SBA or UEA, 0.05 mg.mL^{-1}).

Percentage sugar functionalisation (%)	BSA	BSA-Gal	SBA + BSA-Gal	UEA + BSA-Gal
100	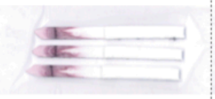	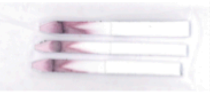	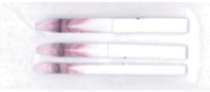	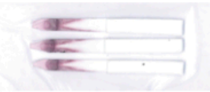

Table S9. – Scans of lateral flow strips using 100% GalPHEA$_{40}$@AuNP$_{40}$. Test lines are unfunctionalised BSA (BSA, 1 mg.mL^{-1}), and Galα1-3Galβ1-4GlcNAc-BSA (BSA-Gal, 1 mg.mL^{-1}) with (or without) lectins in solution (SBA or UEA, 0.05 mg.mL^{-1}).

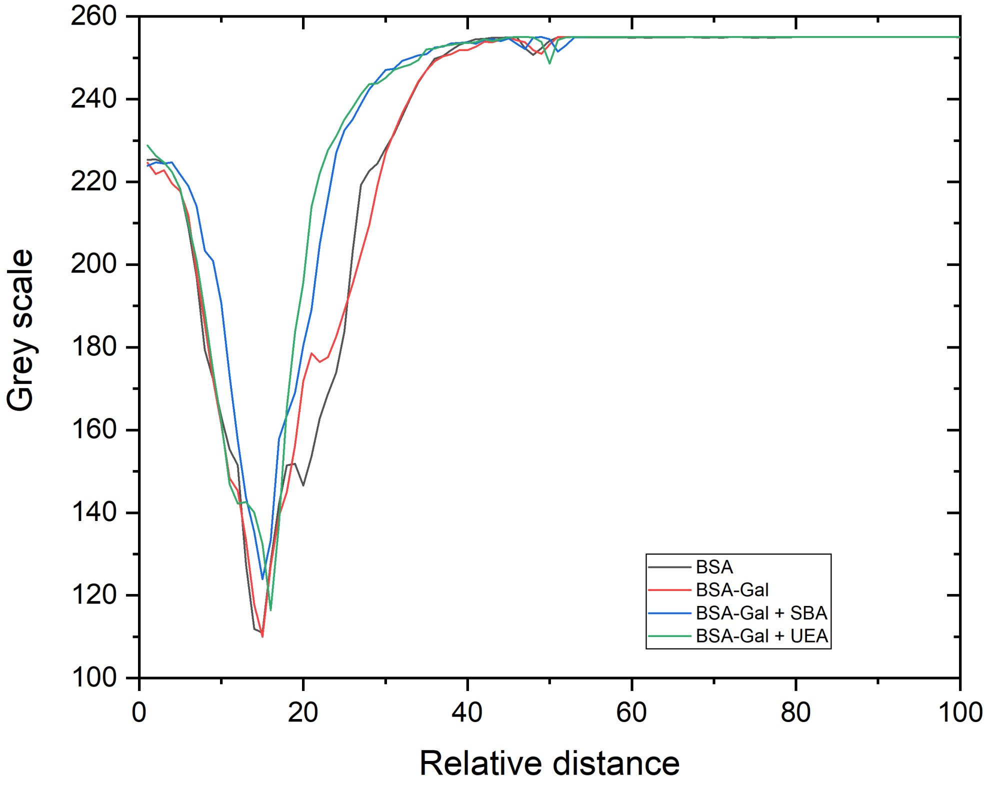

Figure S29. – Analysis of scanned lateral flow strips using 100% GalPHEA$_{40}$@AuNP$_{40}$ particles. Test lines are unfunctionalised BSA (BSA, 1 mg.mL^{-1}), and Galα1-3Galβ1-4GlcNAc-BSA (BSA-Gal, 1 mg.mL^{-1}) with (or without) lectins in solution (SBA or UEA, 0.05 mg.mL^{-1}).

Percentage sugar functionalisation (%)	BSA	BSA-Gal	SBA + BSA-Gal	UEA + BSA-Gal
100	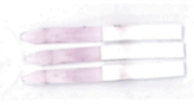	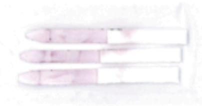	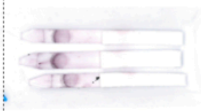	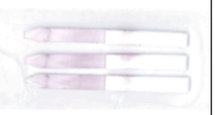
66			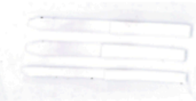	
33	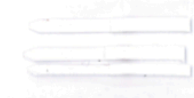		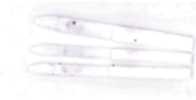	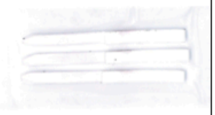
0			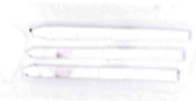	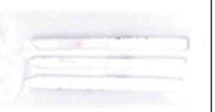

Table S10. – Scans of lateral flow strips using GalPHEA$_{72}$@AuNP$_{40}$ particles of varying glycan functionalisation versus varying test lines of unfunctionalised BSA (BSA, 1 mg.mL^{-1}) and Galα1-3Galβ1-4GlcNAc-BSA (BSA-Gal, 1 mg.mL^{-1}) and with varying lectins (0.05 mg.mL^{-1}).

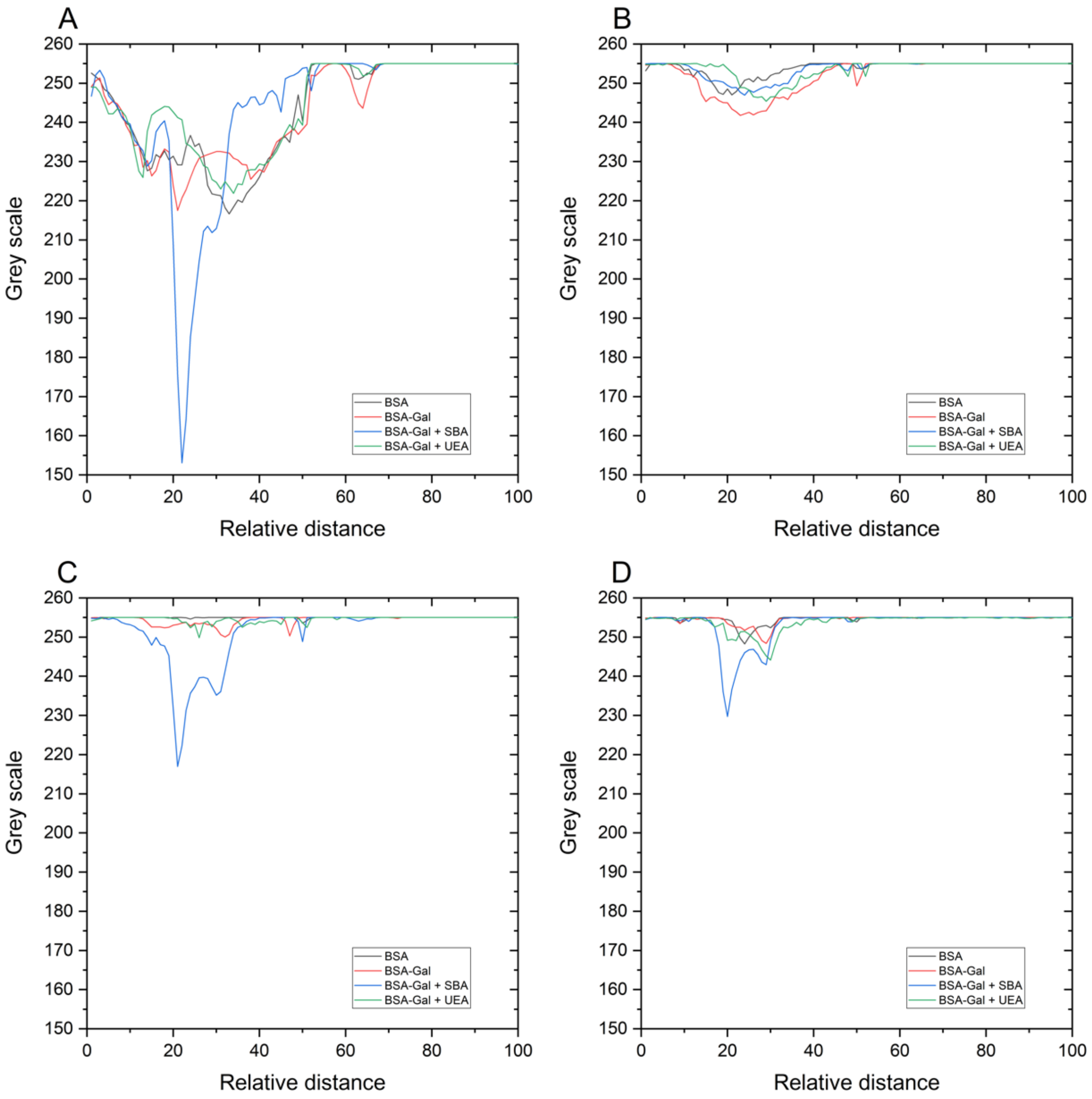

Figure S30. – Analysis of scanned lateral flow strips using GalPHEA$_{72}$@AuNP$_{40}$ particles of varying glycan functionalisation. Test lines are unfunctionalised BSA (BSA, 1 mg.mL^{-1}), and Galα1-3Galβ1-4GlcNAc-BSA (BSA-Gal, 1 mg.mL^{-1}) with (or without) lectins in solution (SBA or UEA, 0.05 mg.mL^{-1}).

Percentage sugar functionalisation (%)	BSA	BSA-Gal	SBA + BSA-Gal	UEA + BSA-Gal
100	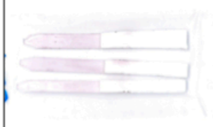	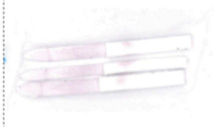	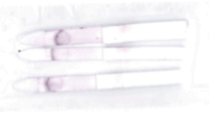	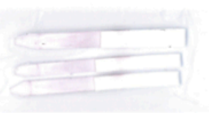
66		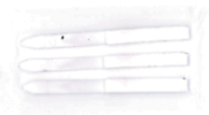	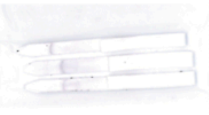	
33			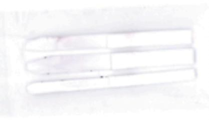	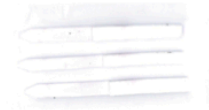
0				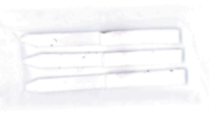

Table S11. – Scans of lateral flow strips using GalPHEA$_{110}$@AuNP$_{40}$ particles of varying glycan functionalisation. Test lines are unfunctionalised BSA (BSA, 1 mg.mL^{-1}), and Galα1-3Galβ1-4GlcNAc-BSA (BSA-Gal, 1 mg.mL^{-1}) with (or without) lectins in solution (SBA or UEA, 0.05 mg.mL^{-1}).

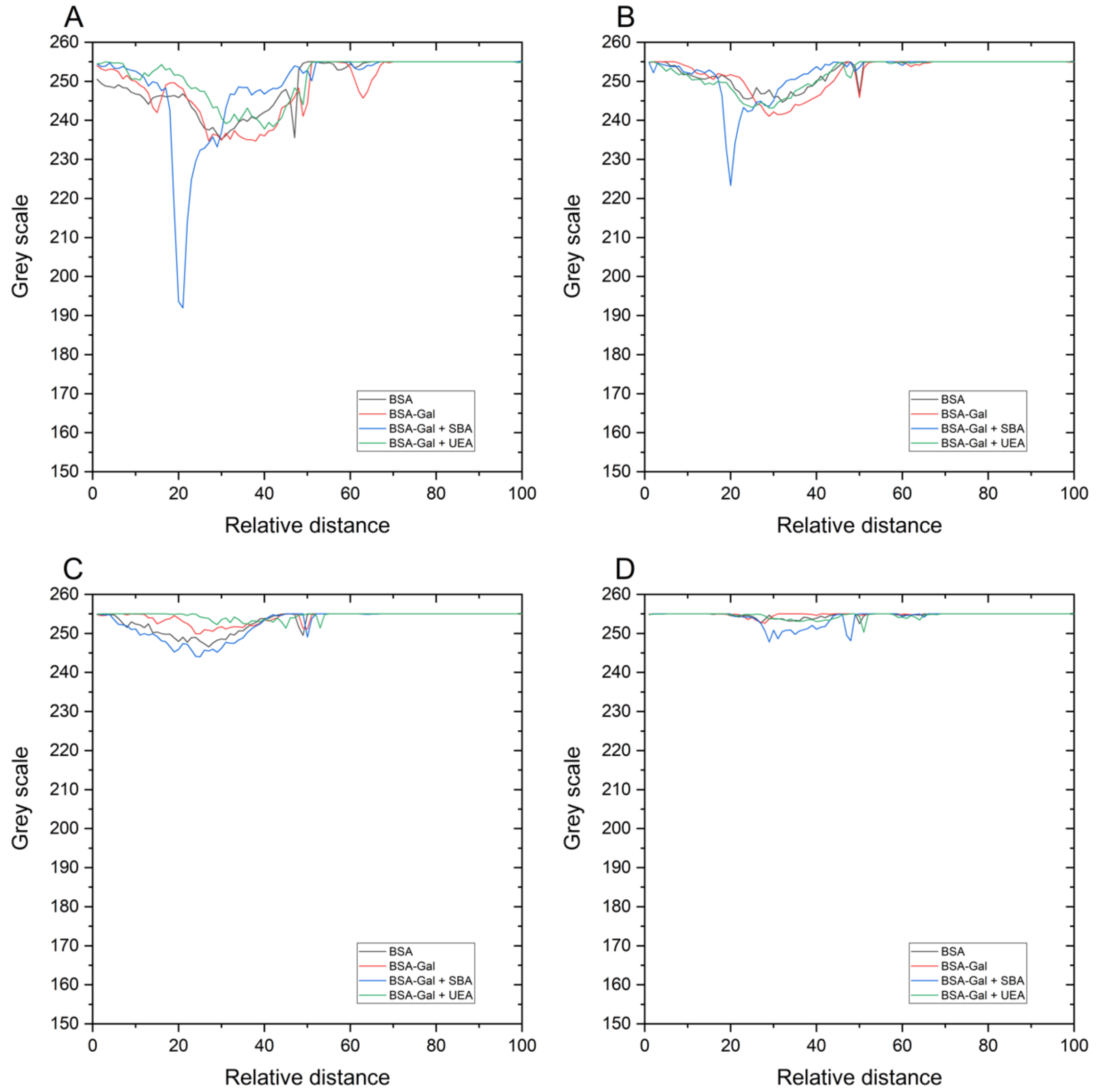

Figure S31. – Analysis of scanned lateral flow strips using GalPHEA$_{110}$@AuNP$_{40}$ particles of varying glycan functionalisation A) 100%, B) 66%, C) 33% and D) 0%. Test lines are unfunctionalised BSA (BSA, 1 mg.mL^{-1}), and Galα1-3Galβ1-4GlcNAc-BSA (BSA-Gal, 1 mg.mL^{-1}) with (or without) lectins in solution (SBA or UEA, 0.05 mg.mL^{-1}).

AuNP System	Signal:Noise
$AuNP_{40}$ vs. BSA	1.6
$AuNP_{40}$ vs. BSA-Gal + SBA	2.8
$AuNP_{40}$ vs. BSA-Gal + UEA	2.8
100% $GalPHEA_{40}$@$AuNP_{40}$ vs. BSA	2.3
100% $GalPHEA_{40}$@$AuNP_{40}$ vs. BSA-Gal + SBA	2.1
100% $GalPHEA_{40}$@$AuNP_{40}$ vs. BSA-Gal + UEA	2.3
100% $GalPHEA_{72}$@$AuNP_{40}$ vs. BSA	1.5
100% $GalPHEA_{72}$@$AuNP_{40}$ vs. BSA-Gal + SBA	3.9
100% $GalPHEA_{72}$@$AuNP_{40}$ vs. BSA-Gal + UEA	1.3
66% $GalPHEA_{72}$@$AuNP_{40}$ vs. BSA	0.8
66% $GalPHEA_{72}$@$AuNP_{40}$ vs. BSA-Gal + SBA	0.8
66% $GalPHEA_{72}$@$AuNP_{40}$ vs. BSA-Gal + UEA	0.9
33% $GalPHEA_{72}$@$AuNP_{40}$ vs. BSA	0.2
33% $GalPHEA_{72}$@$AuNP_{40}$ vs. BSA-Gal + SBA	16.7
33% $GalPHEA_{72}$@$AuNP_{40}$ vs. BSA-Gal + UEA	2.2
0% $GalPHEA_{72}$@$AuNP_{40}$ vs. BSA	3.4
0% $GalPHEA_{72}$@$AuNP_{40}$ vs. BSA-Gal + SBA	12.9
0% $GalPHEA_{72}$@$AuNP_{40}$ vs. BSA-Gal + UEA	5.6
100% $GalPHEA_{110}$@$AuNP_{40}$ vs. BSA	1.5
100% $GalPHEA_{110}$@$AuNP_{40}$ vs. BSA-Gal + SBA	4.7
100% $GalPHEA_{110}$@$AuNP_{40}$ vs. BSA-Gal + UEA	1.2
66% $GalPHEA_{110}$@$AuNP_{40}$ vs. BSA	1.2
66% $GalPHEA_{110}$@$AuNP_{40}$ vs. BSA-Gal + SBA	3.8

66% $GalPHEA_{110}$@$AuNP_{40}$ vs. BSA-Gal + UEA	1.4
33% $GalPHEA_{110}$@$AuNP_{40}$ vs. BSA	2.8
33% $GalPHEA_{110}$@$AuNP_{40}$ vs. BSA-Gal + SBA	3.6
33% $GalPHEA_{110}$@$AuNP_{40}$ vs. BSA-Gal + UEA	0.9
0% $GalPHEA_{110}$@$AuNP_{40}$ vs. BSA	4.4
0% $GalPHEA_{110}$@$AuNP_{40}$ vs. BSA-Gal + SBA	13.5
0% $GalPHEA_{110}$@$AuNP_{40}$ vs. BSA-Gal + UEA	3.6

Table S12. – Signal to noise ratios of all AuNP40 systems

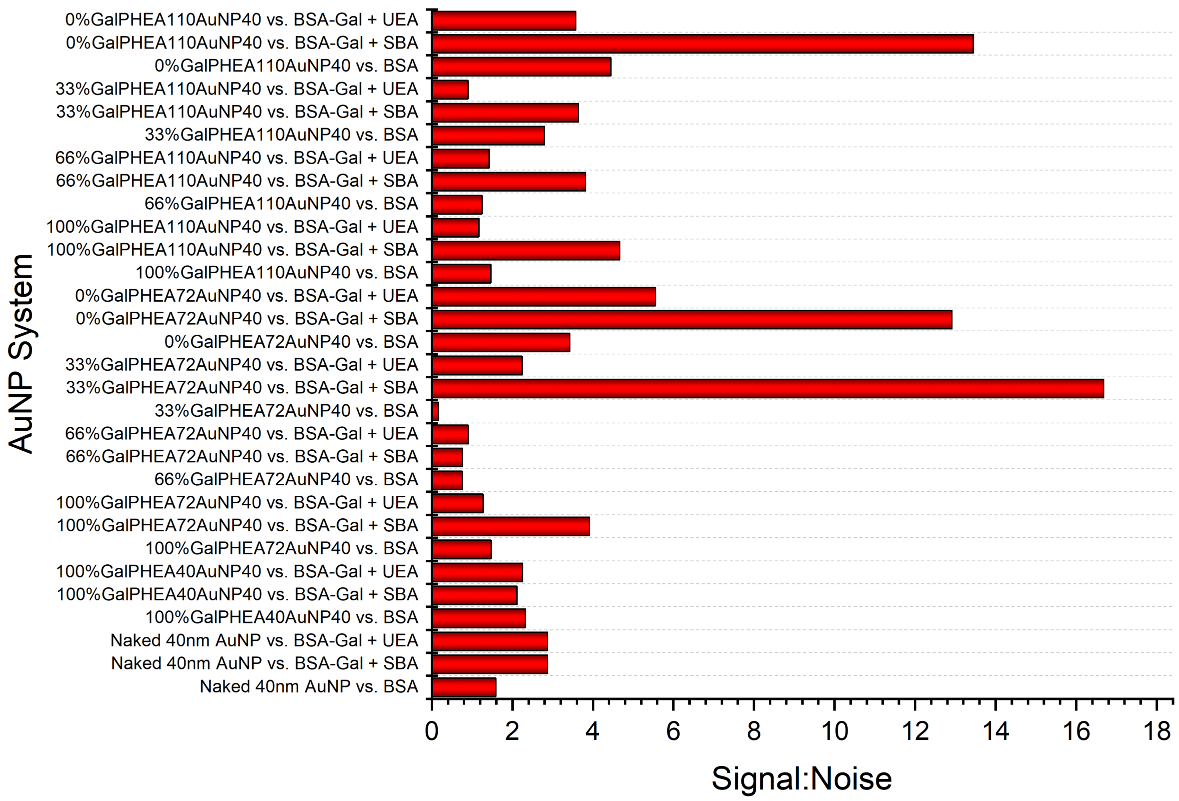

Figure S32. – Signal to noise ratios of all 40nm AuNPs

Concentration of SBA, mg.ml^{-1}	SBA + BSA-Gal
0.05	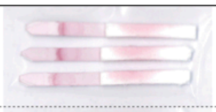
0.04	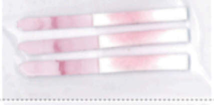
0.03	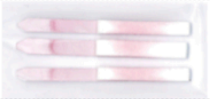
0.025	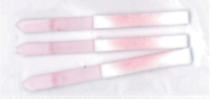
0.02	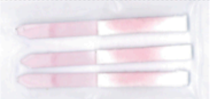
0.01	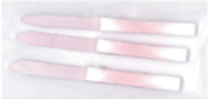
0.0075	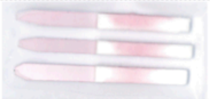
0.005	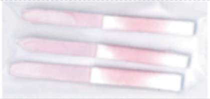

Table S13. – Scans of lateral flow strips using 100% GalPHEA$_{72}$@AuNP$_{16}$. Test lines of 1 mg.mL^{-1} Galα1-3Galβ1-4GlcNAc-BSA (BSA-Gal) with varying SBA concentrations in solution.

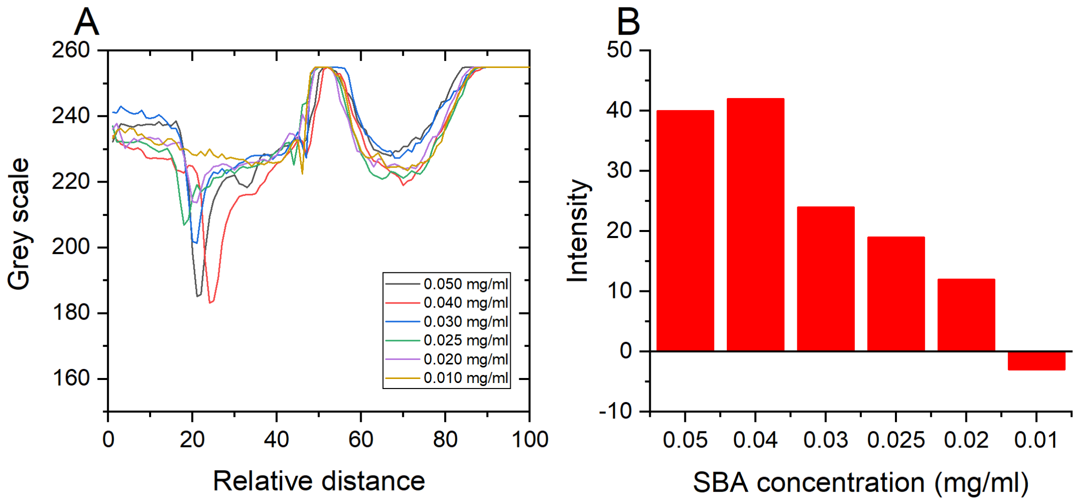

Figure S33. – Analysis of scanned lateral flow strips using 100% GalPHEA$_{72}$@AuNP$_{16}$. Test line of 1 mg.mL^{-1} Galα1-3Galβ1-4GlcNAc-BSA with varying SBA concentrations in solution. A) Analysed data plotted and B) Intensity

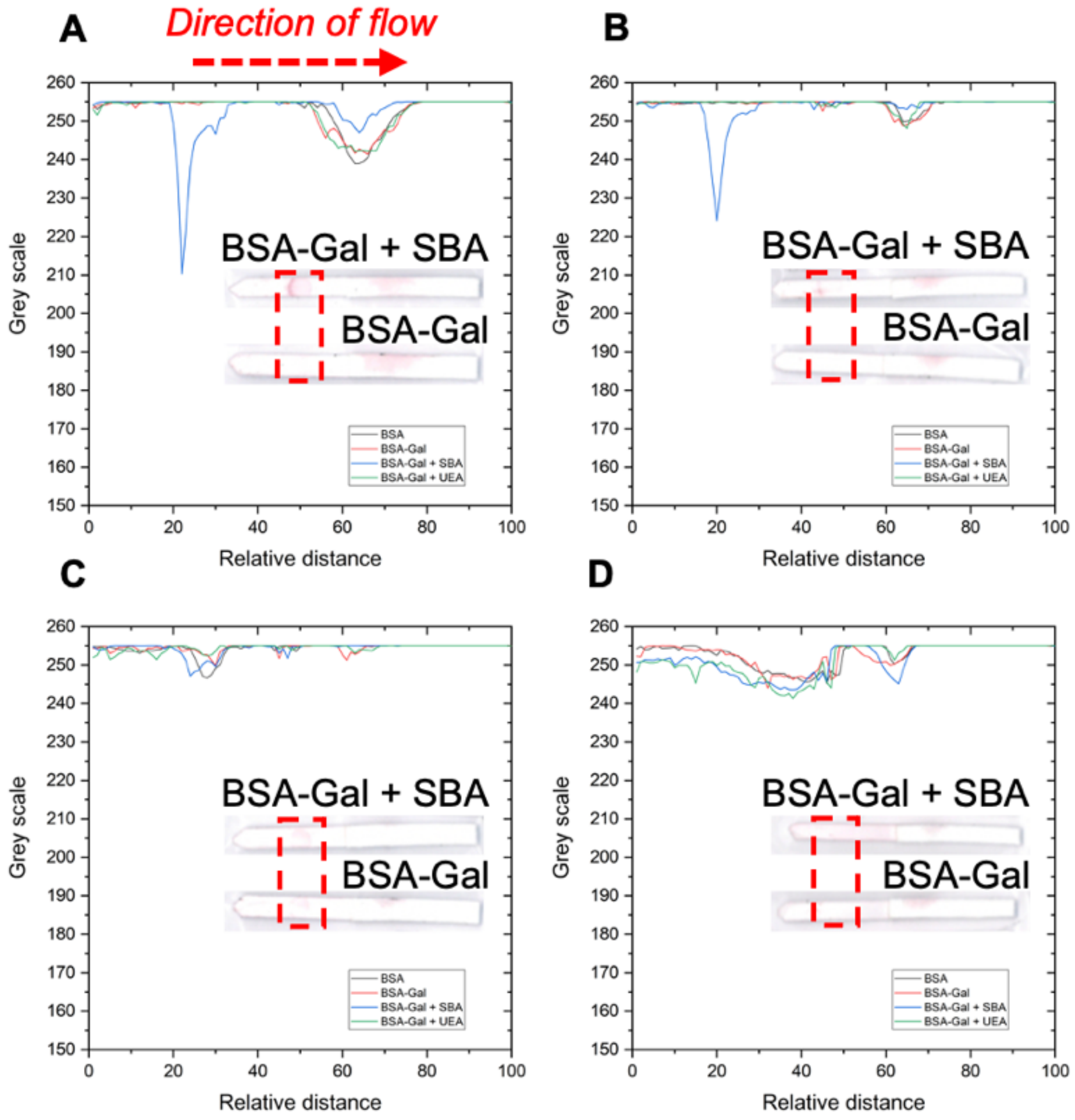

Figure S34A. – Lateral flow dipsticks versus SBA as the analyte including image analysis results and example photographs. A) 100% GalPHEA$_{72}$@AuNP$_{16}$ B) 66% GalPHEA$_{72}$@AuNP$_{16}$ C) 33% GalPHEA$_{72}$@AuNP$_{16}$ D) 0% GalPHEA$_{72}$@AuNP$_{16}$ E) 100% GalPHEA$_{40}$@AuNP$_{16}$ F) 100% GalPHEA$_{110}$@AuNP$_{16}$ G) 100% GalPHEA$_{72}$@AuNP$_{40}$ H) Signal to noise analysis of select AuNP systems. [BSA – unglycosylated BSA (1 mg.mL^{-1}), BSA-Gal Galα1-3Galβ1-4GlcNAc-BSA (1 mg.mL^{-1}), SBA & UEA (0.05 mg.mL^{-1})].

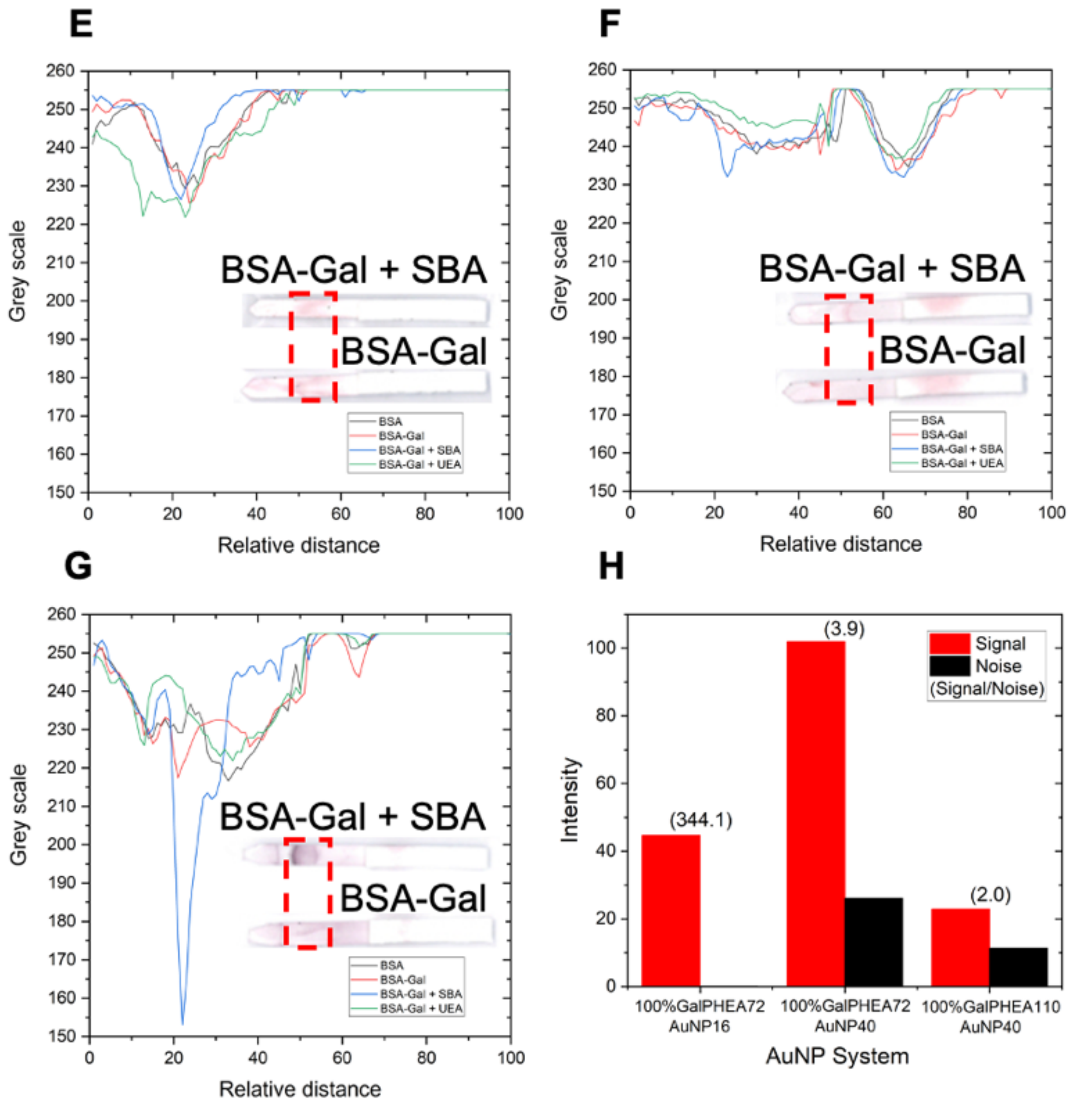

Figure S34B. – Lateral flow dipsticks versus SBA as the analyte including image analysis results and example photographs. A) 100% GalPHEA$_{72}$@AuNP$_{16}$ B) 66% GalPHEA$_{72}$@AuNP$_{16}$ C) 33% GalPHEA$_{72}$@AuNP$_{16}$ D) 0% GalPHEA$_{72}$@AuNP$_{16}$ E) 100% GalPHEA$_{40}$@AuNP$_{16}$ F) 100% GalPHEA$_{110}$@AuNP$_{16}$ G) 100% GalPHEA$_{72}$@AuNP$_{40}$ H) Signal to noise analysis of select AuNP systems. [BSA – unglycosylated BSA (1 mg.mL^{-1}), BSA-Gal Galα1-3Galβ1-4GlcNAc-BSA (1 mg.mL^{-1}), SBA & UEA (0.05 mg.mL^{-1})].

Lateral Flow Cassette Data

Concentration of SBA, mg.ml^{-1}	Cassettes	Strips
0.1	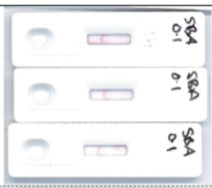	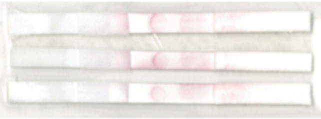
0.05	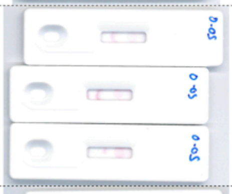	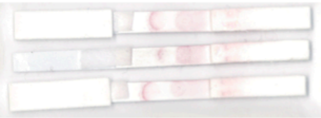
0.04	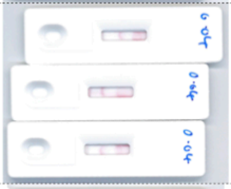	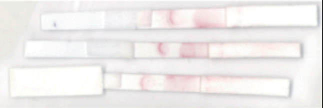
0.03	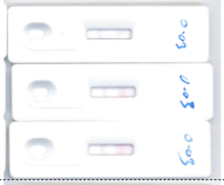	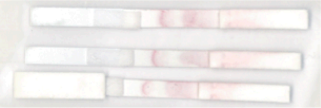
0.02	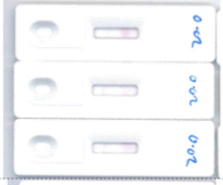	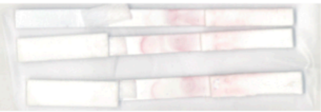
0.01	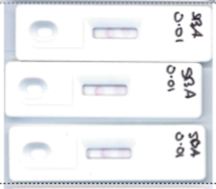	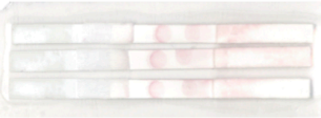
0.005	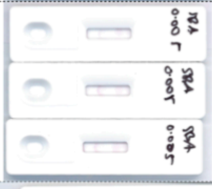	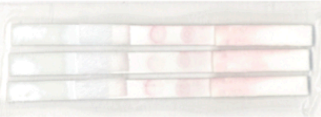
0.0005	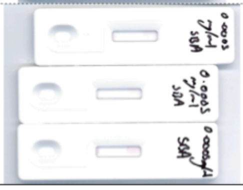	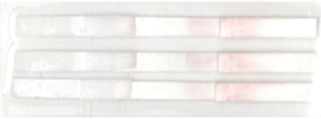

Table S14. – Scans of lateral flow cassettes and strips from the cassettes using 100% $GalPHEA_{72}@AuNP_{16}$. Test line of 1 mg.mL^{-1} Galα1-3Galβ1-4GlcNAc-BSA with varying SBA concentrations in solution.

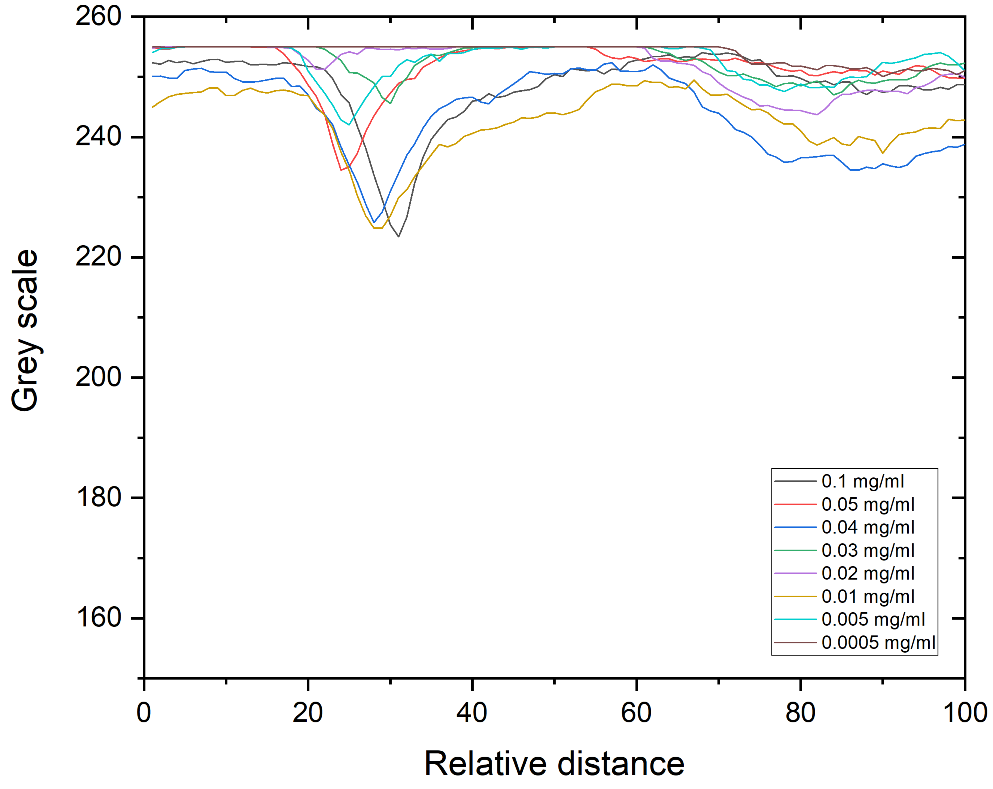

Figure S35. – Analysis of scanned lateral flow cassettes using 100% GalPHEA$_{72}$@AuNP$_{16}$. Test line of 1 mg.mL^{-1} Galα1-3Galβ1-4GlcNAc-BSA with varying SBA concentrations in solution.

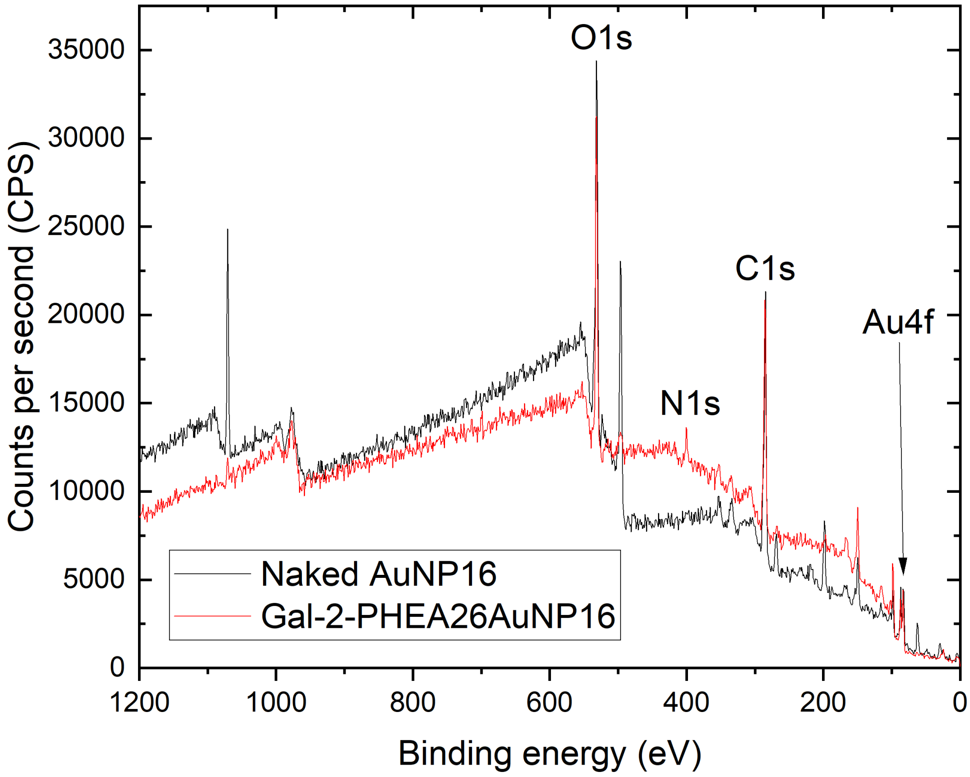

Figure S36. – Representative XPS survey scans of $AuNP_{16}$ (Naked AuNP16) and 100% $GalPHEA_{26}@AuNP_{16}$ (Gal-2-PHEA26AuNP16)

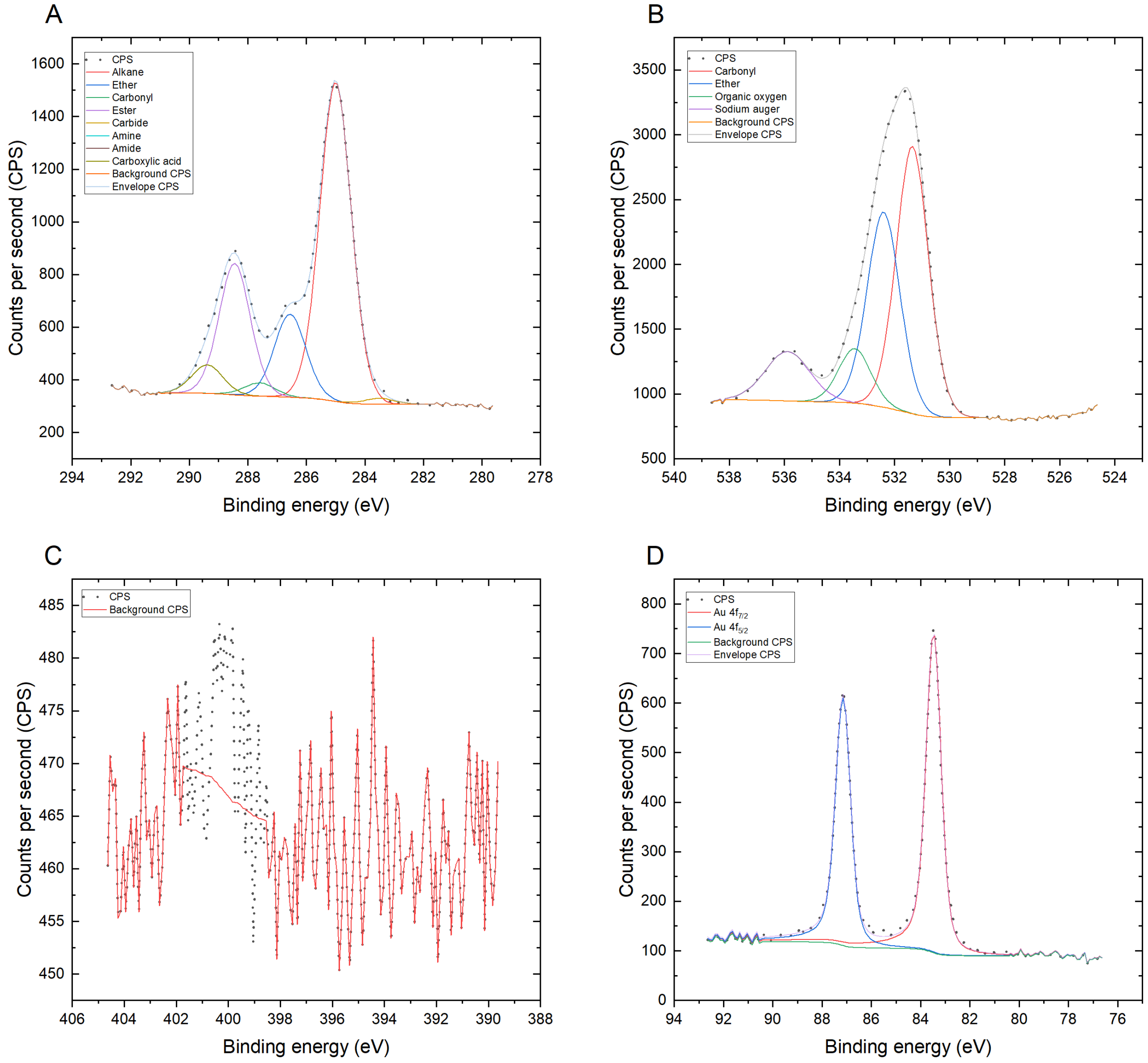

Figure S37. – XPS of $AuNP_{16}$ A) C 1*s* B) O 1*s* C) N 1*s* and D) Au 4*f*

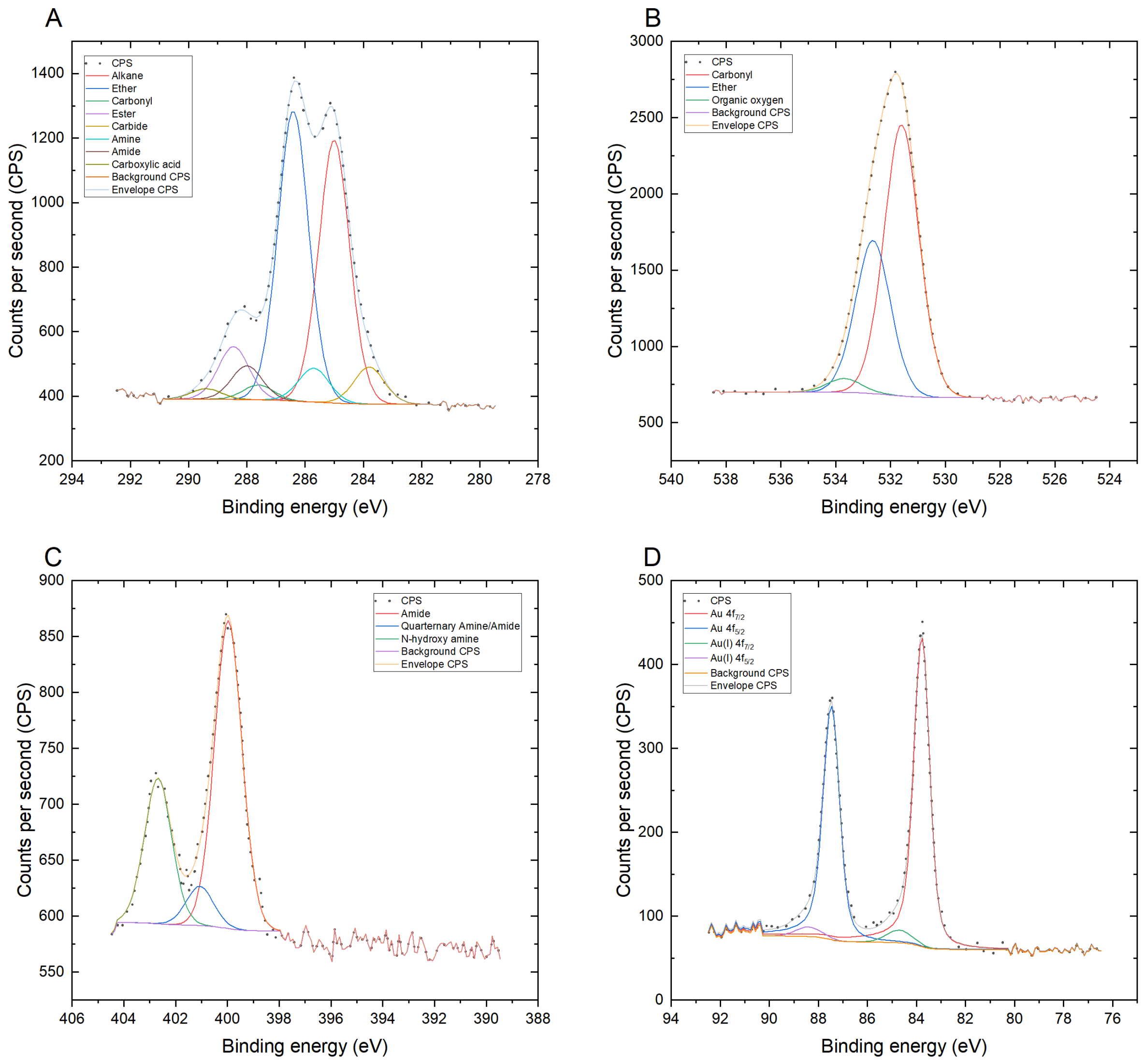

Figure S38. – XPS of 100% GalPHEA$_{26}$@AuNP$_{16}$ A) C 1*s* B) O 1*s* C) N 1*s* and D) Au 4*f*

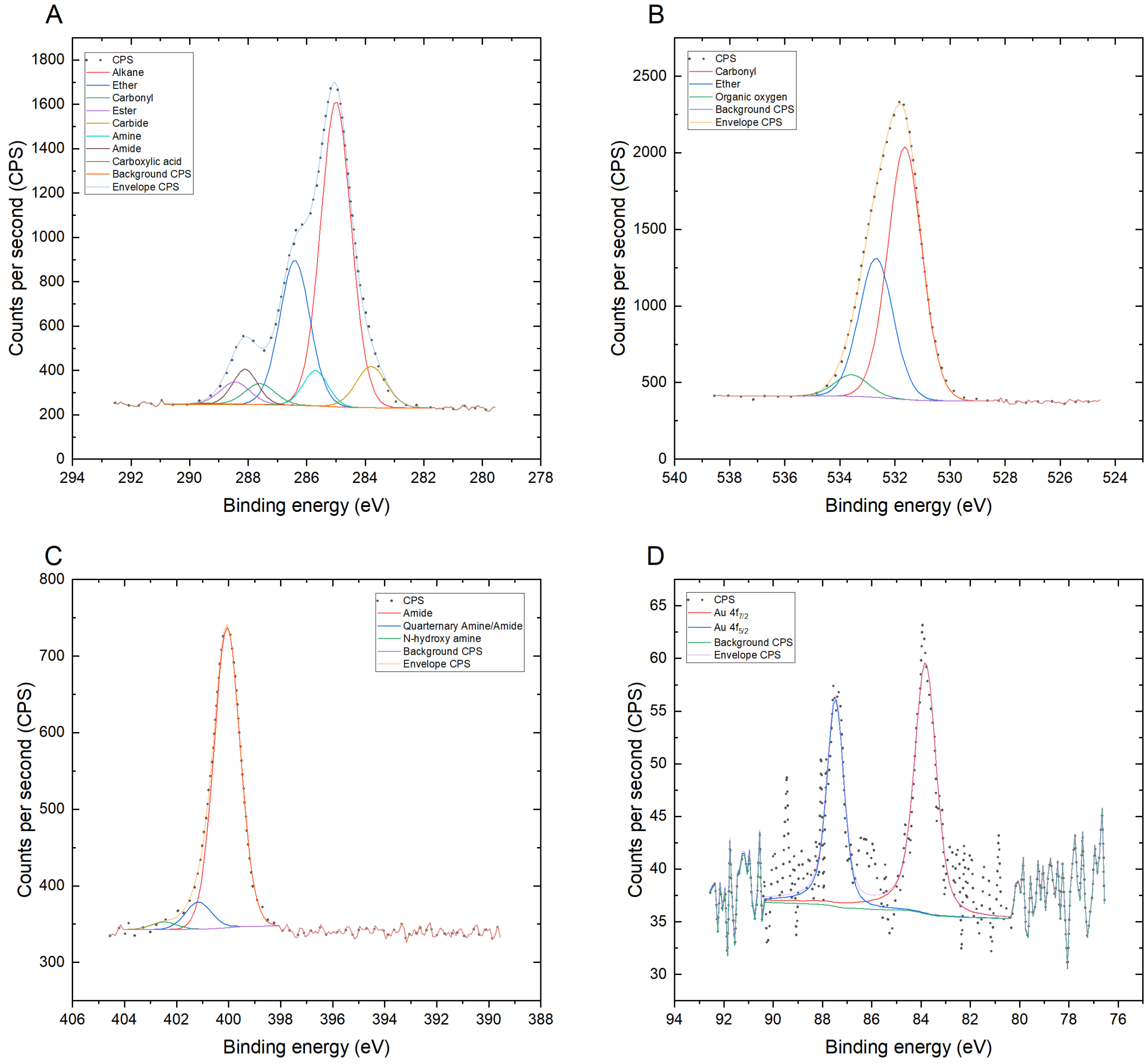

Figure S39. – XPS of 100% GalPHEA$_{40}$@AuNP$_{16}$ A) C 1*s* B) O 1*s* C) N 1*s* and D) Au 4*f*

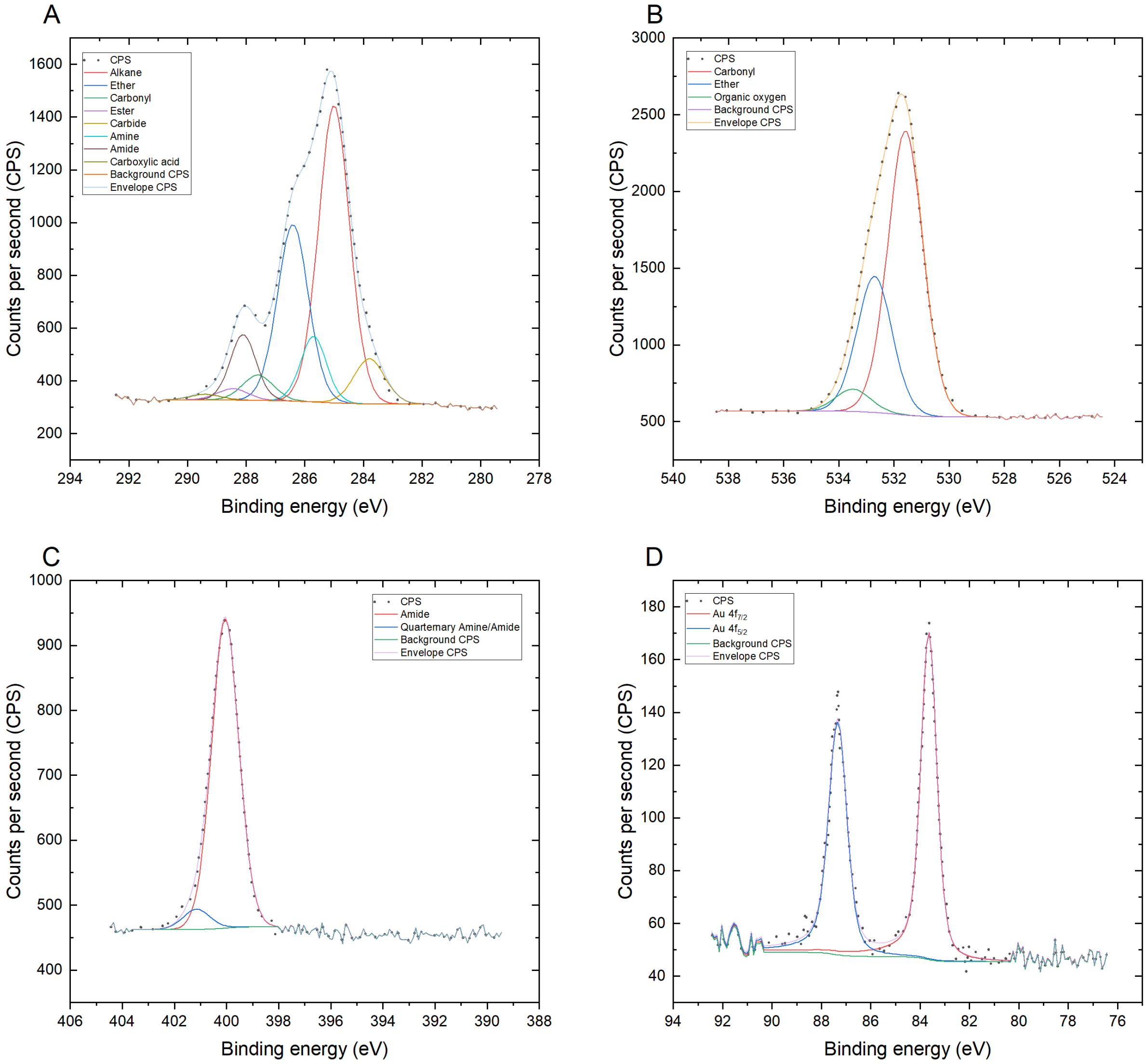

Figure S40. – XPS of 100% GalPHEA$_{72}$@AuNP$_{16}$ A) C 1*s* B) O 1*s* C) N 1*s* and D) Au 4*f*

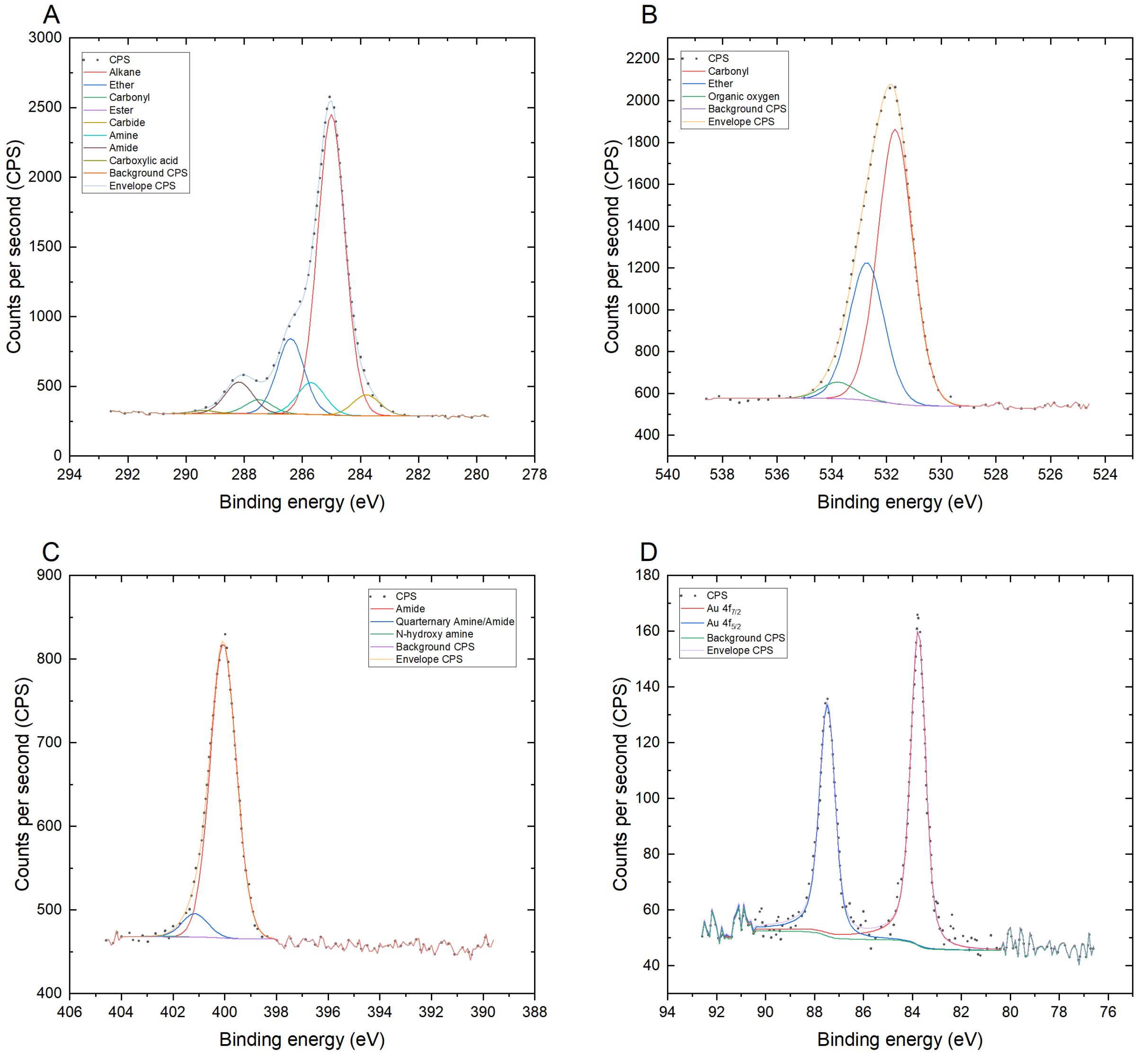

Figure S41. – XPS of 100% GalPHEA$_{110}$@AuNP$_{16}$ A) C 1*s* B) O 1*s* C) N 1*s* and D) Au 4*f*

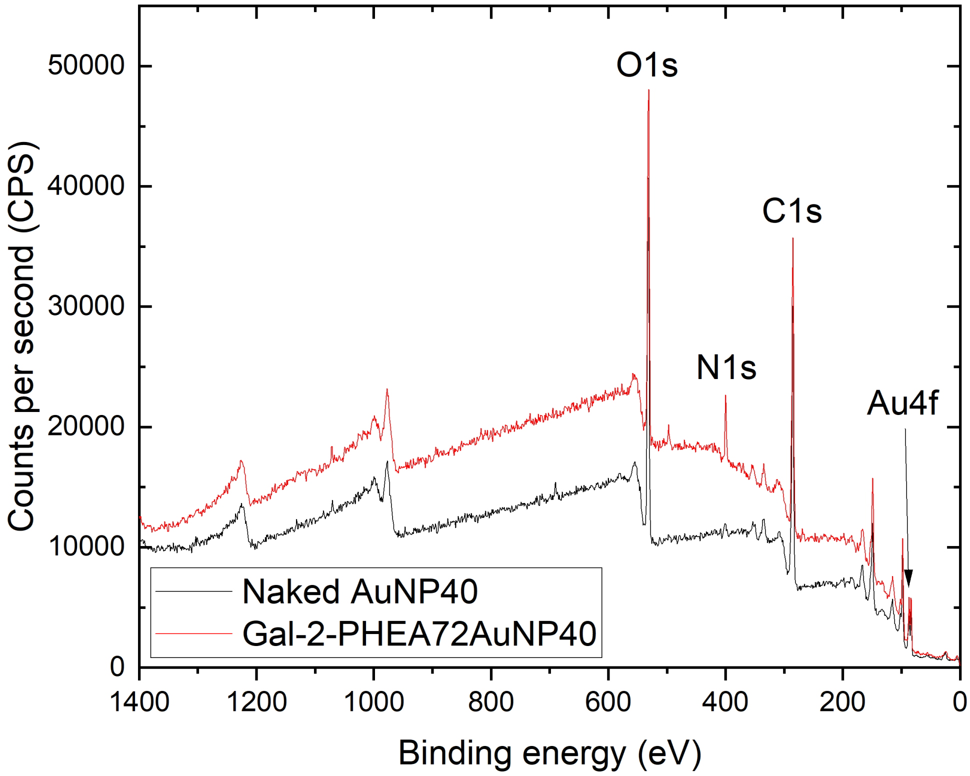

Figure S42. – Representative XPS survey scans of $AuNP_{40}$ (Naked AuNP40) and $GalPHEA_{72}@AuNP_{40}$ (Gal-2-PHEA72AuNP40)

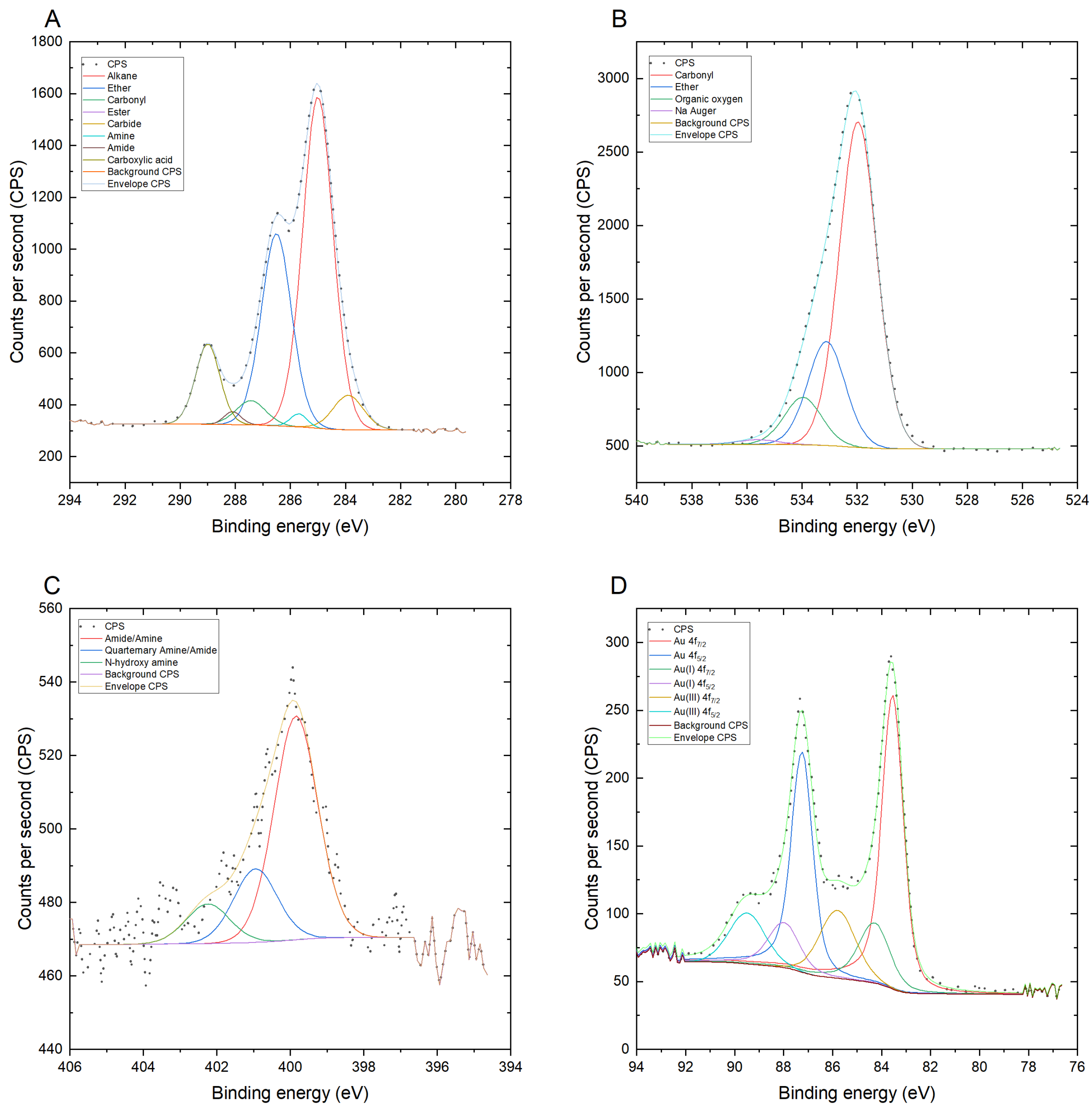

Figure S43. – XPS of $AuNP_{40}$ A) C 1*s* B) O 1*s* C) N 1*s* and D) Au 4*f*

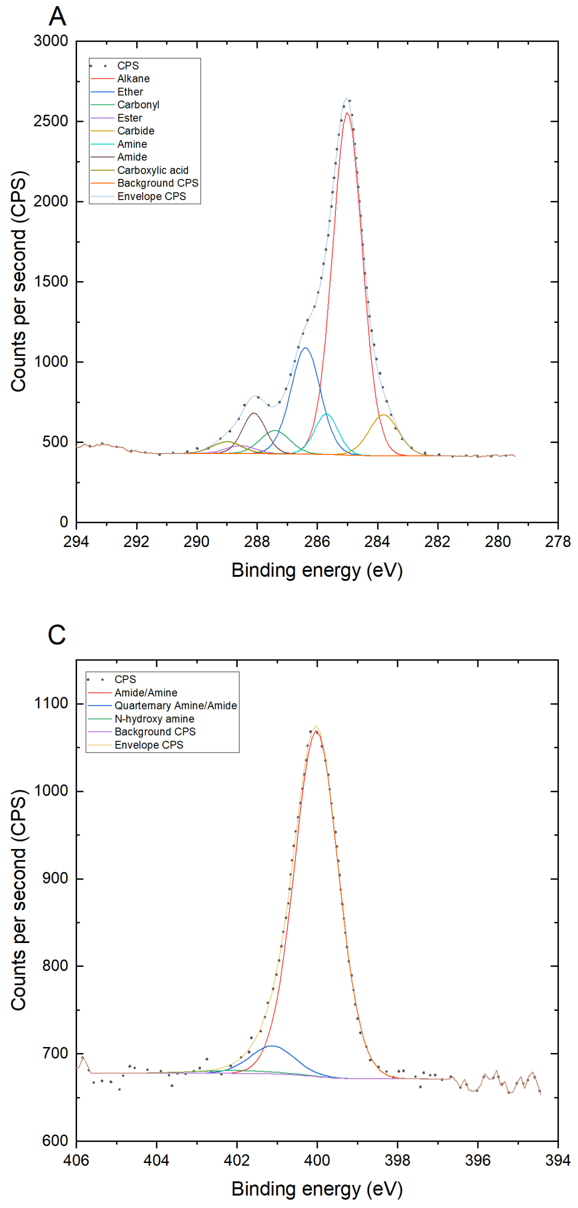

Figure S44. – XPS of 100% GalPHEA$_{40}$@AuNP$_{40}$ A) C 1*s* B) O 1*s* C) N 1*s* and D) Au 4*f*

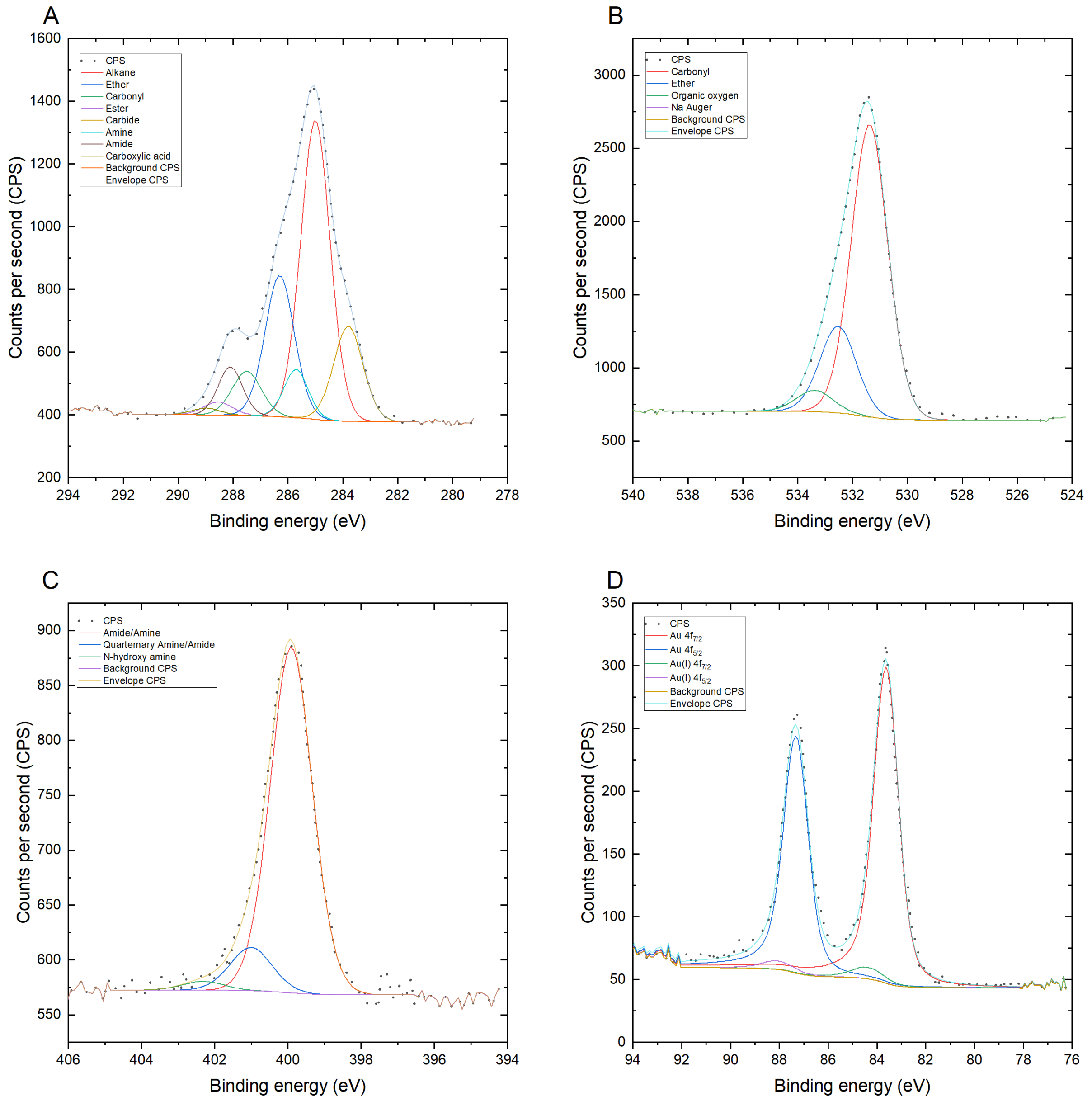

Figure S45. – XPS of 100% GalPHEA$_{72}$@AuNP$_{40}$ A) C 1*s* B) O 1*s* C) N 1*s* and D) Au 4*f*

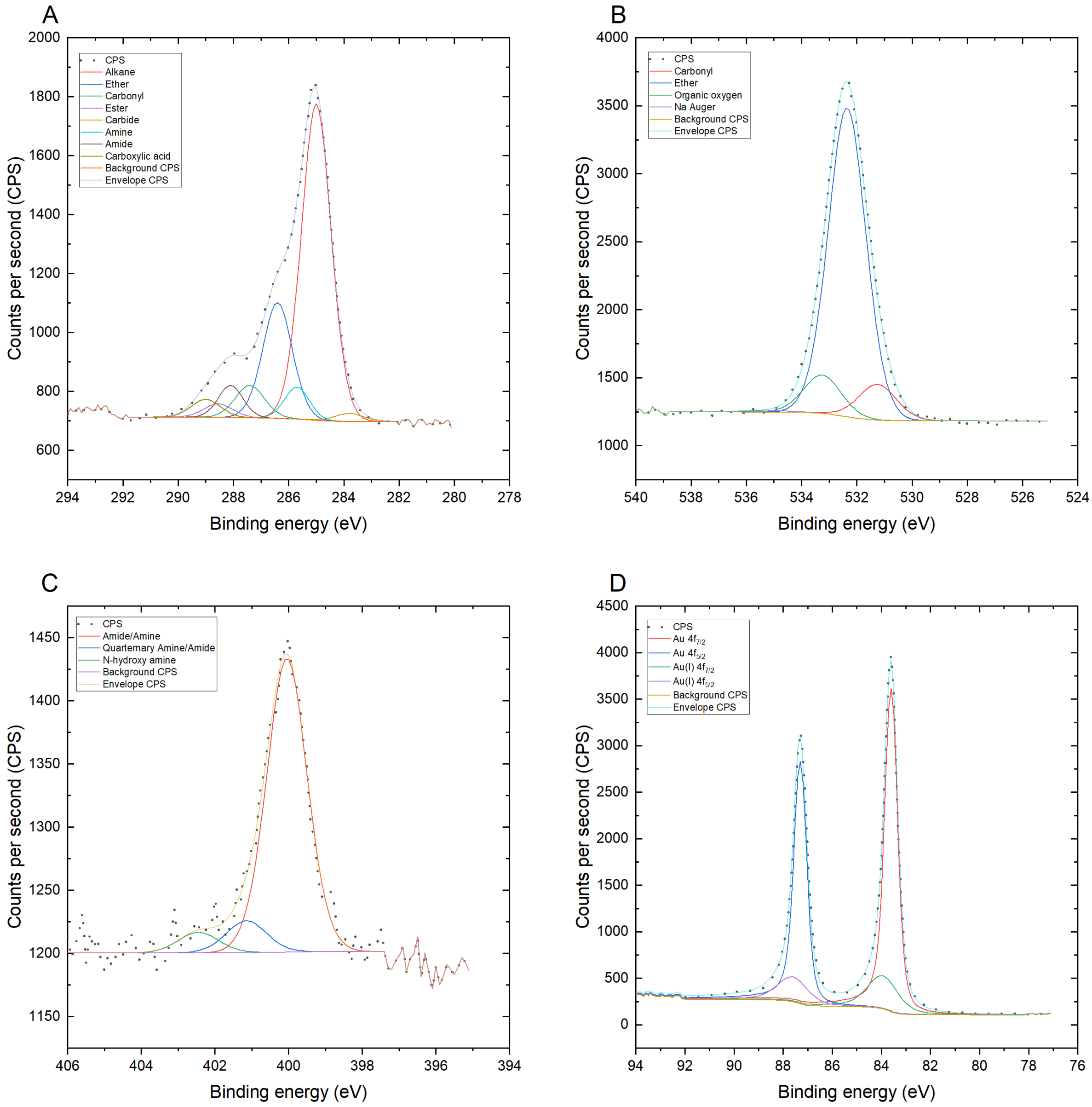

Figure S46. – XPS of 100% GalPHEA$_{110}$@AuNP$_{40}$ A) C 1*s* B) O 1*s* C) N 1*s* and D) Au 4*f*

Particle Composition		Elemental Percentage Composition (%)				Elemental Ratios	
AuNP (nm)	PHEA DP	C 1*s*	O 1*s*	N 1*s*	Au 4*f*	N 1*s*/C 1*s*	N 1*s*/Au 4*f*
16	0	53.04	45.81	0.14	1.01	0.003	0.138
16	26	58.45	34.28	6.61	0.67	0.113	9.926
16	40	64.11	29.80	6.03	0.06	0.094	106.817
16	72	60.50	32.20	7.10	0.20	0.117	34.707
16	110	72.19	22.55	5.09	0.17	0.070	29.320
40	0	60.48	37.27	1.50	0.75	0.025	2.000
40	40	67.17	26.97	5.16	0.70	0.077	7.371
40	72	57.70	35.26	6.27	0.77	0.109	8.143
40	110	51.12	37.51	4.66	6.71	0.091	0.694

Table S15. – Elemental composition of 2-deoxy-2-amino-galactose functionalised nanoparticles determined by XPS

Particle Composition		C 1*s* Bond Percentage Composition (%)								Bond Ratios	
AuNP (nm)	PHEA DP	Alkane	Ether	Carbonyl	Ester	Carbide	Amine	Amide	Carboxylic Acid	Amide/Alkane	Amide/Ether
16	0	54.83	14.31	2.31	22.61	1.02	0.00	0.00	4.93	0.0000	0.0000
16	26	35.66	39.38	2.04	7.21	5.03	4.61	4.62	1.45	0.1296	0.1173
16	40	51.35	24.38	3.57	3.79	6.98	4.87	4.88	0.18	0.0950	0.2002
16	72	44.27	26.30	3.80	1.72	6.73	8.15	8.16	0.86	0.1843	0.3103
16	110	62.47	15.71	2.99	0.15	4.39	6.81	6.82	0.67	0.1092	0.4341
40	0	50.03	28.99	3.67	0.00	5.23	1.12	1.12	9.85	0.0224	0.0386
40	40	57.26	17.83	3.94	1.35	6.88	5.36	5.36	2.02	0.0936	0.3006
40	72	43.93	20.75	6.53	1.93	13.98	5.93	5.93	1.02	0.1350	0.2858
40	110	56.64	20.74	5.81	2.43	1.46	4.85	4.86	3.22	0.0858	0.2343

Table S16. – C 1*s* bonding composition of 2-deoxy-2-amino-galactose functionalised nanoparticles determined by XPS

RCA120-Targeting AuNP Data

DLS and UV-vis Data

Table S17. – Nanoparticles Synthesised and Characterisation

**Particle**	**Generation[a]**	**λ_{SPR}[b] (nm)**	**$\lambda_{SPR}/\lambda_{450}$[b] (nm)**	**DLS[c] (nm)**
$AuNP_{16}$	1	518	1.56	20.69 ±0.8
$GalPHEA_{40}$@$AuNP_{16}$	1	525	1.52	17.40 ±2.1
$GalPHEA_{50}$@$AuNP_{16}$	1	523	1.54	17.96 ±0.3
$GalPHEA_{58}$@$AuNP_{16}$	1	522	1.55	18.07 ±1.5
$GalPHEA_{72}$@$AuNP_{16}$	1	525	1.56	19.75 ±1.1
$GalPHEA_{110}$@$AuNP_{16}$	1	524	1.57	31.58 ±2.0

[a]Generation of nanoparticles used from the seeding synthetic methodology. [b]Maximum absorption wavelength from the surface plasmon resonance band of the particles and characteristic ratio. [c]Diameter from dynamic light scattering ± standard error from three measurements.

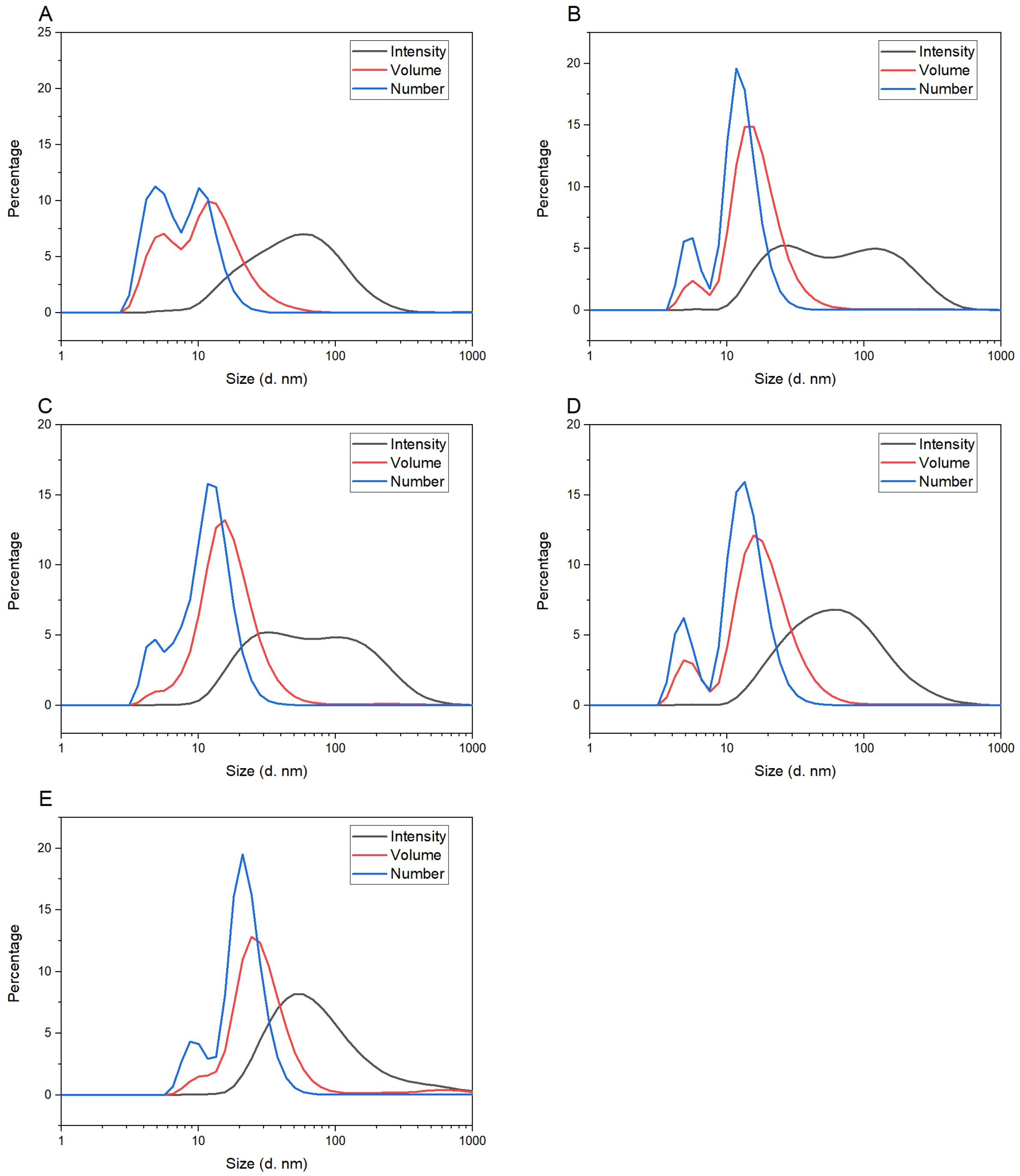

Figure S47. – DLS data for 16nm AuNPs by degree of polymerisation A) 40 B) 50 C) 58 D) 72 E) 110

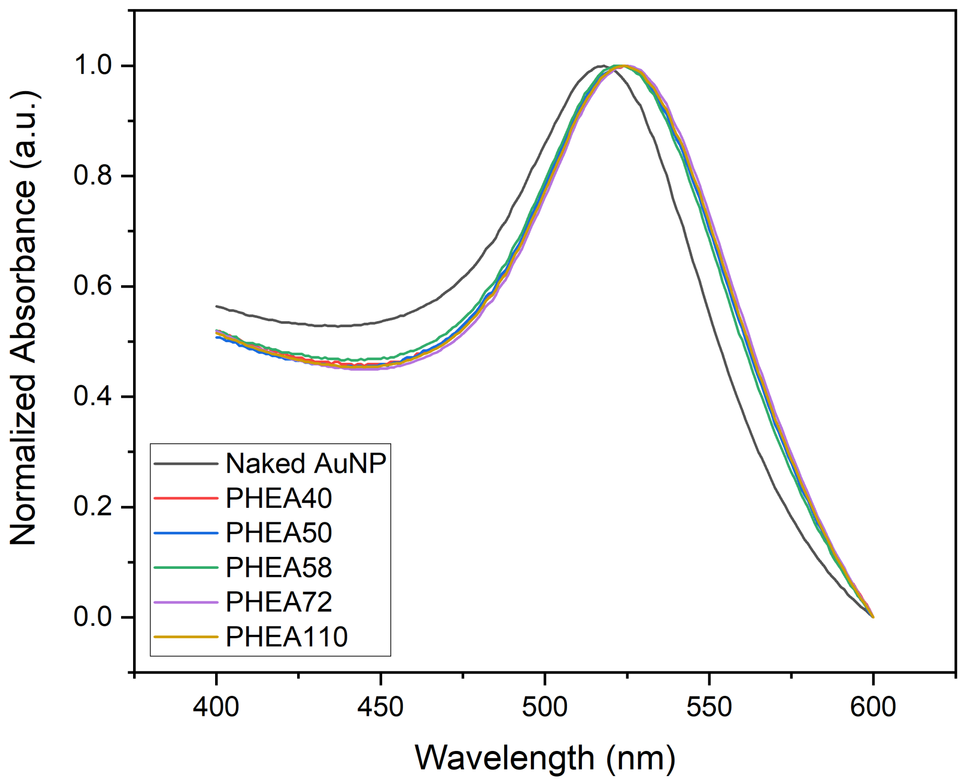

Figure S48. – UV-vis for 16nm AuNPs by degree of polymerisation

Flow-Through Strip Data

AuNP System	No Lectin	5 mg.ml^{-1} RCA_{120}	5 mg.ml^{-1} SBA	5 mg.ml^{-1} WGA	BSA-Gal
Gal-1-PHEA40AuNP 16	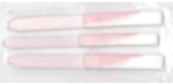	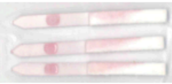	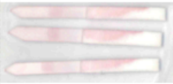	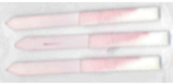	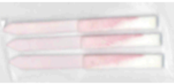
Gal-1-PHEA72AuNP 16	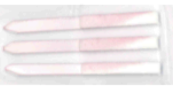	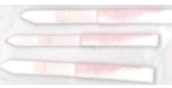	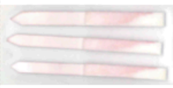	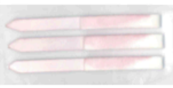	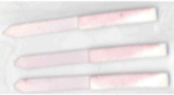
Gal-1-PHEA110AuNP 16	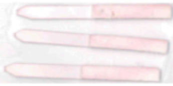			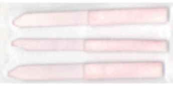	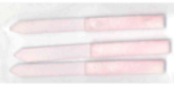
Gal-2-PHEA72AuNP 16		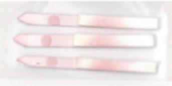	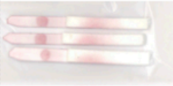		

AuNP System	No Lectin	5 mg.ml^{-1} RCA_{120}	5 mg.ml^{-1} SBA	5 mg.ml^{-1} WGA	BSA-Gal
Gal-1-PHEA50AuNP 16	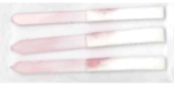	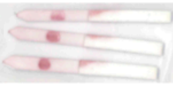	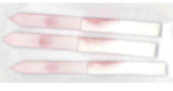	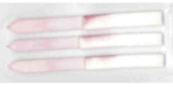	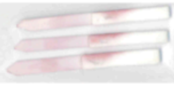
Gal-1-PHEA58AuNP 16	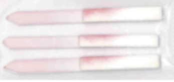	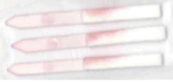	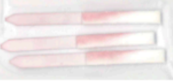	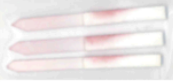	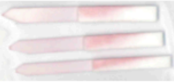

Table S18. – Scans of flow-through strips. Tests were done using Gal-1-$PHEA_X$@$AuNP_{16}$ (1-deoxy-1-amino-galactose functionalised) particles and 100% Gal-2-$PHEA_{72}$@$AuNP_{16}$ (2-deoxy-2-amino-galactose functionalised) particles. Test lines of 1 mg.mL^{-1} Galα1-3Galβ1-4GlcNAc-BSA (BSA-Gal) and varying lectins (RCA_{120}, SBA and WGA) of 5 mg.mL^{-1}

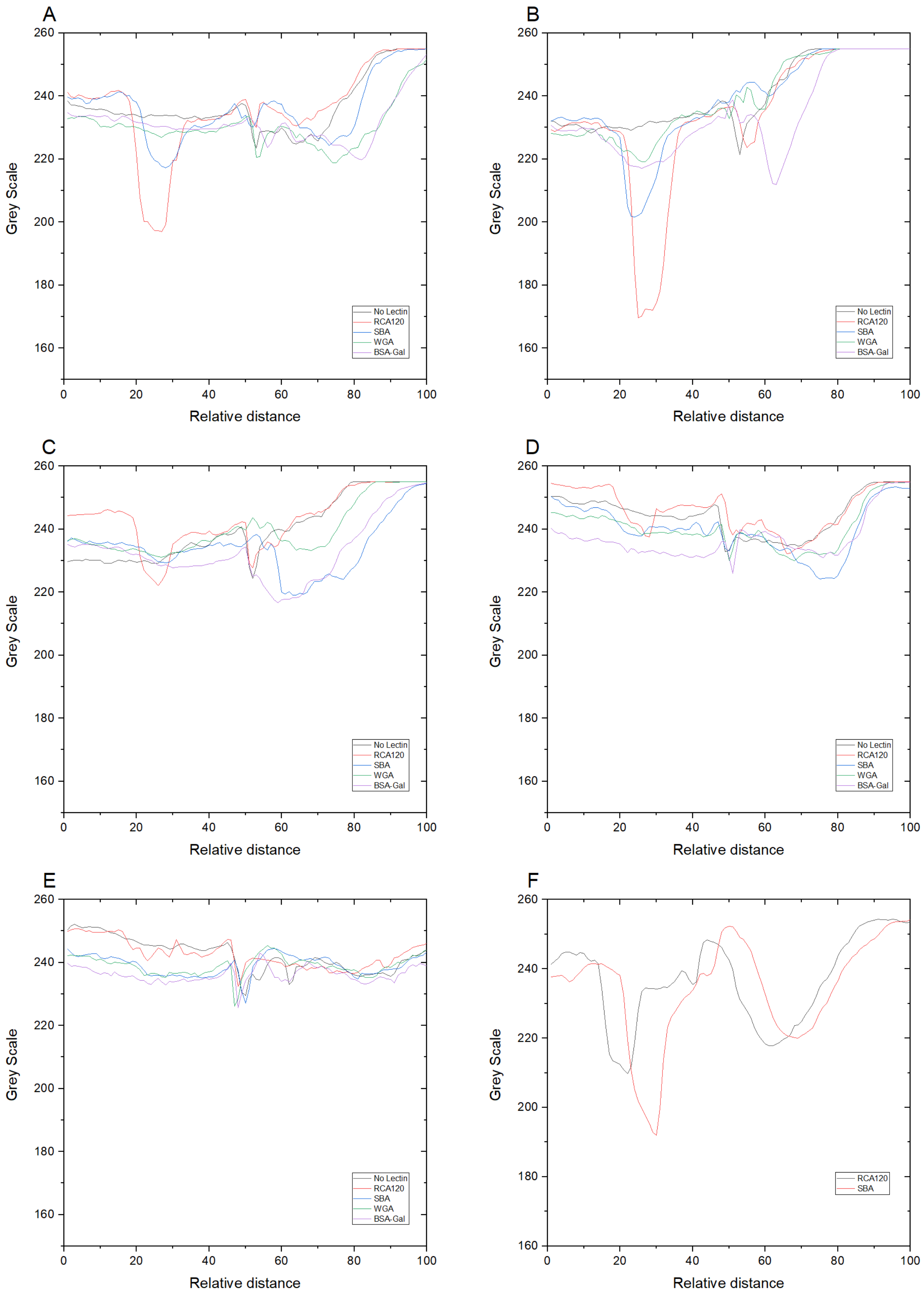

Figure S49. – Analysis of scanned flow-through strips. Tests were done using Gal-1-PHEA$_X$@AuNP$_{16}$ (1-deoxy-1-amino-galactose functionalised) particles (A) 40 B) 50 C) 58 D) 72 E) 110) and 100% Gal-2-PHEA$_{72}$@AuNP$_{16}$ (2-deoxy-2-amino-galactose functionalised)

particles (F). Test lines of 1 mg.mL^{-1} Galα1-3Galβ1-4GlcNAc-BSA (BSA-Gal) and varying lectins (RCA_{120}, SBA and WGA) of 5 mg.mL^{-1}. Degree of polymerization varies as follows A) 40 B) 50 C) 58 D) 72 E) 110 and F) 100% Gal-2-$PHEA_{72}$@$AuNP_{16}$

	RCA120	SBA	WGA
**5 mg.ml^{-1}**	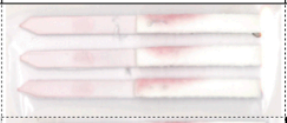	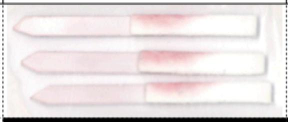	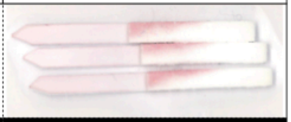
**4 mg.ml^{-1}**	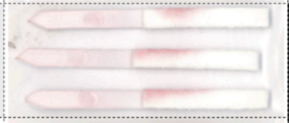		
**3 mg.ml^{-1}**	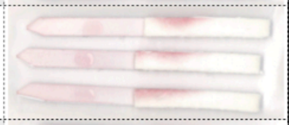		
**2 mg.ml^{-1}**	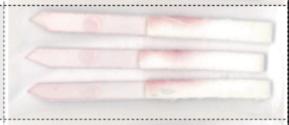		
**1 mg.ml^{-1}**	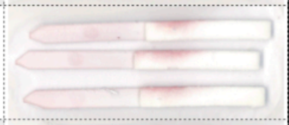		
**0.5 mg.ml^{-1}**	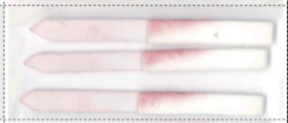		
**0.1 mg.ml^{-1}**	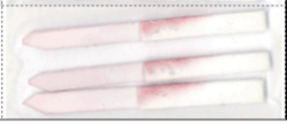		

Table S19. – Scans of flow-through strips. Tests were done using $GalPHEA_{58}$@$AuNP_{16}$ particles. Test lines of differing RCA_{120} (RCA) concentrations or, 5 mg.mL^{-1} SBA or WGA.

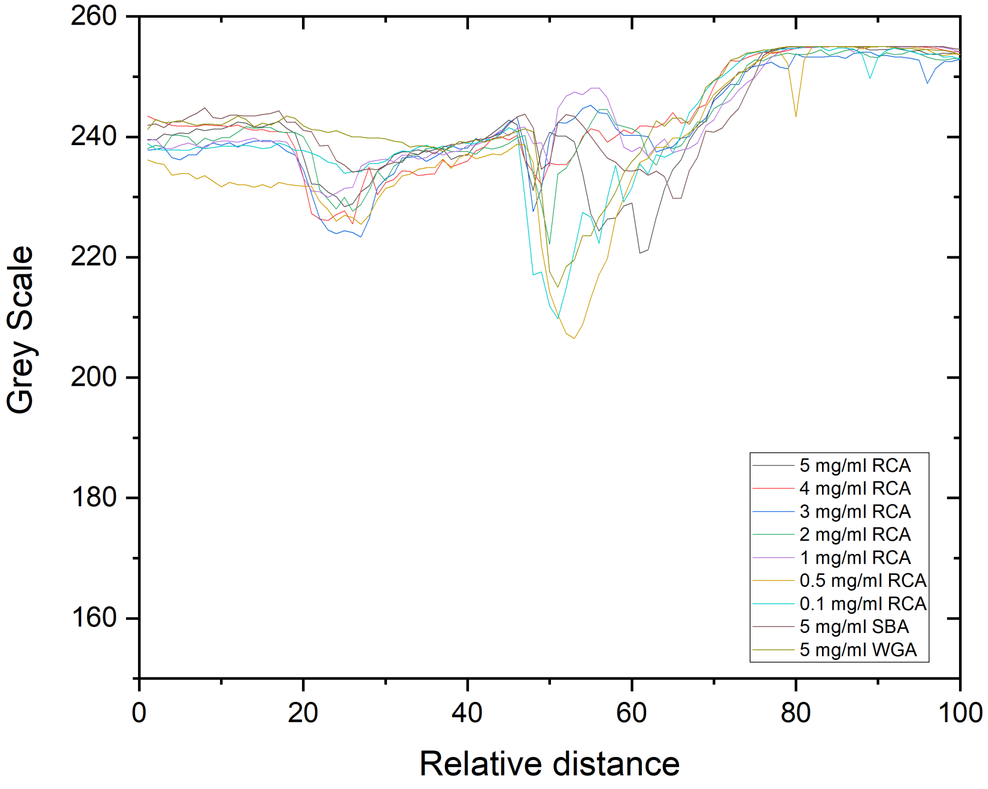

Figure S50. – Analysis of scanned flow-through strips. Tests were done using GalPHEA$_{58}$@AuNP$_{16}$ particles. Test lines of differing RCA$_{120}$ (RCA) concentrations or, 5 mg.mL^{-1} SBA or WGA.

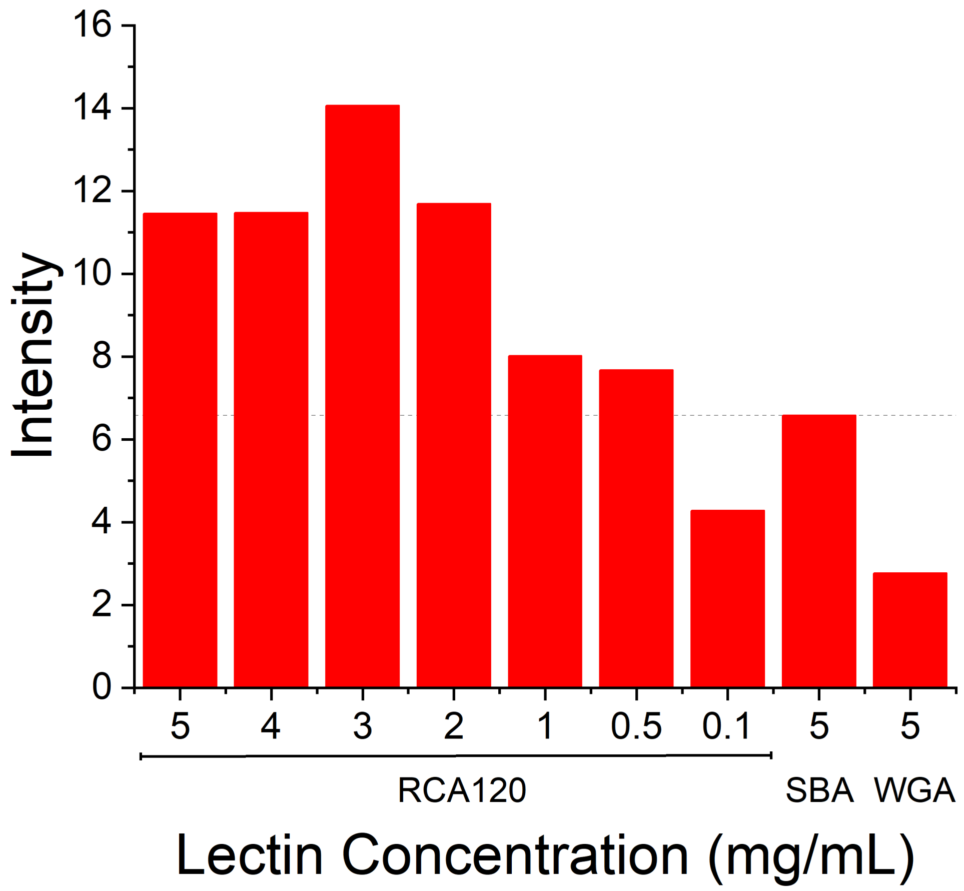

Figure S51. – Signal intensity analysis of scanned flow-through strips. Tests were done using $GalPHEA_{58}@AuNP_{16}$ particles. Test lines of differing RCA_{120} (RCA) concentrations or, 5 $mg.mL^{-1}$ SBA or WGA.

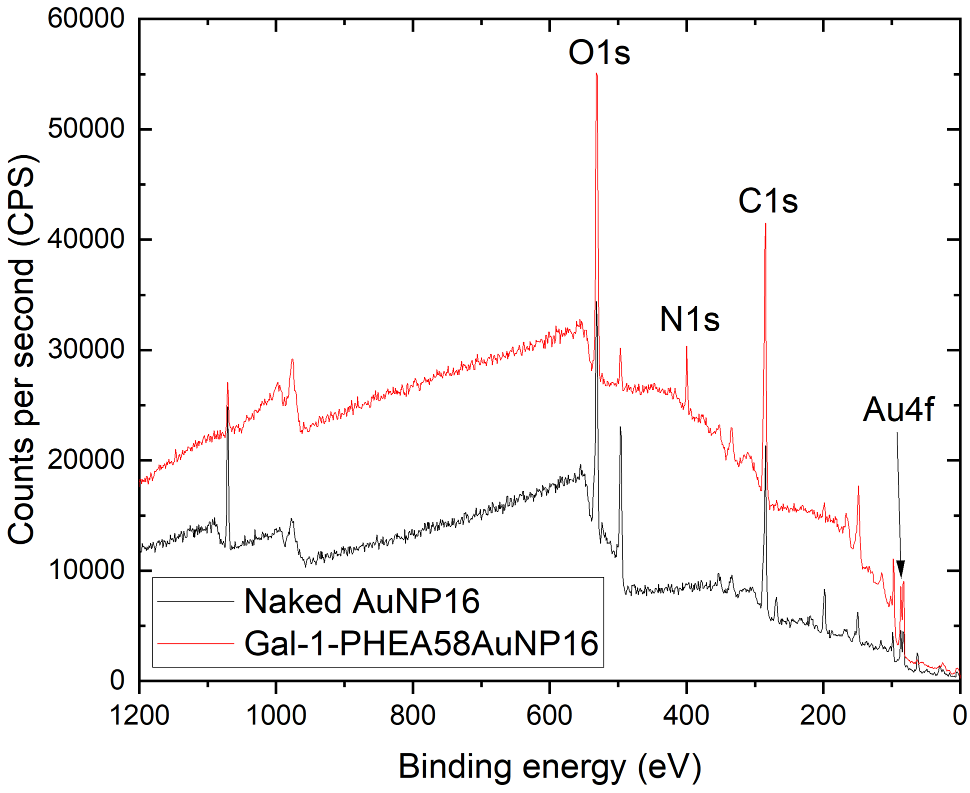

Figure S52. – Representative XPS survey scans of $AuNP_{16}$ (Naked AuNP16) and $GalPHEA_x@AuNP_{16}$ (Gal-1-PHEA58AuNP16)

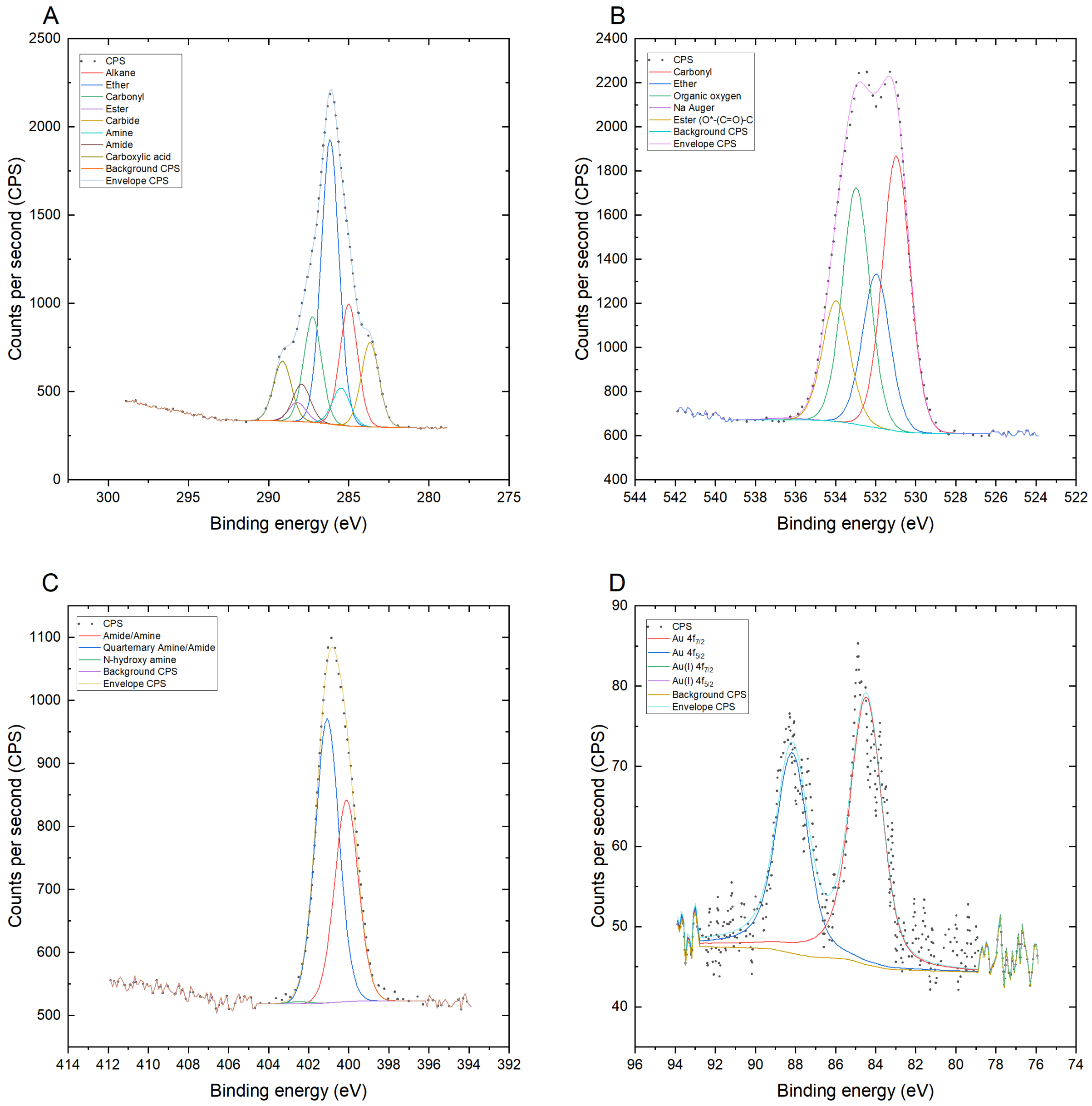

Figure S53. – XPS of GalPHEA$_{40}$@AuNP$_{16}$ A) C 1*s* B) O 1*s* C) N 1*s* and D) Au 4*f*

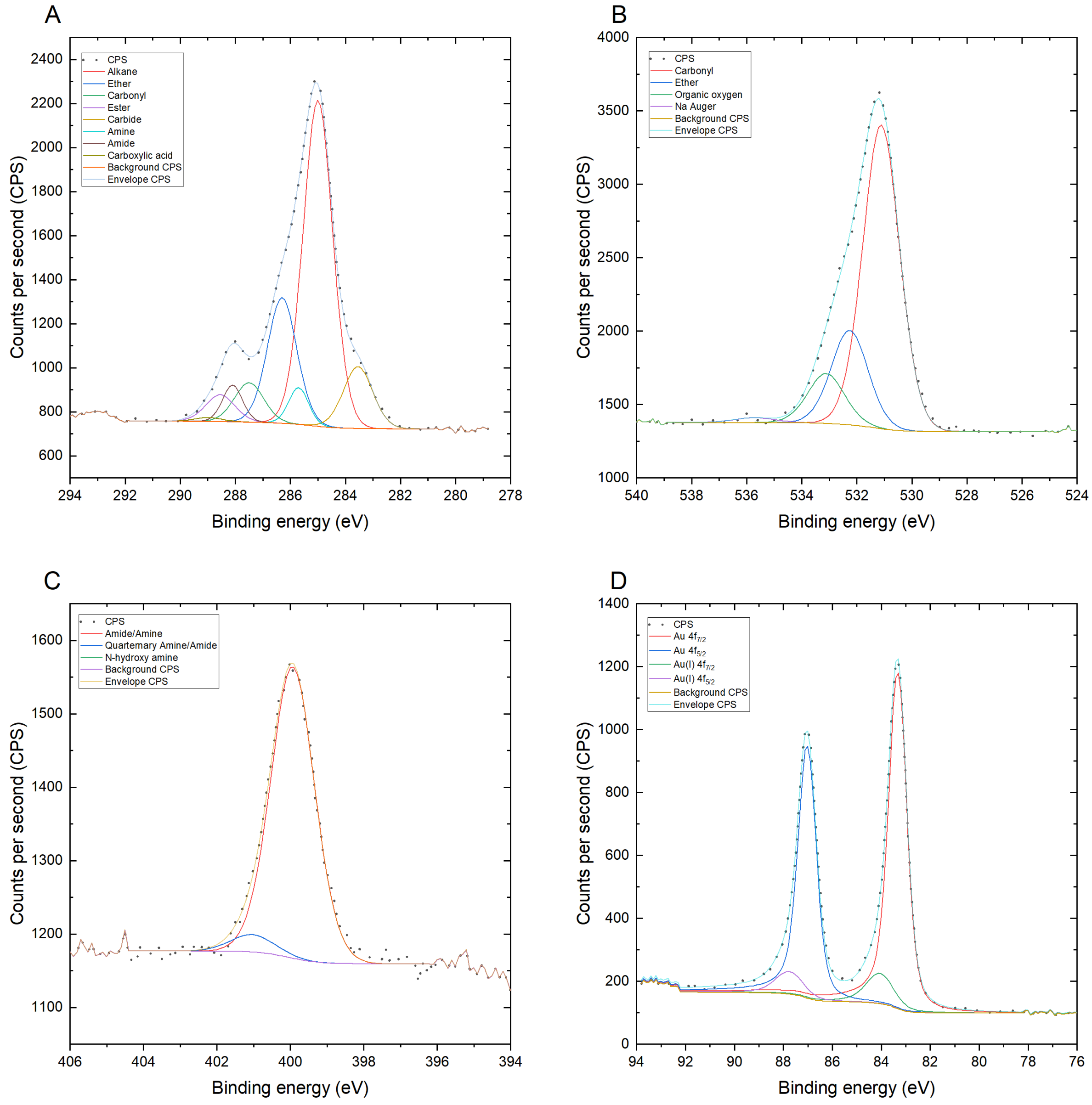

Figure S54. – XPS of GalPHEA$_{50}$@AuNP$_{16}$ A) C 1*s* B) O 1*s* C) N 1*s* and D) Au 4*f*

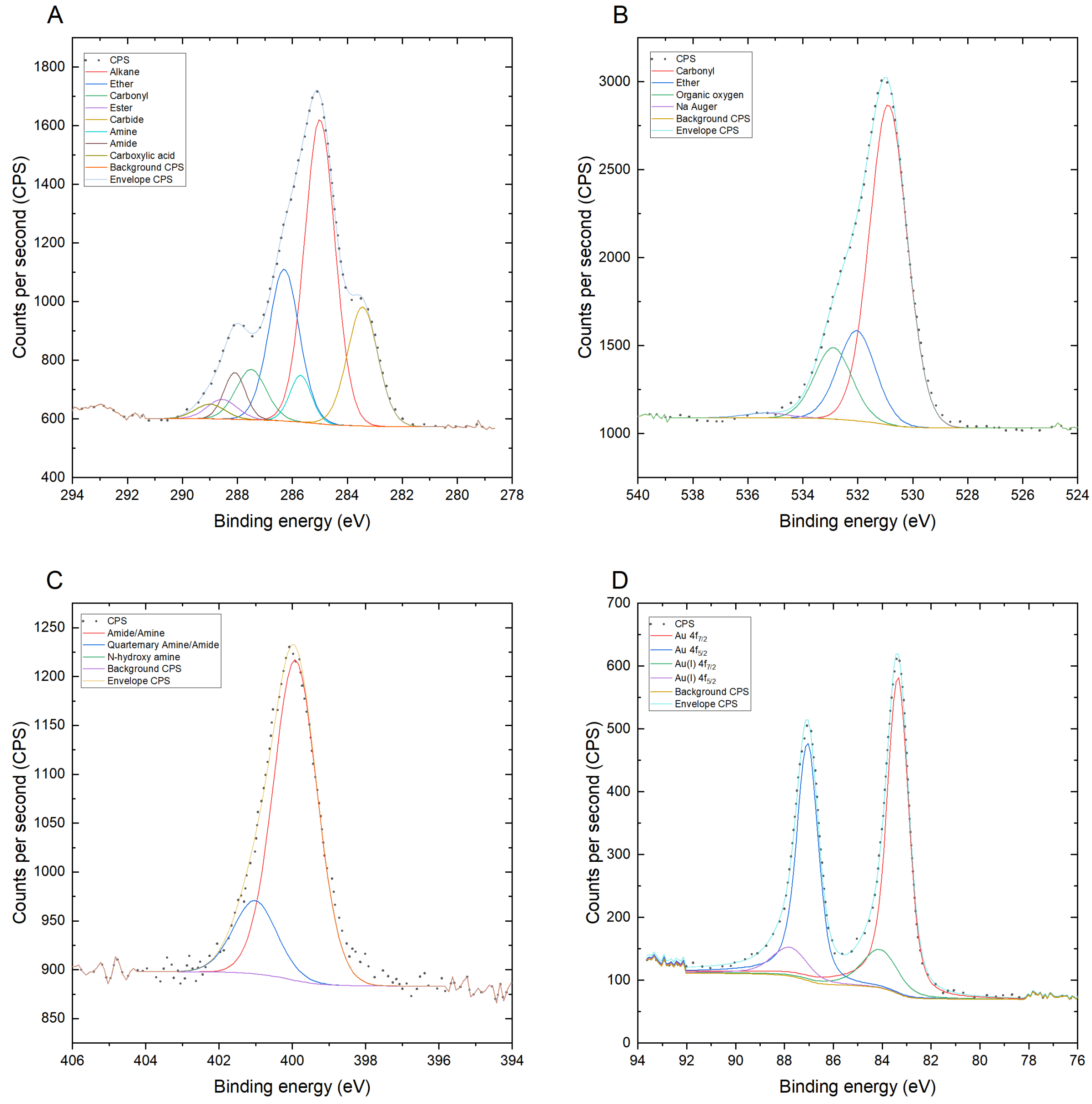

Figure S55. – XPS of GalPHEA$_{58}$@AuNP$_{16}$ A) C 1*s* B) O 1*s* C) N 1*s* and D) Au 4*f*

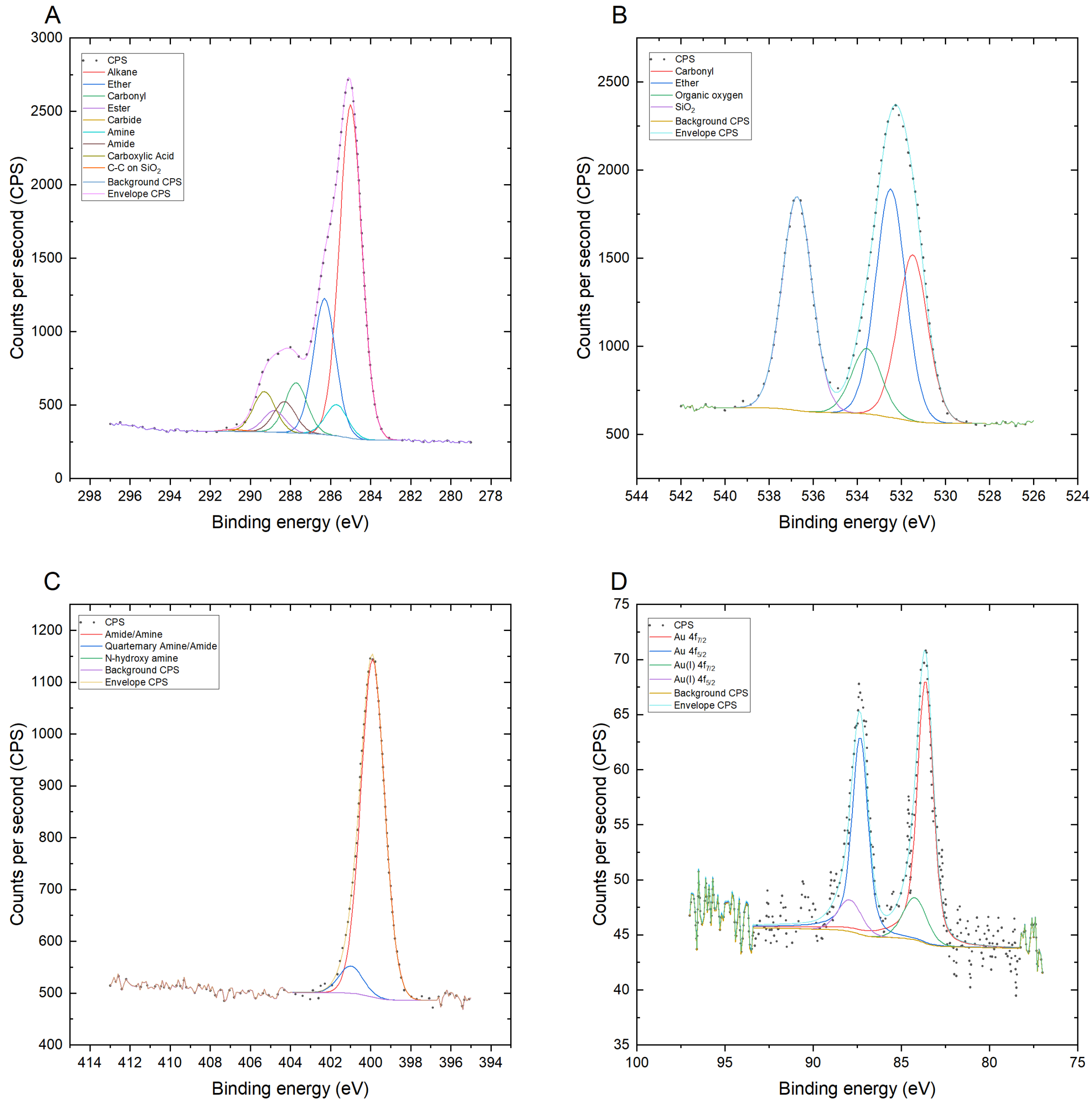

Figure S56. – XPS of GalPHEA$_{72}$@AuNP$_{16}$ A) C 1*s* B) O 1*s* C) N 1*s* and D) Au 4*f*

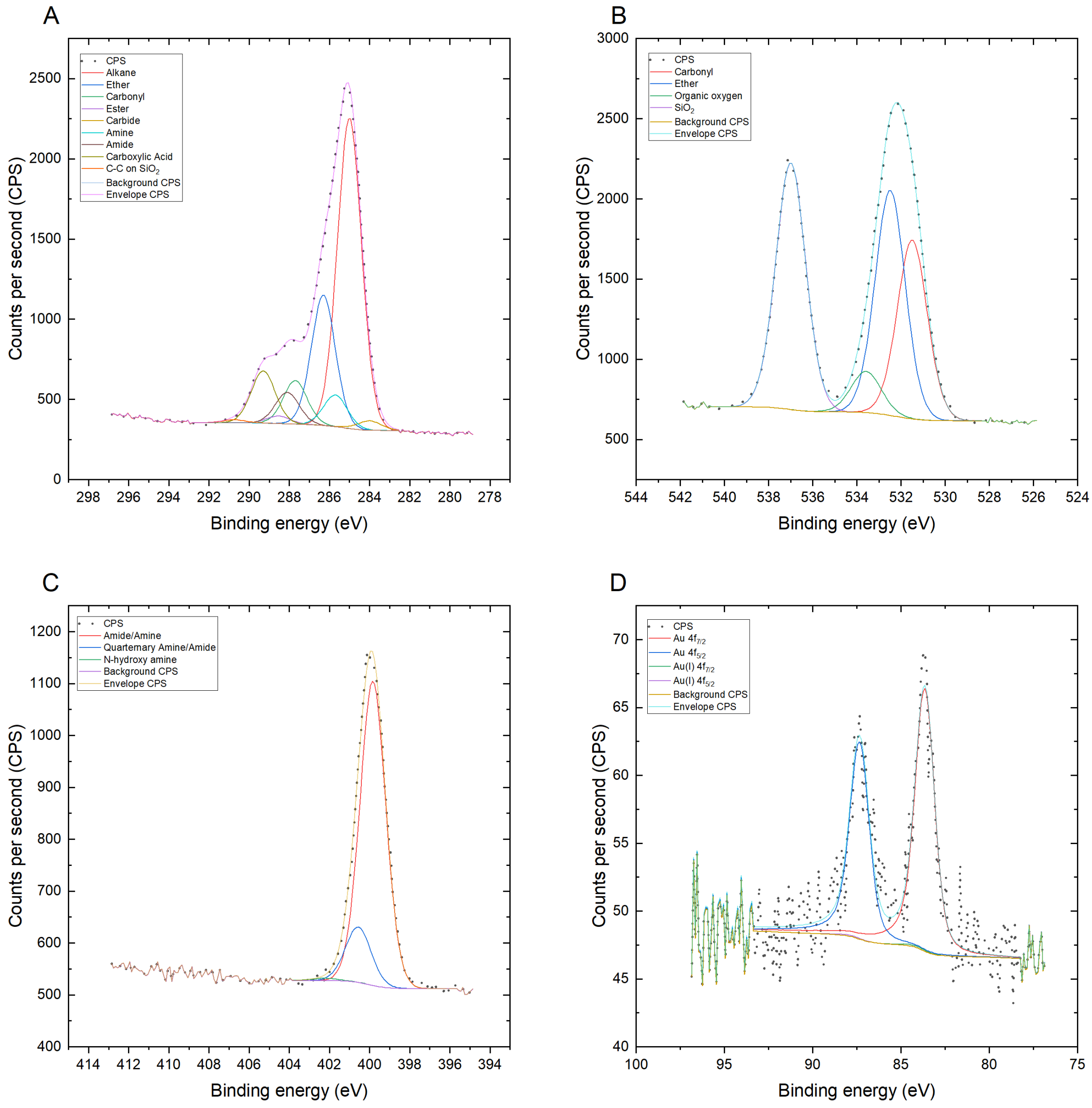

Figure S57. – XPS of GalPHEA$_{110}$@AuNP$_{16}$ A) C 1*s* B) O 1*s* C) N 1*s* and D) Au 4*f*

Particle Composition		Elemental Percentage Composition (%)				Elemental Ratios	
AuNP (nm)	PHEA DP	C 1*s*	O 1*s*	N 1*s*	Au 4*f*	N 1*s*/C 1*s*	N 1*s*/Au 4*f*
16	0	53.04	45.81	0.14	1.01	0.003	0.138
16	40	65.83	26.34	7.75	0.08	0.118	96.875
16	50	60.69	31.33	5.93	2.06	0.098	2.879
16	58	60.21	31.97	6.50	1.32	0.108	4.924
16	72	65.67	27.29	6.70	0.04	0.102	167.5
16	110	62.15	30.62	7.20	0.03	0.116	240.0

Table S20. – Elemental composition of 1-deoxy-1-amino-galactose functionalised nanoparticles determined by XPS

Particle Composition		C 1*s* Bond Percentage Composition (%)									Bond Ratios	
AuNP (nm)	PHEA DP	Alkane	Ether	Carbonyl	Ester	Carbide	Amine	Amide	Carboxylic Acid	C-C on SiO_2	Amide/ Alkane	Amide/ Ether
16	0	54.83	14.31	2.31	22.61	1.02	0.00	0.00	4.93	0.00	0.0000	0.0000
16	40	16.23	37.85	14.06	2.55	11.28	4.99	5.00	8.04	0.00	0.3075	0.1321
16	50	51.38	19.84	6.26	4.26	9.78	3.94	3.94	0.60	0.00	0.0767	0.1986
16	58	41.85	20.91	6.94	2.72	16.39	4.57	4.58	2.05	0.00	0.1092	0.2190
16	72	51.51	21.04	7.82	3.38	0.00	4.83	4.84	6.27	0.31	0.0940	0.2300
16	110	50.00	21.03	7.02	1.24	1.54	5.10	5.11	8.47	0.48	0.1022	0.2430

Table S21. – C 1*s* bonding composition of 1-deoxy-1-amino-galactose functionalised nanoparticles determined by XPS

References

(1) Schneider, C. A.; Rasband, W. S.; Eliceiri, K. W. NIH Image to ImageJ: 25 Years of Image Analysis. *Nat. Methods* **2012**, *9* (7), 671–675.

(2) Richards, S.-J.; Gibson, M. I. Optimization of the Polymer Coating for Glycosylated Gold Nanoparticle Biosensors to Ensure Stability and Rapid Optical Readouts. *ACS Macro Lett.* **2014**, *3* (10), 1004–1008.

(3) Campa, C.; Donati, I.; Vetere, A.; Gamini, A.; Paoletti, S. Synthesis of Glycosylamines: Indentification and Quantification of Side Products. *J. Carbohydr. Chem.* **2001**, *20* (3–4), 263–273.

(4) Lubineau, A.; Augé, J.; Drouillat, B. Improved Synthesis of Glycosylamines and a Straightforward Preparation of N-Acylglycosylamines as Carbohydrate-Based Detergents. *Carbohydr. Res.* **1995**, *266* (2), 211–219.

(5) Ieong, N. S.; Brebis, K.; Daniel, L. E.; O'Reilly, R. K.; Gibson, M. I. The Critical Importance of Size on Thermoresponsive Nanoparticle Transition Temperatures: Gold and Micelle-Based Polymer Nanoparticles. *Chem. Commun.* **2011**, *47* (42), 11627–11629.

(6) Bastús, N. G.; Comenge, J.; Puntes, V. Kinetically Controlled Seeded Growth Synthesis of Citrate-Stabilized Gold Nanoparticles of up to 200 Nm: Size Focusing versus Ostwald Ripening. *Langmuir* **2011**, *27* (17), 11098–11105.
